# Association between living with children and outcomes from covid-19: OpenSAFELY cohort study of 12 million adults in England

**DOI:** 10.1136/bmj.n628

**Published:** 2021-03-18

**Authors:** Harriet Forbes, Caroline E Morton, Seb Bacon, Helen I McDonald, Caroline Minassian, Jeremy P Brown, Christopher T Rentsch, Rohini Mathur, Anna Schultze, Nicholas J DeVito, Brian MacKenna, William J Hulme, Richard Croker, Alex J Walker, Elizabeth J Williamson, Chris Bates, Amir Mehrkar, Helen J Curtis, David Evans, Kevin Wing, Peter Inglesby, Henry Drysdale, Angel Y S Wong, Jonathan Cockburn, Robert McManus, John Parry, Frank Hester, Sam Harper, Ian J Douglas, Liam Smeeth, Stephen J W Evans, Krishnan Bhaskaran, Rosalind M Eggo, Ben Goldacre, Laurie A Tomlinson

**Affiliations:** 1 Electronic Health Records Research Group, Faculty of Epidemiology and Population Health, London School of Hygiene and Tropical Medicine, London, UK; 2 The DataLab, Nuffield Department of Primary Care Health Sciences, University of Oxford, Oxford OX2 6GG, UK; 3 The Phoenix Partnership, 129 Low Lane, Horsforth, Leeds, UK; 4 Centre for Mathematical Modelling of Infectious Diseases, London School of Hygiene & Tropical Medicine, London, UK

## Abstract

**Objective:**

To investigate whether risk of infection with severe acute respiratory syndrome coronavirus 2 (SARS-CoV-2) and outcomes of coronavirus disease 2019 (covid-19) differed between adults living with and without children during the first two waves of the UK pandemic.

**Design:**

Population based cohort study, on behalf of NHS England.

**Setting:**

Primary care data and pseudonymously linked hospital and intensive care admissions and death records from England, during wave 1 (1 February to 31 August 2020) and wave 2 (1 September to 18 December 2020).

**Participants:**

Two cohorts of adults (18 years and over) registered at a general practice on 1 February 2020 and 1 September 2020.

**Main outcome measures:**

Adjusted hazard ratios for SARS-CoV-2 infection, covid-19 related admission to hospital or intensive care, or death from covid-19, by presence of children in the household.

**Results:**

Among 9 334 392****adults aged 65 years and under, during wave 1, living with children was not associated with materially increased risks of recorded SARS-CoV-2 infection, covid-19 related hospital or intensive care admission, or death from covid-19. In wave 2, among adults aged 65 years and under, living with children of any age was associated with an increased risk of recorded SARS-CoV-2 infection (hazard ratio 1.06 (95% confidence interval 1.05 to 1.08) for living with children aged 0-11 years; 1.22 (1.20 to 1.24) for living with children aged 12-18 years) and covid-19 related hospital admission (1.18 (1.06 to 1.31) for living with children aged 0-11; 1.26 (1.12 to 1.40) for living with children aged 12-18). Living with children aged 0-11 was associated with reduced risk of death from both covid-19 and non-covid-19 causes in both waves; living with children of any age was also associated with lower risk of dying from non-covid-19 causes. For adults 65 years and under during wave 2, living with children aged 0-11 years was associated with an increased absolute risk of having SARS-CoV-2 infection recorded of 40-60 per 10 000 people, from 810 to between 850 and 870, and an increase in the number of hospital admissions of 1-5 per 10 000 people, from 160 to between 161 and 165. Living with children aged 12-18 years was associated with an increase of 160-190 per 10 000 in the number of SARS-CoV-2 infections and an increase of 2-6 per 10 000 in the number of hospital admissions.

**Conclusions:**

In contrast to wave 1, evidence existed of increased risk of reported SARS-CoV-2 infection and covid-19 outcomes among adults living with children during wave 2. However, this did not translate into a materially increased risk of covid-19 mortality, and absolute increases in risk were small.

## Introduction

The role of children and adolescents in the transmission of severe acute respiratory syndrome coronavirus 2 (SARS-CoV-2) is uncertain.^1^ ^2^ Good evidence indicates that they have lower susceptibility to infection and are less likely to have severe disease once infected.^1^ ^3^ ^4^ Modelling of other respiratory tract infections such as influenza suggests that children are a major driver of transmission during the initial phase of an epidemic, in part owing to a high frequency of social contacts.^5^ By contrast, accruing evidence suggests that for SARS-CoV-2 lower susceptibility and possibly lower infectiousness, particularly among younger children, means that they may not transmit infection more than adults.^6^

Proposed explanatory mechanisms for lower susceptibility or propensity to disease among children include age dependent expression of the angiotensin converting enzyme 2 gene, differences in innate and adaptive immunity, more frequent respiratory infections, and pre-existing immunity to coronaviruses.^7^ ^8^ ^9^ Four seasonal coronaviruses usually cause self-limiting “common cold”-like syndromes, although coronaviruses are only one group of viruses responsible, causing 10-30% of common colds in adults.^10^ Children have more colds each year than do adults, with the highest infection frequency in young children.^11^ ^12^ ^13^ Adults in close contact with children also have a higher frequency of viral respiratory infections, especially women and adults exposed to younger children.^14^

If recent coronavirus infection is protective against SARS-CoV-2 infection, then adults living with children may be at a lower risk than those living without children. Conversely, children, or activities associated with childcare, could mean that adults living with children may have an increased risk of exposure to SARS-CoV-2 infection. Once infection has occurred, potential cross reactive immunity, and better health among adults living with children, could also affect the severity of illness with coronavirus disease 2019 (covid-19). Therefore, quantifying the overall effect of living with children on the risk of SARS-CoV-2 infections and severe outcomes from covid-19 is important. We conducted a large cohort study using UK electronic health records with linked data on household members to determine whether the risk of infection and covid-19 outcomes differs between adults living with and without school age children.

## Methods

### Database description

Primary care records managed by the general practice software provider The Phoenix Partnership (TPP) were linked to Secondary Uses Service hospital admissions, SARS-CoV-2 testing data from the Second Generation Surveillance System, Intensive Care National Audit and Research Centre (ICNARC) covid-19-related intensive care unit (ICU) admissions,^15^ and Office for National Statistics mortality records through OpenSAFELY, a data analytics platform created on behalf of NHS England to help to answer urgent research questions related to covid-19 (https://opensafely.org).

OpenSAFELY provides a secure software interface allowing the analysis of pseudonymised primary care patient records from England in near real time within the electronic health records vendor’s highly secure data centre, avoiding the need for large volumes of potentially disclosive pseudonymised patient data to be transferred off site. This, in addition to other technical and organisational controls, minimises any risk of re-identification. Similarly, pseudonymised datasets from other data providers are securely provided to the electronic health records vendor and linked to the primary care data. The dataset analysed within OpenSAFELY is based on 24 million people currently registered with general practice surgeries and uses TPP SystmOne software. It includes pseudonymised data such as coded diagnoses, drugs, and physiological parameters. No free text data are included. An index of multiple deprivation is available, which ranks every lower layer super output area on the basis of various characteristics of the region.^16^

### Study design and population

Our pre-specified study protocol and post hoc protocol amendments (supplementary table A1) are available.^17^ We extracted two separate study populations for each wave of the pandemic. These study populations included all adults aged 18 years or over, registered in an English general practice with TPP software. The first cohort included people registered on 1 February 2020 (study start for first wave), and the second cohort included those registered on 1 September 2020 (study start for second wave). We followed participants until the earliest of development of the outcome of interest, deregistration from their general practice, death from any cause, or study end. Study end was 31 August for the first wave cohort and 18 December for the second wave cohort, except for covid-19 related hospital admissions, for which study end was 30 November 2020.

#### Inclusion and exclusion criteria

We required participants to have at least three months of follow-up before study start (1 February or 1 September). This sought to ensure that a patient’s health record had been updated following any recent change of general practice, while minimising loss of households moving home more frequently, potentially related to having children.

The pseudonymised household identifier developed by TPP links people living at the same address on 1 February 2020 (see supplementary methods). We excluded people with no household identifier and those living in care homes (derived by TPP from linking addresses matched to publicly available Care Quality Commission data) or household sizes above 10 people (possible care homes or other institutions). We excluded households in which any individual had a missing record of age, to avoid misclassifying our main exposure variable (household exposure to children). Finally, we excluded people with missing ethnicity, sex, or index of multiple deprivation.

### Study measures

#### Exposures

The primary exposure was an ordered categorical variable reflecting school stages, derived using the ages of individuals linked by the household identifier: no children under 18 years in the household, only children aged 0-11 years in the household, only children aged 12-18 years in the household, and at least one child aged 0-11 years and at least one aged 12-18 years in the household.

#### Outcomes

We included four outcomes: evidence of SARS-CoV-2 infection, defined as the earliest of either a covid-19 code recorded in primary care (specifically, a code indicating a clinical diagnosis of covid-19, a positive swab test for SARS-CoV-2, or having sequelae of covid-19 (see supplementary methods)) or having a positive covid-19 test in Second Generation Surveillance System data; hospital admission for covid-19 defined as an ICD-10 (international classification of diseases, 10th revision) code for covid-19 in the primary diagnosis field (ascertained from Secondary Uses Service data); ICU admission with covid-19 that required non-invasive or invasive respiratory support (ascertained from ICNARC data); and covid-19 related death, defined as an ICD-10 code for covid-19 anywhere on the death certificate (ascertained from Office for National Statistics death certificate data). Participants were able to develop each outcome. Retrospectively, to contextualise our findings, we added the outcome of non-covid-19 death, defined as death from any other cause on the death certificate.

In the UK, testing for SARS-CoV-2 outside of hospitals has been predominantly done among people who have developed symptoms suggestive of infection and requested a test.^18^ Availability of tests for SARS-CoV-2 have changed markedly over the period of the study. During wave 1, capacity outside hospitals was limited, so swab tests were predominantly available to people in high risk jobs or on admission to hospital. During wave 2, tests were much more readily available. In both waves, admission to hospital in the UK has been based on severity of illness and clinical need, with general guidance available about admission criteria for patients and clinicians.^19^

#### Covariates

We used a directed-acyclic graph approach to determine covariates (supplementary figure A1), considering demographics including age in years, sex, body mass index (kg/m^2^), smoking status, index of multiple deprivation, ethnicity, geographic area, and the total number of adults in the household. We identified chronic comorbidities that are associated with the risk of severe covid-19 outcomes.^20^ These are defined with links to code lists in the supplementary methods, with further information about demographic covariates. We identified participants who were recommended to shield following the UK government’s identification of clinically extremely vulnerable groups.^21^ In the main analysis, we assumed people with missing body mass index to be non-obese and those with missing smoking information to be non-smokers.

### Statistical analysis

#### Primary model

We analysed outcomes separately for adults aged 18-65 years, those most likely to be parents or primary caregivers and also of working age, and older adults (over 65 years). We described the proportion of individuals within each exposure and outcome, by the covariates. We then described the rate of outcomes according to the presence of children in the household.

We used Cox proportional hazards modelling to determine hazard ratios for each outcome, using robust standard errors to account for clustering by household identifier and stratifying by geographic area (through the Sustainability and Transformation Partnership, an NHS administrative region) to allow for geographical variation in infection rates. We analysed the association between exposure and outcome separately for each wave rather than comparing results between waves.

We wished to adjust for illness in early adult life, which could have affected the ability or decision to have children, as a confounder but were concerned about the accuracy of dates of onset of illness in the primary care record. Therefore, we used comorbidities at study start as a proxy for earlier health problems, as well as a marker of differences in current health status between adults living with and without children. To show the effect of this adjustment, we present a “demographic adjusted model,” adjusted for sex, age using a four knot cubic spline, index of multiple deprivation, body mass index, smoking, ethnicity, and total number of adults in the household, and then a “comorbidity adjusted model” with the addition of clinical comorbidities at study start. We explored violations of the proportional hazards assumption by testing for a zero slope in the scaled Schoenfeld residuals.

#### Secondary models

We examined possible interactions between our primary exposure and sex of the adult and probable shielding behaviour of the adult. In relation to the possibility cross reactive immunity from other coronavirus infections, we also examined for evidence of a “dose-response effect” of exposure to previous coronavirus infections by re-categorising the number of children, in households with only children aged 0-11 years, as one, two, three, and four or more.

#### Sensitivity analyses

To confirm that our household identifier correctly linked adults in close contact with children we did a similar analysis over an earlier time period (1 February 2019 to 1 February 2020), in which the outcome was a Read code for threadworm infection, a condition for which we would anticipate transmission from children to adults, with the strongest association seen in younger children.

Secondly, we repeated the comorbidity adjusted model: restricting to participants with complete body mass index and smoking data; using age as the underlying timescale, to ensure that we had fully adjusted for age as a confounder; requiring 12 months or more primary care follow-up before study start, to fully capture pre-existing comorbidities; fitting time interactions on covariates where evidence existed of non-proportional hazards; censoring the study period on 1 November for the outcome covid-19 related hospital admission, owing to potential delays in availability of Secondary Uses Service data; and splitting the “only children 0-11 years” group into children aged 0-4 (pre-school) and 5-11 years (primary school).

As data on occupation (related to risk of SARS-CoV-2 infection^22^) were not available, we used quantitative bias analysis to assess the potential extent of confounding from high risk occupation among adults 65 years and under. We calculated bias adjusted hazard ratios under a range of plausible assumptions about the association between occupation and risk of infection and prevalence of high risk occupations among people with and without children.^23^ ^24^ ^25^ ^26^

We carried out an additional post hoc sensitivity analysis using multiple imputation to include individuals initially excluded owing to missing ethnicity data. We used a population calibrated imputation approach, with marginal proportions of each ethnicity group within each of nine broad geographical regions of England (East, East Midlands, London, North East, North West, South East, South West, West Midlands, Yorkshire and The Humber) taken from Annual Population Survey data (pooled 2014-16). Five imputed datasets were created with estimated hazard ratios combined using Rubin’s rules.^27^

Finally, to quantify the absolute risk associated with living with children in wave 2, we estimated a range of increases in SARS-CoV-2 infections and hospital admissions with covid-19 for each exposure category by multiplying the bounds of the 95% confidence intervals by the rate of each outcome in people not living with children and expressed this per 10 000 people.

#### Software and reproducibility

We used Python 3.8 and SQL (Server 2016 Enterprise SP2) for data management and Stata 16 for analysis. The analysis code is available online.^28^

### Patient and public involvement

Neither patients nor the public were involved in developing the research question and study or in the design, management, or interpretation of this study. The primary barrier was the rapid timescale of analysis to deliver timely results.

## Results

The final cohort for wave 1 included 9 334 392 adults 65 years or under and 2 684 524 adults over 65 years (supplementary figure A2): wave 2 had similar numbers. In both waves, among people 65 years and under, 63% did not live with children, 20% lived with only children aged 0-11 years, 10% lived with only children aged 12-18 years, and 7% lived with children aged 0-11 and 12-18 years (table 1). Those living with children were more likely to be younger, female, and of non-white ethnicity, to have a lower index of multiple deprivation, to have more adults in the household, and to have fewer comorbidities (supplementary table A3). In wave 1, a total of 51 560 (0.55%) had recorded SARS-CoV-2 infection, 6374 (0.07%) were admitted to hospital with covid-19, 1601 (0.02%) were admitted to ICU for ventilatory support with covid-19, and 1219 (0.01%) died of covid-19 (supplementary table A3). In wave 2, a total of 241 693 (2.61%) had recorded SARS-CoV-2 infection, 3616 (0.04%) were admitted to hospital with covid-19, 1102 (0.01%) were admitted to ICU for ventilatory support with covid-19, and 591 (0.01%) died of covid-19 (supplementary table A3). The vast majority of diagnoses for the outcome SARS-CoV-2 infection, particularly in wave 2, came from swab testing (supplementary table A4).

**Table 1:** Cohort description, of adults 65 years and under, by presence of children in household during first and second wave of UK pandemic. Values are numbers (percentages)

Characteristics	Wave 1 (1 February to 31 August)						Wave 2 (1 September to 18 December)				
	Total cohort (n=9 334 392)	Ages of children in household					Total cohort (n=9 266 919)	Ages of children in household			
		No children (n=5 862 942)	Only children aged 0-11 (n=1 918 698)	Only children aged 12-18 (n=868 582)	Children aged 0-11 and 12-18 (n=684 170)			No children (n=5 826 609)	Only children aged 0-11 (n=1 849 103)	Only children aged 12-18 (n=895 168)	Children aged 0-11 and 12-18 (n=696 039)
Age, years:											
18-<30	1 981 860 (21.2)	1 363 510 (23.26)	370 176 (19.29)	163 895 (18.87)	84 279 (12.32)		1 912 652 (20.6)	1 315 490 (22.58)	338 068 (18.28)	172 635 (19.29)	86 459 (12.42)
30-<40	2 230 655 (23.9)	1 022 787 (17.44)	888 086 (46.29)	89 796 (10.34)	229 986 (33.62)		2 222 046 (24.0)	1 042 511 (17.89)	854 529 (46.21)	92 963 (10.38)	232 043 (33.34)
40-<50	2 022 059 (21.7)	892 381 (15.22)	493 831 (25.74)	341 432 (39.31)	294 415 (43.03)		2 021 030 (21.8)	883 126 (15.16)	491 383 (26.57)	346 632 (38.72)	299 889 (43.09)
50-<60	2 062 371 (22.1)	1 622 258 (27.67)	127 370 (6.64)	248 283 (28.58)	64 460 (9.42)		2 064 172 (22.3)	1 615 153 (27.72)	126 089 (6.82)	256 739 (28.68)	66 191 (9.51)
60-<66	1 037 447 (11.1)	962 006 (16.41)	39 235 (2.04)	25176 (2.90)	11 030 (1.61)		1 047 019 (11.3)	970 329 (16.65)	39 034 (2.11)	26 199 (2.93)	11 457 (1.65)
Female sex	4802386 (51.4)	2837737 (48.40)	1102345 (57.45)	478341 (55.07)	383963 (56.12)		4 757 515 (51.3)	2 813 198 (48.28)	1 061 462 (57.40)	492 480 (55.02)	390 375 (56.09)
Ethnicity:											
White	7 734 228 (82.9)	5 018 723 (85.60)	1 517 885 (79.11)	698 665 (80.44)	498 955 (72.93)		7 666 700 (82.7)	4 978 892 (85.45)	1 462 663 (79.10)	717 609 (80.16)	507 536 (72.92)
Mixed	160 065 (1.7)	95 418 (1.63)	35 811 (1.87)	14 439 (1.66)	14 397 (2.10)		160 932 (1.7)	96 111 (1.65)	34 747 (1.88)	15 243 (1.70)	14 831 (2.13)
South Asian	836 666 (9.0)	395 482 (6.75)	233 110 (12.15)	99 548 (11.46)	108 526 (15.86)		835 735 (9.0)	397 786 (6.83)	224 660 (12.15)	103 212 (11.53)	110 077 (15.81)
Black	309 079 (3.3)	168 273 (2.87)	68 060 (3.55)	33 380 (3.84)	39 366 (5.75)		309 730 (3.3)	169 139 (2.90)	65 419 (3.54)	35 237 (3.94)	39 935 (5.74)
Other	294 354 (3.2)	185 046 (3.16)	63 832 (3.33)	22 550 (2.60)	22 926 (3.35)		293 822 (3.2)	184 681 (3.17)	61 614 (3.33)	23 867 (2.67)	23 660 (3.40)
IMD fifth:											
1 (least deprived)	1 724 481 (18.5)	1 062 278 (18.12)	366 563 (19.10)	185 680 (21.38)	109 960 (16.07)		174 1677 (18.8)	1 074 749 (18.45)	359 760 (19.46)	193 610 (21.63)	113 558 (16.31)
2	1 850 024 (19.8)	1 196 763 (20.41)	368 768 (19.22)	172 144 (19.82)	112 349 (16.42)		1 837 273 (19.8)	1 191 807 (20.45)	354 459 (19.17)	177 150 (19.79)	113 857 (16.36)
3	1 942 791 (20.8)	1 266 447 (21.60)	381 946 (19.91)	169 873 (19.56)	124 525 (18.20)		1 898 184 (20.5)	1 238 423 (21.25)	362 430 (19.60)	172 020 (19.22)	125 311 (18.00)
4	2 010 621 (21.5)	1 273 190 (21.72)	409 140 (21.32)	174 424 (20.08)	153 867 (22.49)		1 994 633 (21.5)	1 263 543 (21.69)	394 553 (21.34)	179 832 (20.09)	156 705 (22.51)
5 (most deprived)	180 6475 (19.4)	1 064 264 (18.15)	392 281 (20.45)	166 461 (19.16)	183 469 (26.82)		1 795 152 (19.4)	1 058 087 (18.16)	377 901 (20.44)	172 556 (19.28)	186 608 (26.81)
≥3 adults in household	3 223 340 (34.5)	2 026 229 (34.56)	527 633 (27.50)	415 617 (47.85)	253 861 (37.10)		3 270 235 (35.3)	2 051 056 (35.20)	516 672 (27.94)	439 632 (49.11)	262 875 (37.77)
Probable shielding*	1 976 741 (21.2)	1 289 269 (21.99)	373 102 (19.45)	180 874 (20.82)	133 496 (19.51)		1 94 4911 (21.0)	1 267 606 (21.76)	358 550 (19.39)	183 976 (20.55)	134 779 (19.36)
Any comorbidity†	2 829 678 (30.3)	1 885 989 (32.17)	495 963 (25.85)	259 559 (29.88)	188 167 (27.50)		2 782 512 (30.0)	1 853 883 (31.82)	476 053 (25.75)	263 308 (29.41)	189 268 (27.19)

Full information on cohort is in supplementary table A3.

IMD=index of multiple deprivation.

* Includes people with organ transplants, renal replacement therapy, haematological cancers, non-haematological cancers, immunodeficiencies/asplenia, and severe respiratory conditions.

† Includes chronic respiratory disease, asthma, chronic cardiac disease, diabetes, cancer, end stage renal disease, chronic liver disease, stroke or dementia, other neurological disease, other transplant, asplenia, and rheumatoid arthritis, systemic lupus erythematosus, psoriasis, or other immunosuppressive condition.

Among people over 65 years, 97% did not live with children (supplementary table A2). In wave 1, a total of 18 893 (0.70%) had recorded SARS-CoV-2 infection, 8560 (0.32%) were admitted to hospital with covid-19, 732 (0.03%) were admitted to ICU for ventilatory support with covid-19, and 6507 (0.24%) died of covid-19 (supplementary table A5). In wave 2, a total of 34 519 (1.28%) had recorded SARS-CoV-2 infection, 4280 (0.16%) were admitted to hospital with covid-19, 784 (0.03%) were admitted to ICU for ventilatory support with covid-19, and 4108 (0.15%) died of covid-19 (supplementary table A5).

### Adults aged 65 years and under

**Fig 1:**
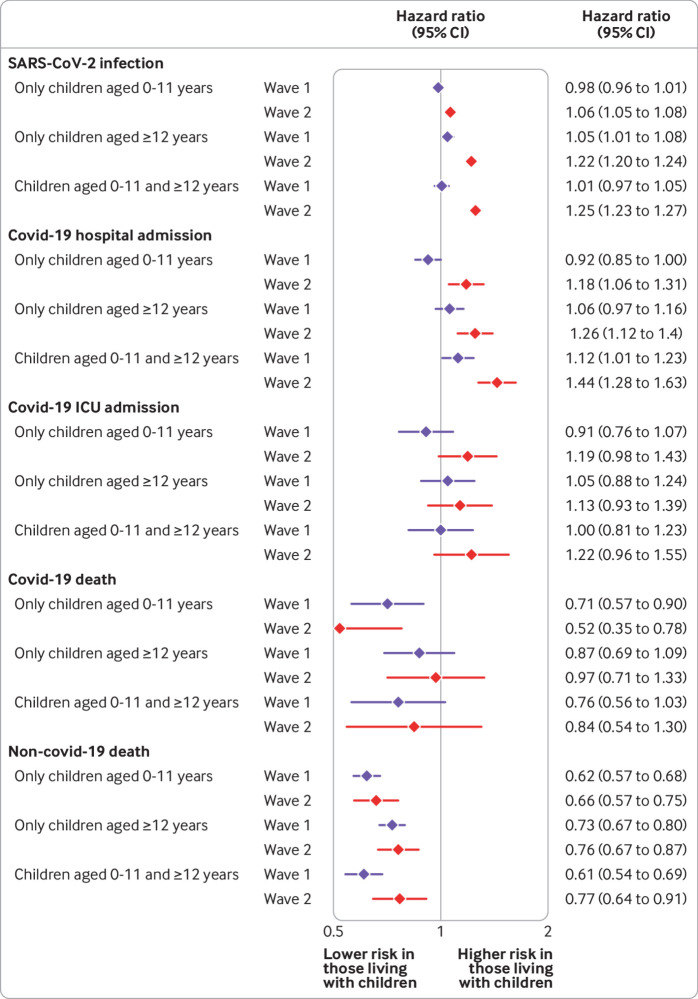
Adjusted hazard ratios for outcomes recorded SARS-CoV-2 infection, covid-19 related hospital admission, covid-19 related intensive care unit (ICU) admission, covid-19 related death, and non-covid-19 related death among adults aged 65 years and under, for waves 1 and 2 of UK pandemic. Comorbidity adjusted model adjusted for age, sex, ethnicity, number adults in household, index of multiple deprivation, body mass index, smoking, hypertension or high blood pressure, chronic respiratory disease, asthma, cancer, chronic liver disease, stroke or dementia, other neurological disease, reduced kidney function, end stage renal disease, solid organ transplant, asplenia, and rheumatoid arthritis, systemic lupus erythematosus, psoriasis, or other immunosuppressive condition

In wave 1, among adults 65 years and under, in the comorbidity adjusted model, living with children of any age was not associated with an increased risk of recorded SARS-CoV-2 infection or covid-19 related outcomes, compared with people not living with children, except for small increases in the risk of infection for adults living with children aged 12-18 years and of covid-19 related hospital admissions for adults living with children aged 0-11 and 12-18 years (fig 1). In wave 2, living with children was associated with an increased risk of recorded SARS-CoV-2 infection and covid-19 related hospital admission but was not associated with ICU admission. Living with children aged 0-11 years was associated with a reduced risk of death from covid-19 in both wave 1 and wave 2; we observed no increase in risk of death for those living with older children.

Our findings with regard to the second wave for adults 65 years and under can be contextualised by considering the absolute increase in risk of outcomes that they represent. Using the overall rate of outcomes among people living without children as the baseline, we estimate that living with children aged 0-11 years was associated with an increase in the number of SARS-CoV-2 infections of 40-60 per 10 000 people, from 810 to between 850 and 870, and an increase in the number of hospital admissions of 1-5 per 10 000 people, from 160 to between 161 and 165. Living with children aged 12-18 years was associated with an increase of 160-190 per 10 000 in the number of SARS-CoV-2 infections and an increase of 2-6 per 10 000 in the number of hospital admissions.

### Adults aged over 65 years

For adults over 65 years living in a household with children, we found no evidence of an association with any outcome in wave 1 (supplementary figure A4). In wave 2, living with children of any age was associated with an increased risk of recorded SARS-CoV-2 infection but not covid-19 related hospital admission. We also found evidence that living with children aged 0-11 and 12-18 years was associated with an increased risk of covid-19 related ICU admission (hazard ratio 1.86, 95% confidence interval 1.11 to 3.14) and covid-19 related death (1.44, 1.05 to 1.97).

### Control analyses and interactions

In relation to the control outcome of death from non-covid-19 related causes, in both waves, living with children of any age was associated with around a 30% reduced risk for adults 65 or under, but risk was not reduced for adults over 65 years (fig 1; supplementary figure A4). We observed no strong or consistent trends in the associations between risks of recorded SARS-CoV-2 infection or severe outcomes from covid-19 and the number of children aged 0-11 years in a household, for adults of any age in either wave (supplementary table A6).

**Fig 2:**
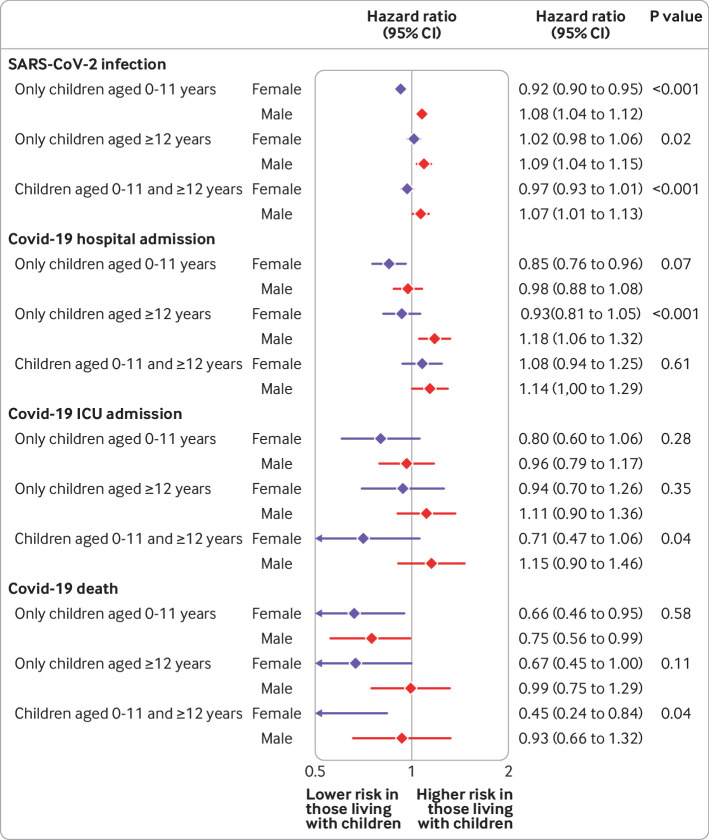
Comorbidity adjusted hazard ratios for each covid-19 outcome, compared with having no children in household, by sex, among adults aged 65 years and under for wave 1 of UK pandemic

**Fig 3:**
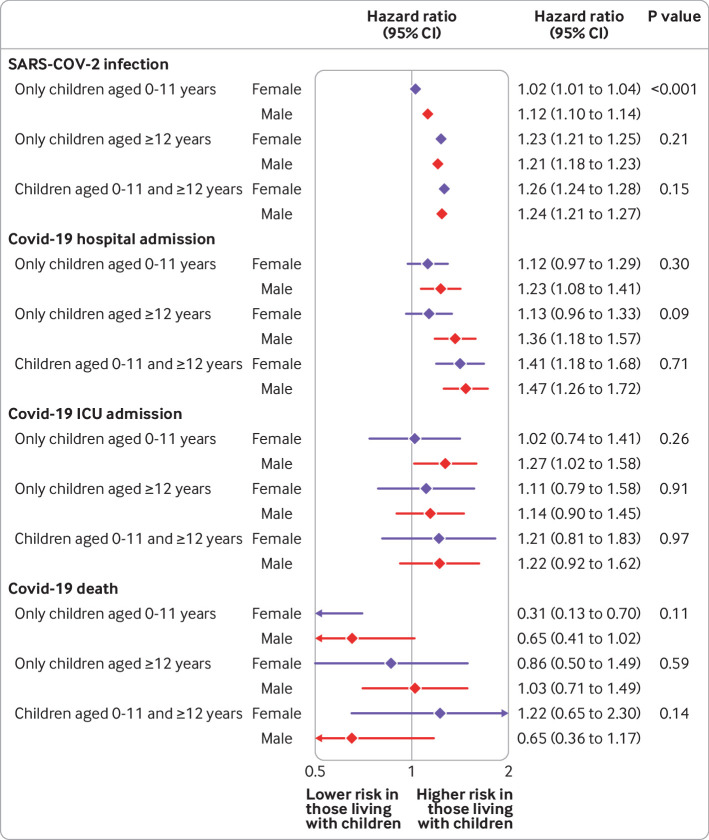
Comorbidity adjusted hazard ratios for each covid-19 outcome, compared with having no children in household, by sex, among adults aged 65 years and under for wave 2 of UK pandemic

**Fig 4:**
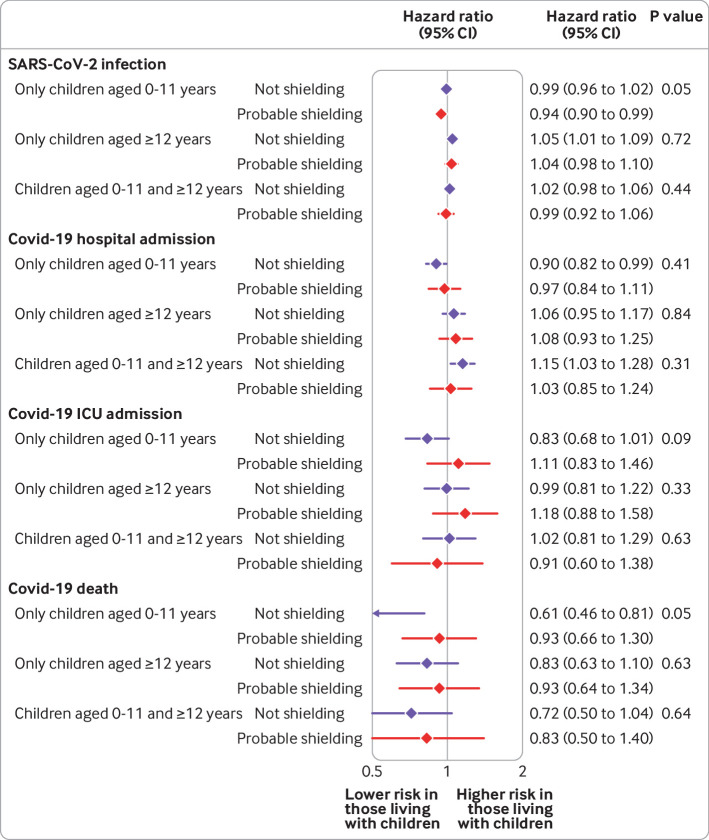
Comorbidity adjusted hazard ratios for each covid-19 outcome, compared with having no children in household, by shielding status, among adults aged 65 years and under for wave 1 of UK pandemic

**Fig 5:**
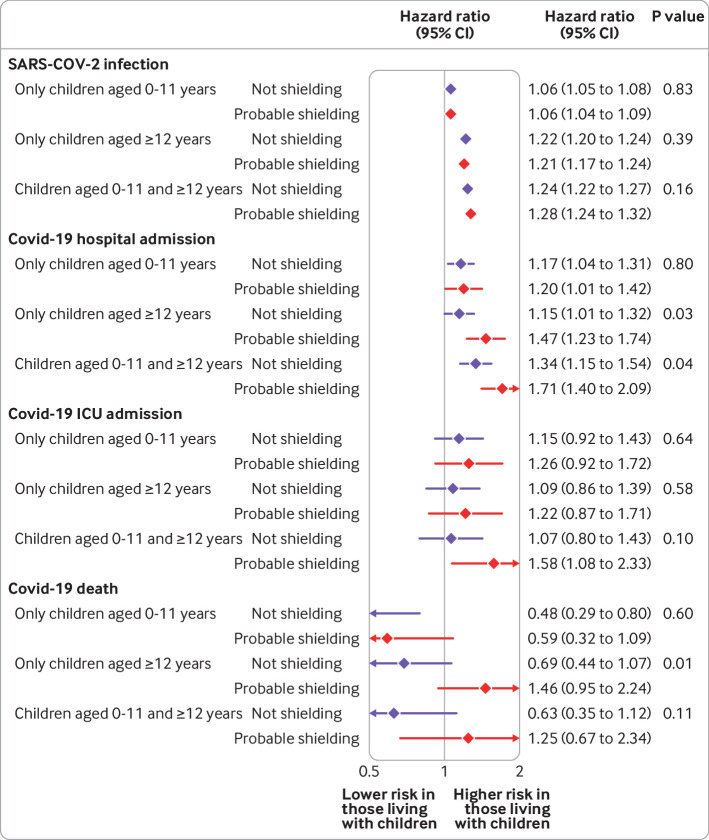
Comorbidity adjusted hazard ratios for each covid-19 outcome, compared with having no children in household, by shielding status, among adults aged 65 years and under for wave 2 of UK pandemic

We explored whether the association between household exposure to children and the risk of covid-19 outcomes varied by sex or probable shielding status in the comorbidity adjusted model (fig 2, fig 3, fig 4, and fig 5 for adults 65 years and under; supplementary figure A5 for adults over 65 years). Among adults 65 years and under, we observed no consistent patterns of interaction by sex, although in both waves the small increase in risk of recorded SARS-CoV-2 infection among those living with children aged 0-11 years was observed in men but not women (P for interaction<0.001). For adults over 65 years, we observed a greater increase in risk of recorded SARS-CoV-2 infection and covid-19 related hospital admission for women living with children 0-11 and 12-18 years than for men.

We found no evidence of important increases in risk for any outcome for potentially shielding adults 65 years and under in wave 1. However, in wave 2, for people living with children aged 12-18 years or with children aged 0-11 and 12-18 years, the magnitude of the increased risk of covid-19 related hospital admission was greater in those potentially shielding than those not shielding (P for interaction=0.03 and 0.04, respectively). For death from covid-19, for people living with children 12-18 years, we found weak evidence of an increased risk in those who were shielding (hazard ratio 1.46, 0.95 to 2.24) but not in those who were not shielding (0.69, 0.44 to 1.07; P for interaction=0.009).

### Model checking and sensitivity analyses

Among adults 65 years and under, living with only children aged 0-11 years was associated with a twofold higher risk of being diagnosed with threadworm (hazard ratio 2.70, 2.33 to 3.12), with strong evidence of an increased risk with increasing number of children aged 0-11 in the household. The risk was also increased among adults living with only children aged 12-18 years, although the increase was of a smaller magnitude (hazard ratio 1.30, 1.02 to 1.67) (supplementary table A7).

None of the sensitivity analyses materially altered the results from the comorbidity adjusted models (supplementary figure A6). We detected evidence of non-proportional hazards in the comorbidity adjusted models for all outcomes (P<0.001). Further assessment for non-proportional hazards based on Schoenfeld residuals showed evidence of non-proportionality by exposure status for reported SARS-CoV-2 infection in both waves and for covid-19 related ICU admission in wave 1. For all outcomes, we also found evidence of non-proportional hazards in several adjustment covariates. Therefore, we did a sensitivity analysis fitting a time interaction (at 1 April and 1 May 2020 for wave 1; at 1 November and 1 December in wave 2) with these covariates; the results for the main exposure from these models were similar to the main analysis (supplementary figure A6).

In post hoc analysis, we observed an increased risk of recorded SARS-CoV-2 infection and covid-19 related hospital admission for adults 65 years and under living with preschool children aged 0-4 years (supplementary figure A7), with magnitudes similar to the findings for the 5-11 age group. When we accounted for missing ethnicity data through multiple imputation, the associations between living with children and outcomes remained the same as in the primary (complete case) analysis (supplementary tables A8 and A9). However, in wave 2, the imputed results showed stronger evidence of an increased risk of covid-19 related ICU admission for adults living with children of all ages.

In quantitative bias analysis, accounting for shared risk of people with young children working in occupations with a high risk of SARS-CoV-2 exposure, when we assumed an association between being in a high risk occupation and developing each outcome of 1.3 or 2, the hazard ratio for the association between having children and the outcomes were lowered but the overall conclusions were largely unchanged (supplementary table A10 and A11). By contrast, when we assumed a hazard ratio between high risk occupation and each outcome of 3, the differences were more marked (supplementary table A12). In wave 2, rather than an increased risk of reported SARS-CoV-2 infection among adults living with only children aged 0-11 years, we observed a reduced risk (hazard ratio 0.94, 0.93 to 0.95). Risks of reported SARS-CoV-2 infection among adults living with only children aged 12-18 years, as well as adults living with children aged 0-11 and 12-18 years, were still increased. For covid-19 related hospital admission, the hazard ratio reduced toward the null, and we found evidence of an increased risk of admission only among adults living with children aged 0-11 and 12-18 years.

## Discussion

During the first wave of the pandemic of SARS-CoV-2 in the UK, we found no evidence of substantial increases in risk of recorded infection or serious covid-19 outcomes for adults aged 65 years and under sharing a household with children of any age, compared with those living without children. However, our results show an increased risk of recorded infection and covid-19 related hospital admission for adults living with children of all age groups in the second wave. In a post hoc analysis, separating children aged 0-11 years, we found that these increased risks were similar for adults living with children of preschool and primary school age children. For adults aged over 65 years, we also found an increased risk of infection associated with living with children of any age and of ICU admission and death from covid-19 for those living with children aged 0-11 and 12-18 years. In both waves, among adults aged 65 years and under, sharing a household with children aged 0-11 years was associated with a reduced risk of death from covid-19; this was similar in magnitude to the reduction in risk of death from causes other than covid-19 seen in adults aged 65 years and under living with children of all ages.

### Strengths and limitations of study

Our analysis has several important strengths. Firstly, our study is large, providing us with sufficient power to examine rarer outcomes. Secondly, we studied SARS-CoV-2 infection, with the vast majority of cases confirmed by swab testing, and covid-19 related clinical outcomes, enabling us to examine associations across the range of severity of illness. Thirdly, we have done a control analysis of threadworm infection in adults, which provides strong evidence that our model is able to detect an infection transmitted from children to adults, with, as we would anticipate, risk increasing with the number of children in a household and with the strongest association seen in younger children. Finally, we have used the depth of linked UK healthcare and electronic health records datasets to enable detailed adjustment for a range of covariates and provide rapid evidence related to wave 2 of the covid-19 pandemic.

The study also has some limitations. Owing to geographical variation in the choice of electronic health record system, our large population may not be fully representative of the UK. We stratified by region through the Sustainability and Transformation Partnership, but we could not account for local variation in the prevalence of SARS-CoV-2. We did not examine the direct association between children and adults within a household testing positive for SARS-CoV-2, as a substantial proportion of infections in this age group will be asymptomatic or undiagnosed.^29^ In relation to the data used to define the outcomes, changing availability of SARS-CoV-2 testing over the period of the study means that results related to testing during wave 1 could have been biased by characteristics of people in high risk jobs. Additionally, results for the infection outcome could have been biased if people living with children were more likely to seek testing, although we saw similar proportions of infections defined by swab testing in those living with and without children, and we would not anticipate that this would create bias for the other outcomes. Data on hospital admissions are not available until after a patient has been discharged and thus may be biased towards capturing shorter admissions for the later part of wave 2, although early censoring of Secondary Uses Service data in a sensitivity analysis did not change our findings. The threshold for hospital treatment may have changed between wave 1 and wave 2, with increased awareness of the risks of hypoxia and availability of treatments such as dexamethasone. However, we would not anticipate this having a substantial effect on admissions for people aged 65 years and under, and we would have anticipated lower admission rates among adults living with children owing to lower levels of comorbidities. ICNARC data do not capture patients admitted to all ICUs, particularly for the newer “surge” capacity.

Occupation was an unmeasured confounder both in terms of exposure to SARS-CoV-2 (such as healthcare workers and other high risk workers) and degree of contact with children outside the home (such as nursery workers). We sought to explore the potential effect of lack of occupational information on our results through a quantitative bias analysis. This suggested that our findings were not materially altered unless a substantial proportion of the population were both more likely to live with children and to work in roles strongly associated with an increased risk of SARS-CoV-2 infection. However, our highest assumed estimate for risk of infection, taken from a study of healthcare workers,^22^ is likely to be a substantial overestimate for the average increased risk for all key workers. We were not able to adjust for confounding by previous comorbidities that affected both ability or choice to have children and subsequent risk of development of severe outcomes from covid-19. However, results of models with and without adjustment for baseline comorbidities show no important differences.

We have probably misclassified the degree of contact with children in some situations, such as for divorced parents, and limitations in the data may mean misclassification of the number of people living in a household—for example, when people have not updated their address with their general practice following a house move. In our main analysis, we have not adjusted for the number of children living in a household; therefore, our analysis represents the overall association of living with children in that age group, and we acknowledge that risk may vary by the number of children. However, we did not detect strong evidence of higher risk for adults living with increasing numbers of younger children. Finally, we assume a constant relation for infections between people through clustering at the household level, rather than detailed modelling of how infections are transmitted within households.

### Comparison with other studies

One recent cohort study has adopted a similar approach to ours to explore potential cross immunity from other coronavirus infections among people living with young children. This study linked information on more than 300 000 healthcare workers and other adults in their household in Scotland to determine whether the risk of SARS-CoV-2 infection and severe outcomes from covid-19 differed between adults living in households with and without children aged 0-11 years.^30^ During the period from March to October 2020, they found a reduced risk of testing positive for SARS-CoV-2 for those living with young children but no difference in risk of covid-19 requiring hospital admission. Differences in power, greater and more consistent risk of occupational exposure among healthcare workers in their cohort, and different epidemic trajectories between Scotland and England make direct comparisons between our results difficult. However, our results show no evidence that cross reactive immunity protects against SARS-CoV-2 infection and covid-19 outcomes in the general population.

### Policy implications

Given this, what are the factors likely to underlie our findings, and in particular the difference in results between waves 1 and 2? An important difference is that for most of the time period of wave 1, schools were closed except to children of key workers and vulnerable children. However, they reopened after the holidays at the beginning of September and remained open throughout the period of wave 2 in this study. Data from population based studies show that the reopening of schools was temporally associated with progressively increasing prevalence of SARS-CoV-2 among children of all ages.^31^ ^32^ The role of schools in transmission of SARS-CoV-2 and the effect of school closures on its prevalence remain uncertain. Our finding of an increased risk to adults who live with children beyond SARS-CoV-2 transmission within schools during wave 2 may have been partly driven by indirect effects—for example, school opening may have allowed parents to travel to work outside the home, increasing potential contacts. Potential differences also exist in the behaviours of households with children that also lead to increased social contacts, such as the need for child care, greater likelihood of play and activity outside of schools, and more frequent shopping. Furthermore, policy decisions around schools must take into account the potential harms associated with school closures, such as adverse mental health consequences and worsening inequalities.^33^ ^34^ ^35^

Although even covid-19 infection that does not lead to hospital admission may be associated with serious sequelae, that we have not found a materially increased risk of ICU admission or death overall among adults aged 65 years and under living with children is reassuring. However, signals of harm are apparent in some subgroups, and confidence intervals are compatible with an increase in risk, particularly given limited numbers of ICU admissions. Our findings of no increase in risk of severe outcomes from covid-19 despite an increased risk of infection and hospital admission could be explained by confounding if adults living with children had a greater risk of infection in wave 2 but are healthier than people without children. This explanation is supported by our finding of a substantially lower risk of mortality from causes other than covid-19 among adults aged 65 years and under living with children of any age. Parents are known to have lower all cause mortality than people without children.^36^ ^37^ The protective mechanisms of having children are likely to be multifactorial and may include healthier behaviours among parents—for example, in relation to smoking and alcohol,^38^ ^39^ self-selection of healthier people becoming parents,^40^ and beneficial changes in immune function from exposure to young children.^41^ In this context, we did not observe a lower risk of death from covid-19 for adults aged 65 years and under, compared with adults living without children, except for those living with children aged 0-11 years.

### Conclusions

During the first wave of the UK pandemic, for adults 65 years and under living with children, we found no evidence of a markedly increased risk of SARS-CoV-2 infection or severe covid-19 outcomes. However, we found evidence of increased risk of infection and hospital admission with covid-19 for adults living with children during wave 2, although the absolute increases in risk were low. These increased risks during wave 2 were observed at a time when schools remained open, raising the possibility that widespread school attendance may have led to increased risks to households, but other differences between households with and without children could also have explained these observational findings. Close monitoring and evaluation as schools re-open will be crucial to inform ongoing policy.

## What is already known on this topic

Adults living with children have more “common colds” than do those not living with children
This could result in a lower risk of serious outcomes from SARS-CoV-2 infection due to cross protective immunity from other seasonal coronaviruses
Alternatively, living with children may lead to greater opportunities for infection with SARS-CoV-2 and increased risks to adults they live with

## What this study adds

During the first wave of the pandemic in the UK, living with children of any age was not associated with an increased risk of severe outcomes from covid-19, compared with not living with children
Risk of SARS-CoV-2 infection and covid-19 related hospital admission was increased for adults aged 65 years and under living with children of any age during the second wave, compared with those not living with children
Absolute increases in risks of SARS-CoV-2 infection and covid-19 related hospital admission among adults living with children in wave 2 were small

## Web extra

Extra material supplied by authors

Web appendix: Supplementary materials

## Notes

We are very grateful for all the support received from the technical operations team of The Phoenix Partnership (TPP) throughout this work and for generous assistance from the information governance and database teams at NHS England/NHSX.

Contributors: HF, CEM, and SB contributed equally to this work as first authors. BG and LT contributed equally to this work as senior authors. HF, LT, CEM, HIM, CM, JPB, LS, SJWE, KB, RME, and BG were involved in conceptualisation of the study. HF, KB, CEM, EJW, AS, RM, CTR, AJW, CB, JC, CM, WJH, BM, SB, HJC, and RM were involved in data curation. HF, KB, and JPB were responsible for formal analysis. LS, BG, and RME acquired funding. HF, LT, CEM, and SB were involved the analysis. HF, LT, RM, AS, CTR, KB, KW, RME, LS, BG, BM, EJW, HJC, HIM, SJWE, CEM, and JPB developed the methods. HF, RM, LT, AS, AJW, CM, BG, WJH, SB, AM, CEM, HD, and KW developed code lists. HF, LT, AS, AJW, CEM, BG, and WJH were responsible for project administration. CB, JC, BG, BM, SB, and AM obtained resources. CB, JC, DE, PI, CEM, WJH, SB, HJC, RC, JP, FH, SH, and ND developed the software. HF and SB were involved in data visualisation. HF and LT wrote the original draft of the manuscript, and all authors were involved in reviewing and editing it. CB LS, BG, and AM were responsible for information governance. HF, CEM, KB, CB, and JC were involved in verification of the underlying data. The corresponding author attests that all listed authors meet authorship criteria and that no others meeting the criteria have been omitted. HF, LT, LS, and BG are the guarantors.

Ethical approval: This study was approved by the Health Research Authority (REC reference 20/LO/0651) and by the LSHTM Ethics Board (reference 21863).

Information governance: NHS England is the data controller, TPP is the data processor, and the key researchers on OpenSAFELY are acting on behalf of NHS England. This implementation of OpenSAFELY is hosted within the TPP environment, which is accredited to the ISO 27001 information security standard and is NHS IG Toolkit compliant.^42^ ^43^ Patient data have been pseudonymised for analysis and linkage using industry standard cryptographic hashing techniques; all pseudonymised datasets transmitted for linkage onto OpenSAFELY are encrypted; access to the platform is via a virtual private network connection, restricted to a small group of researchers; the researchers hold contracts with NHS England and access the platform only to initiate database queries and statistical models; all database activity is logged; only aggregate statistical outputs leave the platform environment following best practice for anonymisation of results such as statistical disclosure control for low cell counts.^44^ The OpenSAFELY research platform adheres to the obligations of the UK General Data Protection Regulation (GDPR) and the Data Protection Act 2018. In March 2020 the Secretary of State for Health and Social Care used powers under the UK Health Service (Control of Patient Information) Regulations 2002 (COPI) to require organisations to process confidential patient information for the purposes of protecting public health, providing healthcare services to the public and monitoring and managing the covid-19 outbreak and incidents of exposure.^45^ Taken together, these provide the legal bases to link patient datasets on the OpenSAFELY platform. General practices, from which the primary care data are obtained, are required to share relevant health information to support the public health response to the pandemic, and have been informed of the OpenSAFELY analytics platform.

Data sharing: All data were linked, stored, and analysed securely within the OpenSAFELY platform (https://opensafely.org/). Detailed pseudonymised patient data are potentially re-identifiable and therefore not shared. The OpenSAFELY data analysis platform was rapidly delivered without prior funding to deliver timely analyses on urgent research questions in the context of the global covid-19 health emergency. Now that the platform is established, a formal process is being developed for external users to request access in collaboration with NHS England. Details of this process will be published shortly on the OpenSAFELY website.

Code availability: Data management was performed using Python 3.8 and SQL, with analysis carried out using Stata 16.1 and Python. All code is shared openly for review and reuse under an MIT open license. All code for data management and analysis is archived online at https://github.com/opensafely/school-age-children-and-covid2. All clinical and medicines code lists are openly available for inspection and reuse at https://codelists.opensafely.org/.

The lead authors affirm that this manuscript is an honest, accurate, and transparent account of the study being reported; that no important aspects of the study have been omitted; and that any discrepancies from the study as planned (and, if relevant, registered) have been explained.

Dissemination to participants and related patient and public communities: We will share information and interpretation of our findings through press releases, social media channels, and plain language summary on the OpenSAFELY website (https://opensafely.org/research/).

Provenance and peer review: Not commissioned; externally peer reviewed.
