# Association between living with children and outcomes from covid-19: OpenSAFELY cohort study of 12 million adults in England

**DOI:** 10.1136/bmj.n794

**Published:** 2021-03-22

In this paper by Harriet Forbes and colleagues (BMJ 2021;372:n628, doi:10.1136/bmj.n628), the line of no effect was incorrectly labelled as 0 instead of 1 in all the forest plots (figs 1-5). The online version has been amended.

