# Supplementary material for: Association between living with children and outcomes from covid-19: OpenSAFELY cohort study of 12 million adults in England
Source: BMJ. 2021 Mar 18;372:n628. doi: 10.1136/bmj.n628 (PMC7970340; doi:10.1136/bmj.n628)
Supplement: Supplementary file 1 — Web appendix: Supplementary materials [file forh062967.ww1.pdf]

Contents

Figures 3

Figure A1: Directed acyclic graph (DAG) illustrating implicitly assumed causal structure between household exposure to children and recorded SARS-CoV-2 infection, and severe outcomes from COVID-19 3

Figure A2: Flow diagram of cohort with numbers excluded at different stages for wave 1 and wave 2........ 4

Figure A3: Histograms of frequencies of outcomes (recorded SARS-CoV-2 infection, COVID-19 outcomes and non-COVID-19 deaths) in wave 1 (1st February to 31st August 2020) and wave 2 (1st September to 18th December)........ 5

Figure A4: Adjusted* Hazard Ratios (HRs) for outcomes ((a) recorded SARS-CoV-2 infection, (b) COVID-19 hospital admission, (c) COVID-19 ICU admission, (d) COVID-19 death and (e) non-COVID-19 death) among adults aged >65 years, for waves 1 and 2 of the UK pandemic... 6

Figure A5. Comorbidity adjusted hazard ratios (HRs) and 95% confidence intervals (CI) for each COVID-19 outcome in wave 1 and wave 2, compared to having no children in the household by (a) sex, (b) shielding status among those >65 years. 7

Figure A6: Results from sensitivity analysis: Hazard Ratios (HRs) for each COVID-19 outcome (a) evidence of SARS-CoV-2 infection recorded in primary care, (b) COVID-19 hospital admission, (c) COVID-19 ICU admission and (d) COVID-19 death), among those ≤65 years 9

Figure A7: Results from sensitivity analysis creating alternative exposure definition which identifies children aged 0-4 years: Hazard Ratios (HRs) for each COVID-19 outcome (a) among those ≤65 years (b) among those >65 years 13

Tables........ 15

Table A1: Changes to the original study protocol 15

Table A2. Cohort description, of adults >65 years, by presence of children in the household during Wave 1 and Wave 2 of the pandemic 17

Table A3. Cohort description by outcomes (evidence of SARS-CoV-2 infection recorded in primary care, COVID-19 outcomes and non-COVID 19 deaths) for adults ≤65 years in wave 1 and wave 2 of the UK pandemic........ 22

Table A4. Source of diagnoses for the outcome of SARS-CoV-2 infection by wave and exposure status........ 26

Table A5. Cohort description by outcomes (evidence of SARS-CoV-2 infection recorded in primary care, COVID-19 outcomes and non-COVID 19 deaths) for adults >65 years in wave 1 and wave 2 of the UK pandemic 27

Table A6. Hazard Ratios (HRs) for outcomes in wave 1 and wave 2 (a) evidence of SARS-CoV-2 infection recorded in primary care, (b) COVID-19 hospital admission, (c) COVID-19 ICU admission, (d) COVID-19 death and (e) non-COVID-19 death), stratified by age. 32

Table A7. Hazard Ratios (HRs) for threadworm between 2018 to 2019, stratified by age 37

Table A8: Hazard Ratios (HRs) and 95% confidence intervals (CIs) for COVID-19 outcomes among those ≤65 years, comparing results of complete case analysis with multiple imputation to account for ethnicity. 38

Table A9: Hazard Ratios (HRs) and 95% confidence intervals (CIs) for COVID-19 outcomes among those >65 years, comparing results of complete case analysis with multiple imputation to account for ethnicity 39

Table A10: Bias-adjusted hazard ratios accounting for high-risk occupation for the association between living in a household with children and Covid-19 outcome among adults aged ≤ 65 years assuming a hazard ratio between high-risk occupation and outcome of 1.3..... 40

Table A11: Bias-adjusted hazard ratios accounting for high-risk occupation for the association between living in a household with children and Covid-19 outcomes among adults aged ≤ 65 years assuming a hazard ratio between high-risk occupation and outcome of 2 41

Table A12: Bias-adjusted hazard ratios accounting for high-risk occupation for the association between living in a household with children and Covid-19 outcomes among adults aged ≤65 years assuming a hazard ratio between high-risk occupation and outcome of 3 42

Supplementary methods 43

Algorithm to identify households in TPP, using addresses registered in patient record 43

Table of primary care codes used as evidence of SARS-CoV-2 infection in primary care with links to codelists 44

Table of covariate definitions 44

Table of chronic comorbidities with details and links to codelists 45

Further details of quantitative bias analysis 48

Figures

Figure A1: Directed acyclic graph (DAG) illustrating implicitly assumed causal structure between household exposure to children and recorded SARS-CoV-2 infection, and severe outcomes from COVID-19

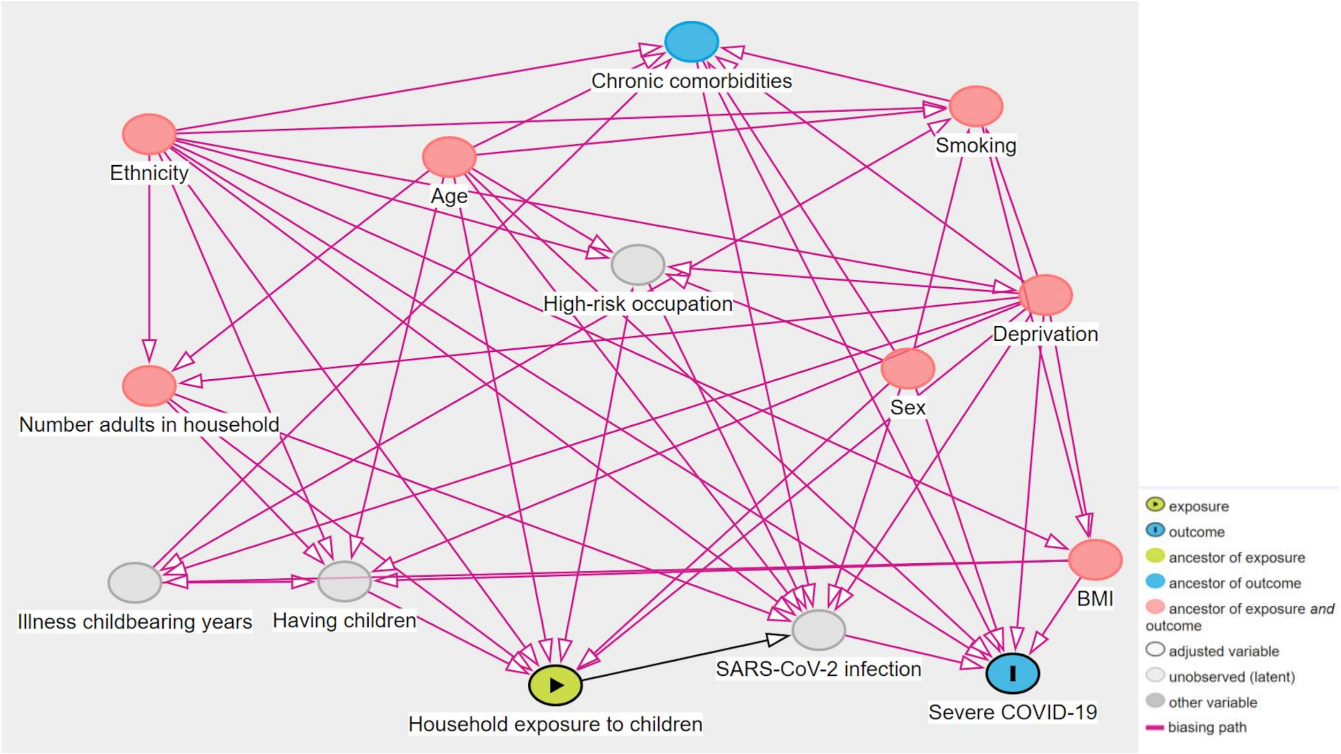

Explanatory note: Variables that could confound the relationship between household exposure to children and COVID-19 outcomes were identified using a directed acyclic graph. Both expert opinion and current literature were used to identify plausible confounders and to specify known causal paths. We can infer from the graph that we can estimate the total effect of household exposure to children on the risk of COVID-19 outcomes by adjusting for age, BMI, chronic comorbidities, deprivation, ethnicity, high-risk occupation, number adults in household and sex. Although we weren't able to adjust for high-risk occupation, we sought to consider the potential impact of this in the quantitative bias analysis.

Figure A2: Flow diagram of cohort with numbers excluded at different stages for wave 1 and wave 2

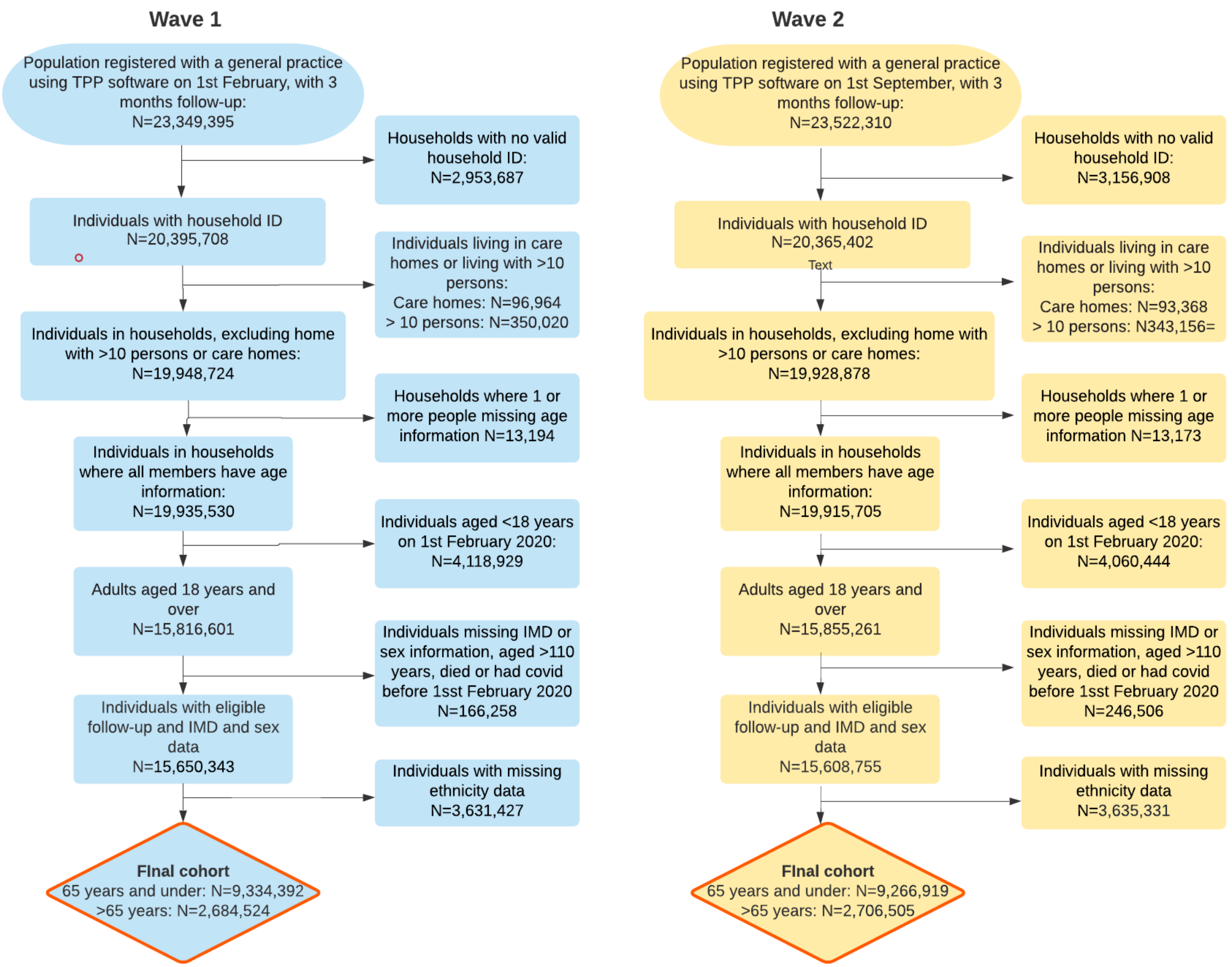

Figure A3: Histograms of frequencies of outcomes (recorded SARS-CoV-2 infection, COVID-19 outcomes and non-COVID-19 deaths) in wave 1 (1st February to 31st August 2020) and wave 2 (1st September to 18th December).

Wave 1

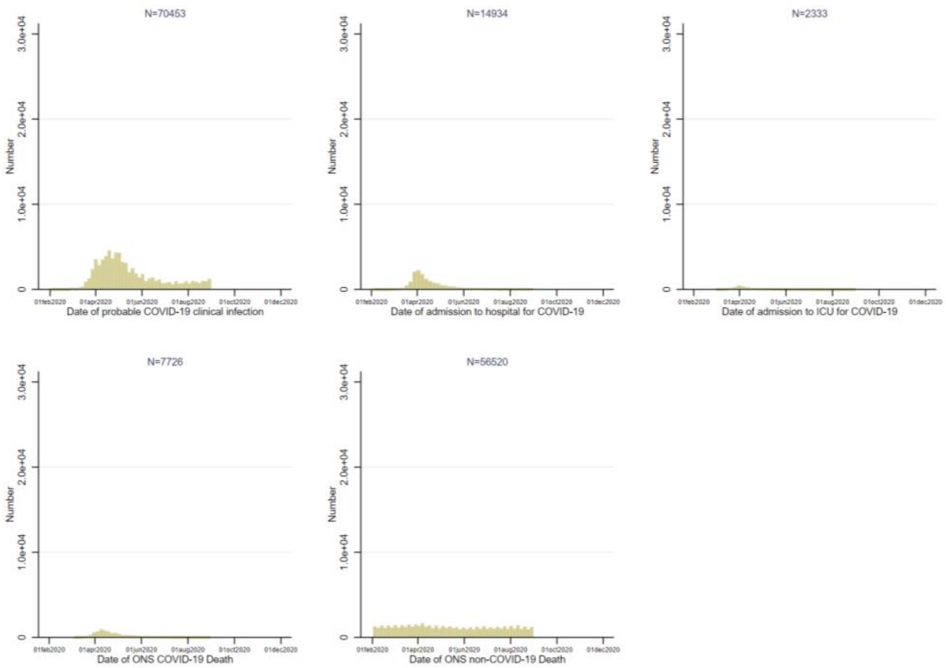

Wave 2

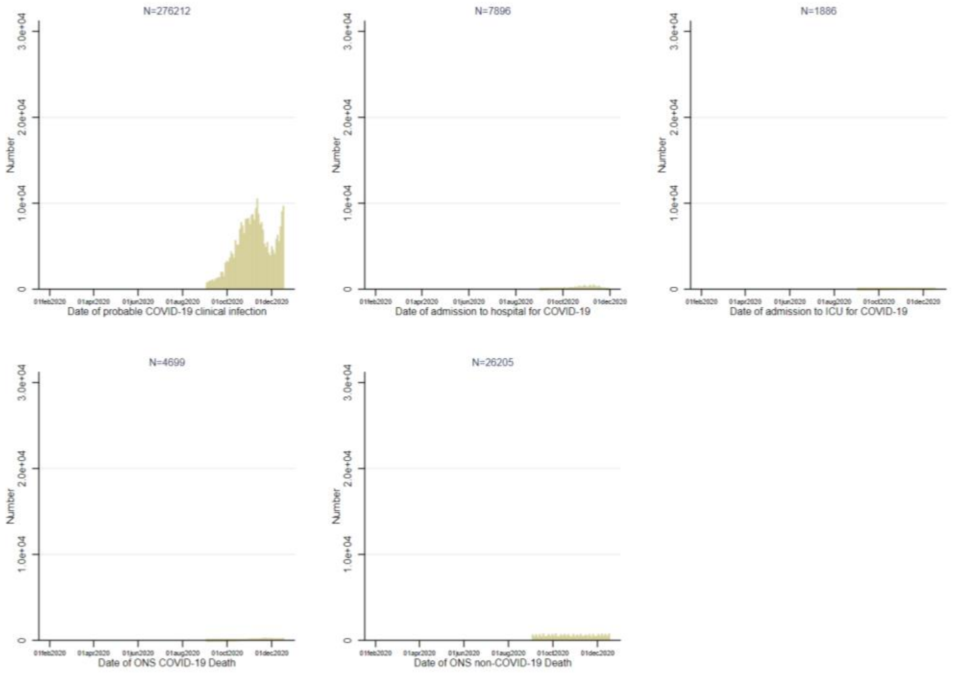

Figure A4: Adjusted* Hazard Ratios (HRs) for outcomes ((a) recorded SARS-CoV-2 infection, (b) COVID-19 hospital admission, (c) COVID-19 ICU admission, (d) COVID-19 death and (e) non-COVID-19 death) among adults aged >65 years, for waves 1 and 2 of the UK pandemic.

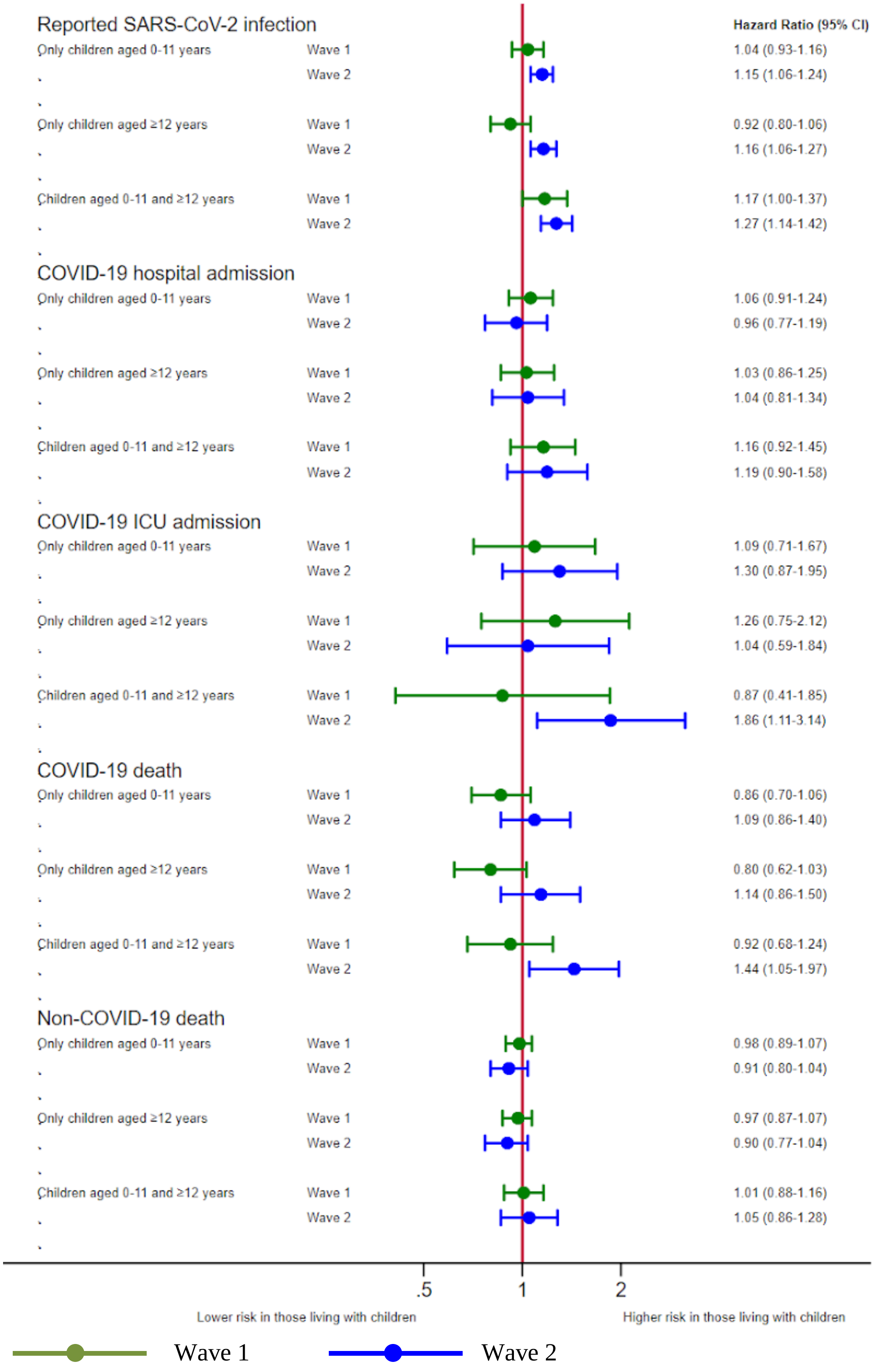

Figure A5. Comorbidity adjusted hazard ratios (HRs) and 95% confidence intervals (CI) for each COVID-19 outcome in wave 1 and wave 2, compared to having no children in the household by (a) sex, (b) shielding status among those >65 years.

a) Sex

Wave 1

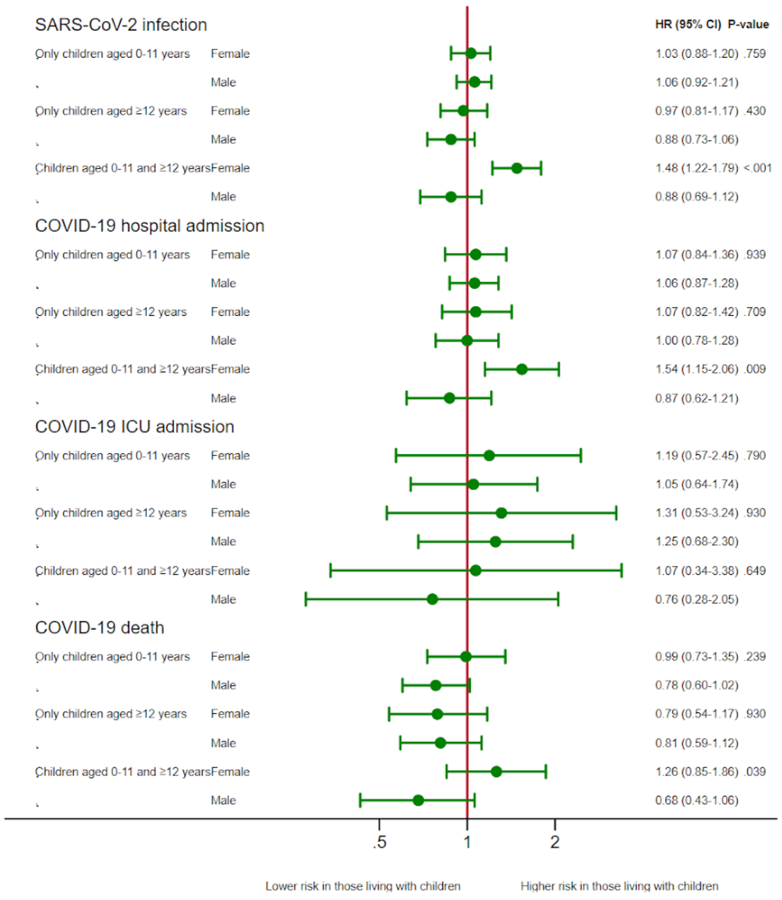

Wave 2

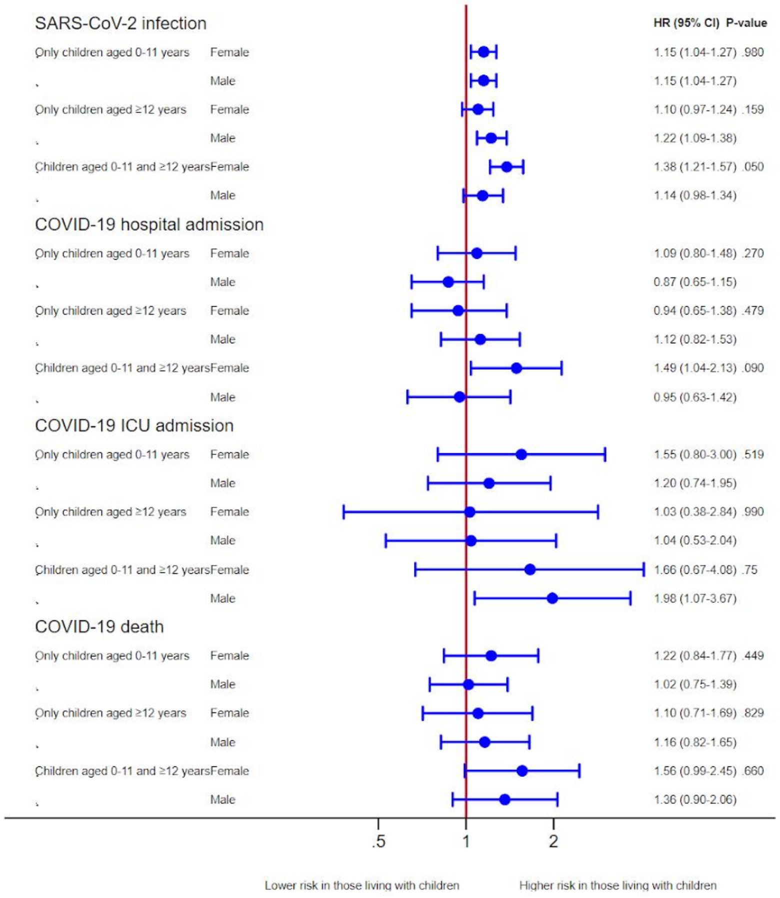

b) Shielding status

Wave 1

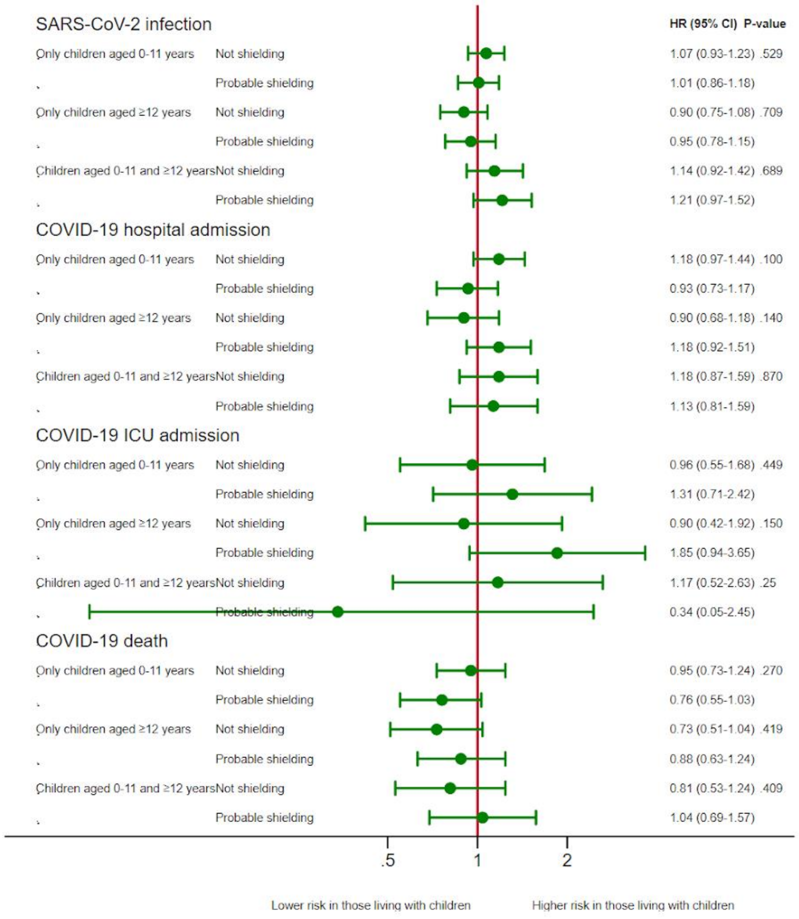

Wave 2

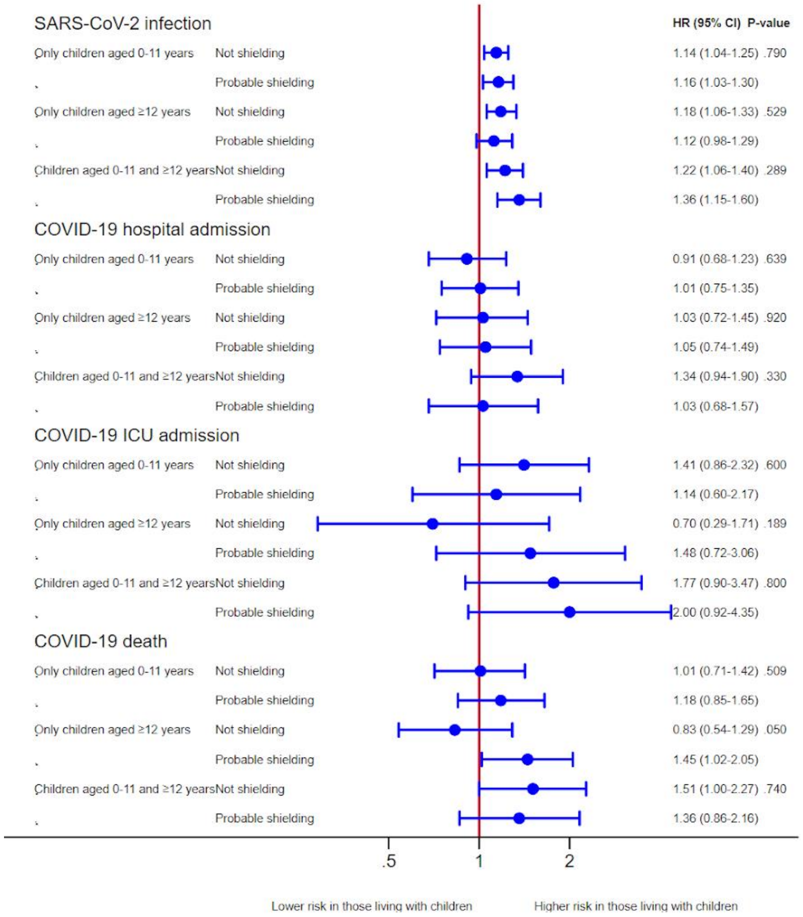

Figure A6: Results from sensitivity analysis: Hazard Ratios (HRs) for each COVID-19 outcome (a) evidence of SARS-CoV-2 infection recorded in primary care, (b) COVID-19 hospital admission, (c) COVID-19 ICU admission and (d) COVID-19 death), among those ≤65 years

(a) Recorded SARS-CoV-2 infection

Wave 1

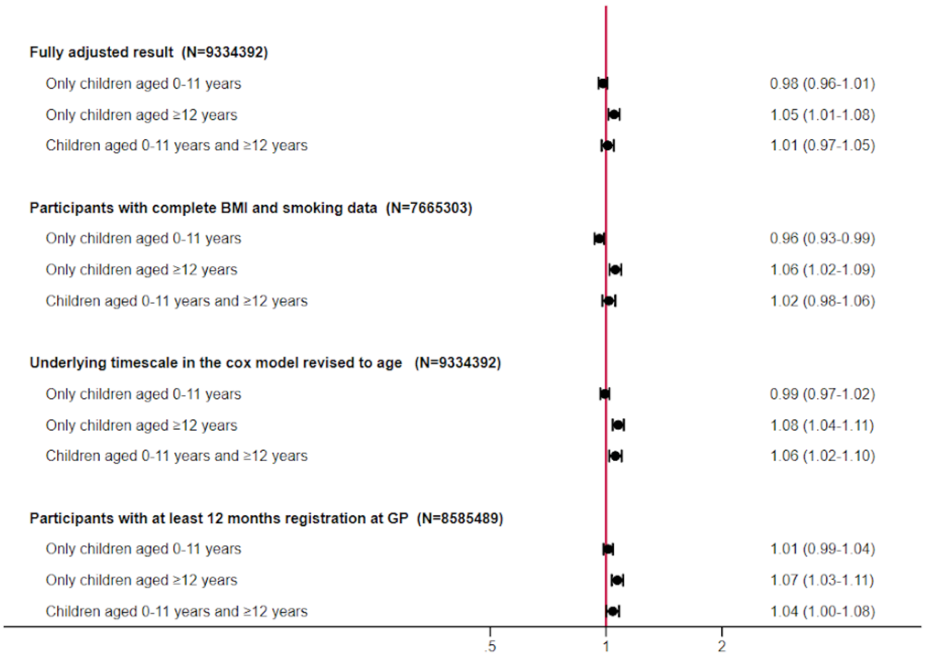

Wave 2

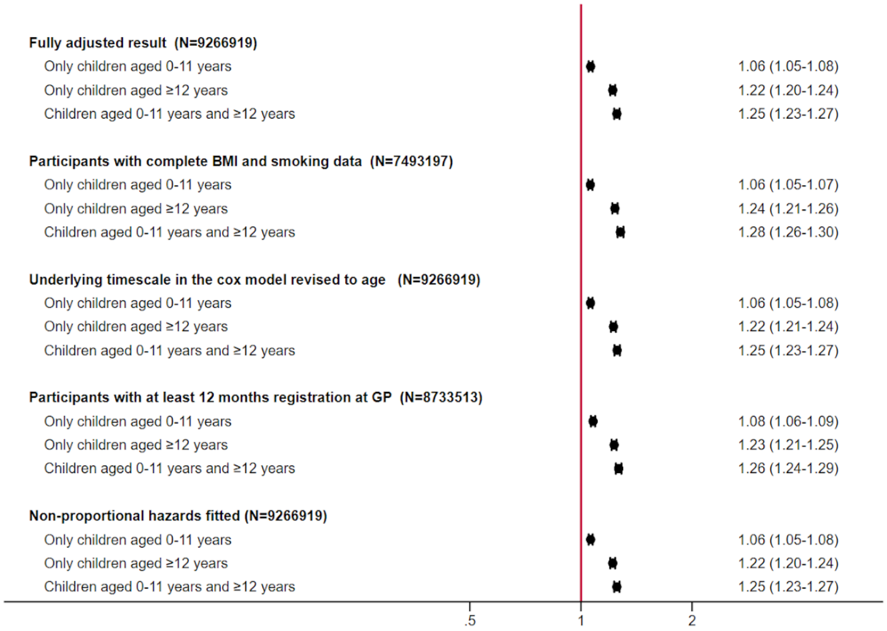

(b) COVID-19 hospital admission

Wave 1

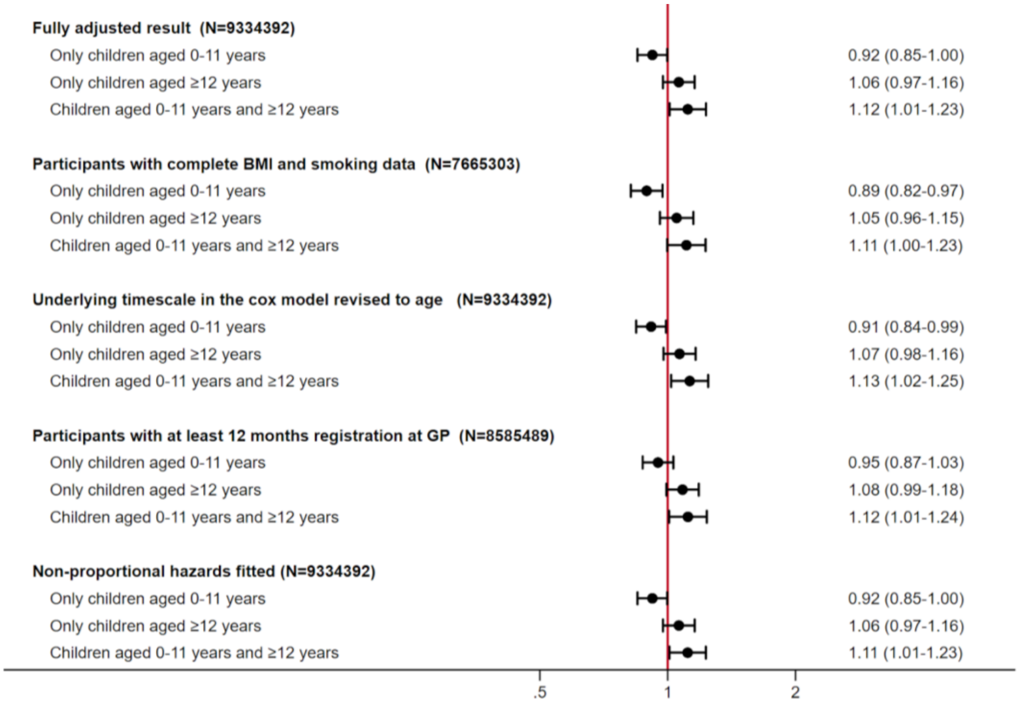

Wave 2

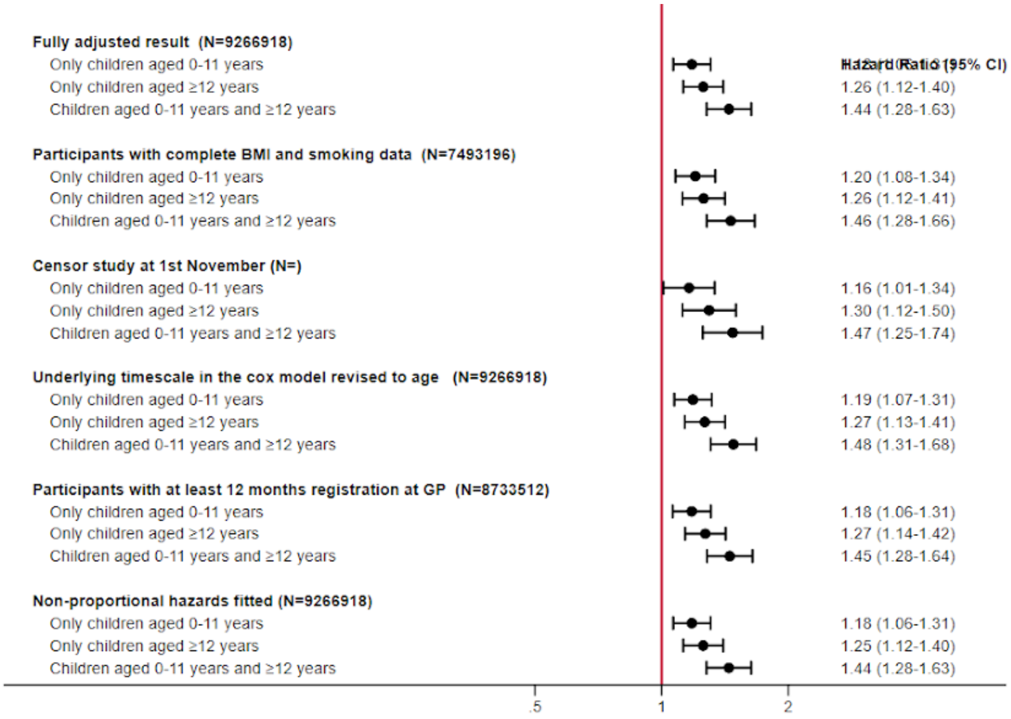

(c) COVID-19 ICU admission

Wave 1

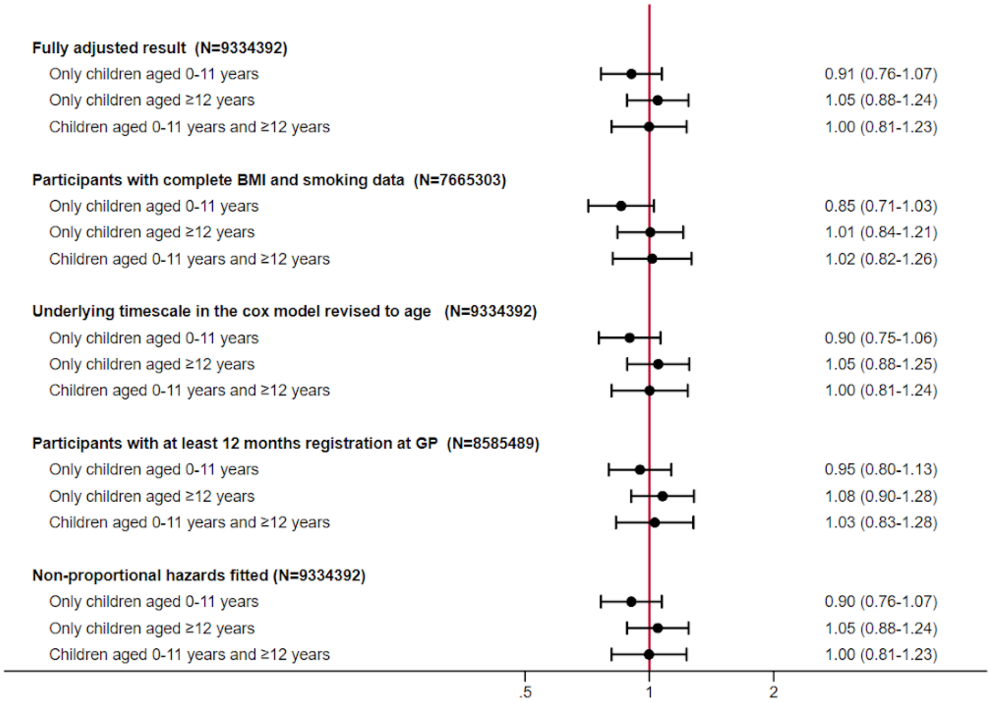

Wave 2

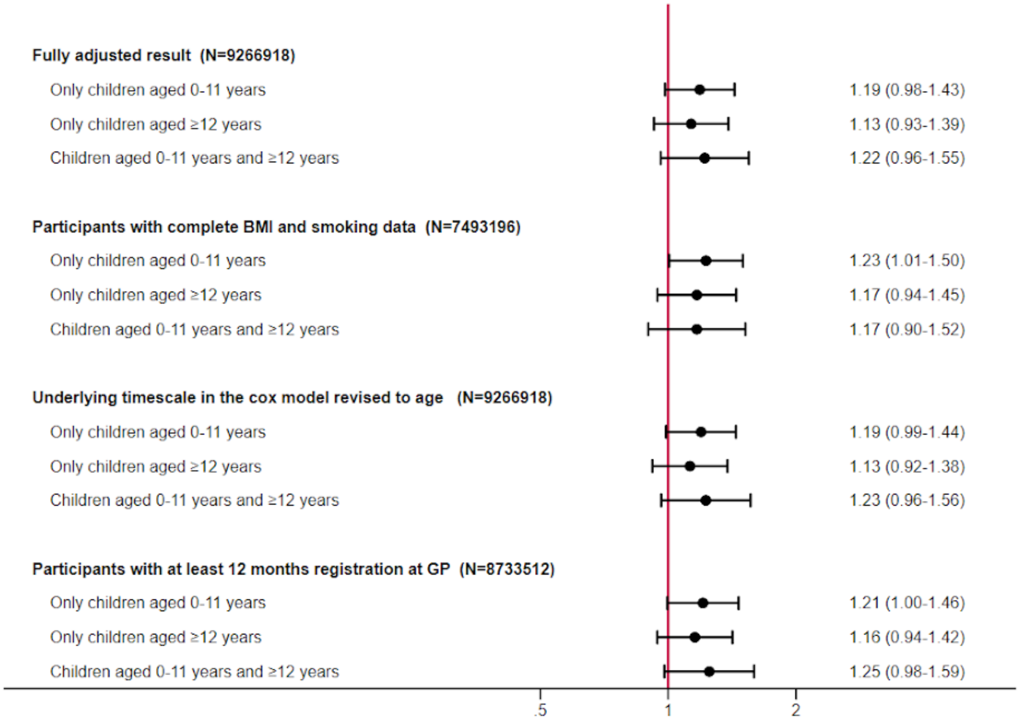

(d) COVID-19 death

Wave 1

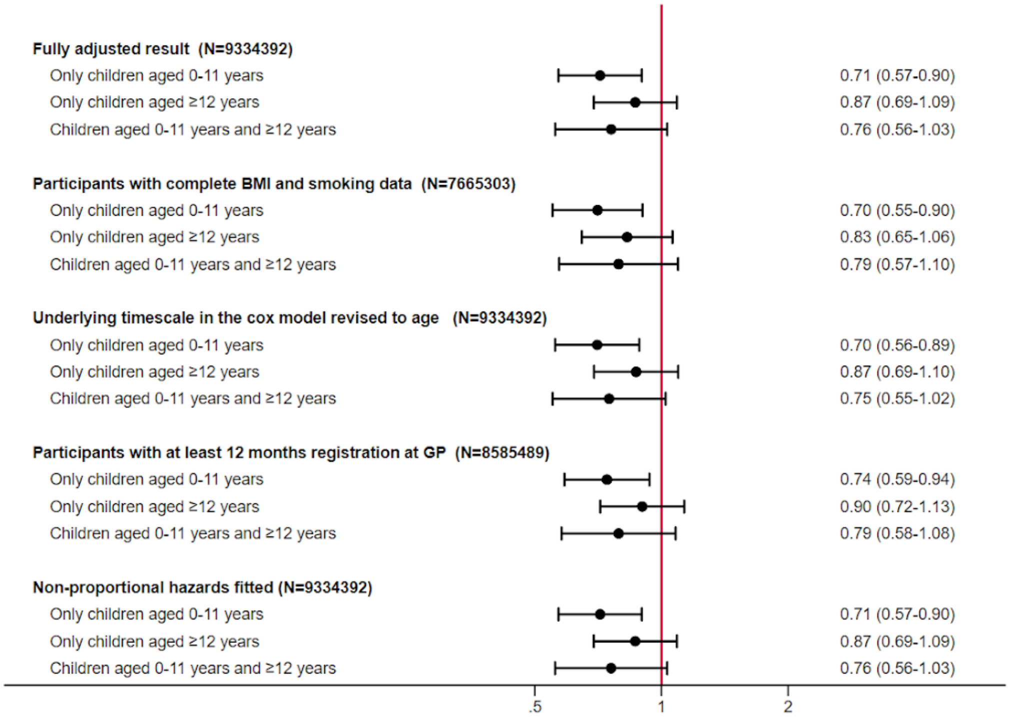

Wave 2

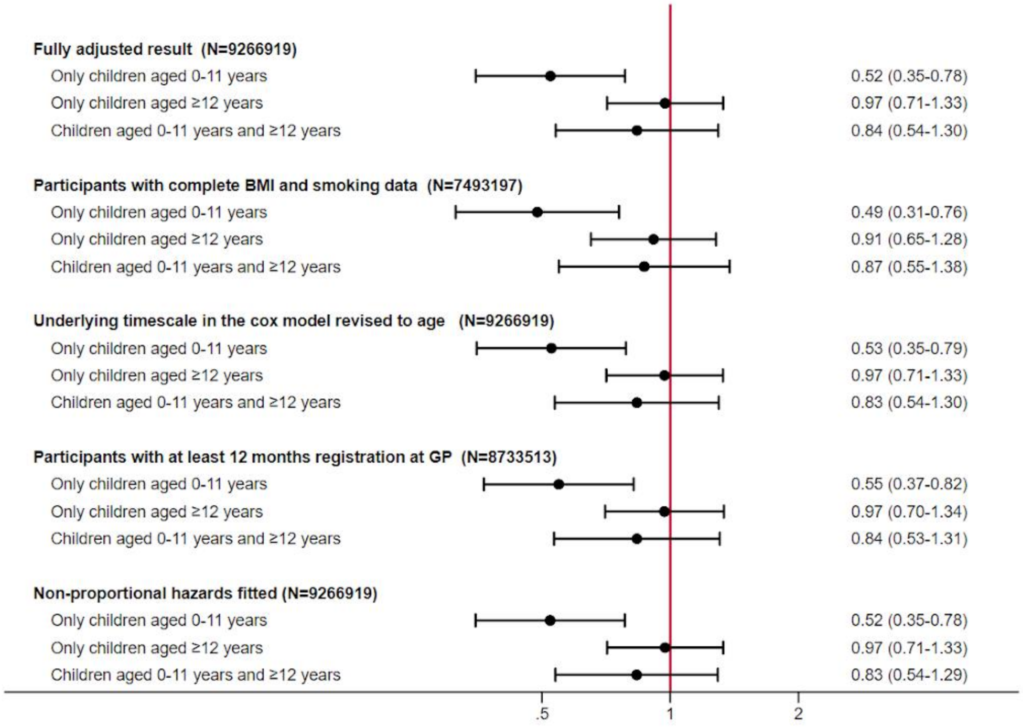

Figure A7: Results from sensitivity analysis creating alternative exposure definition which identifies children aged 0-4 years: Hazard Ratios (HRs) for each COVID-19 outcome (a) among those ≤65 years (b) among those >65 years

a) Age ≤65 years

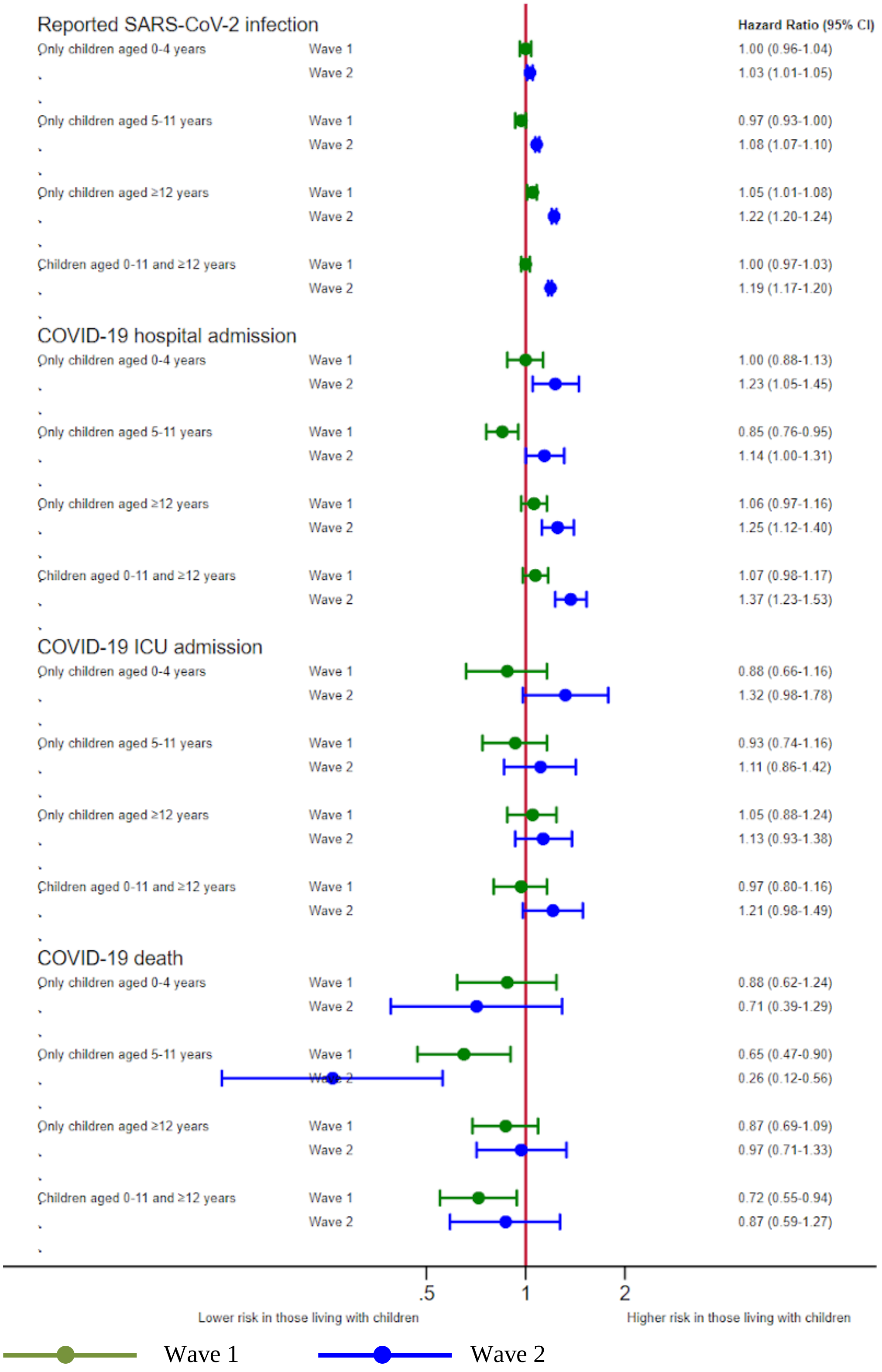

b) Age >65 years

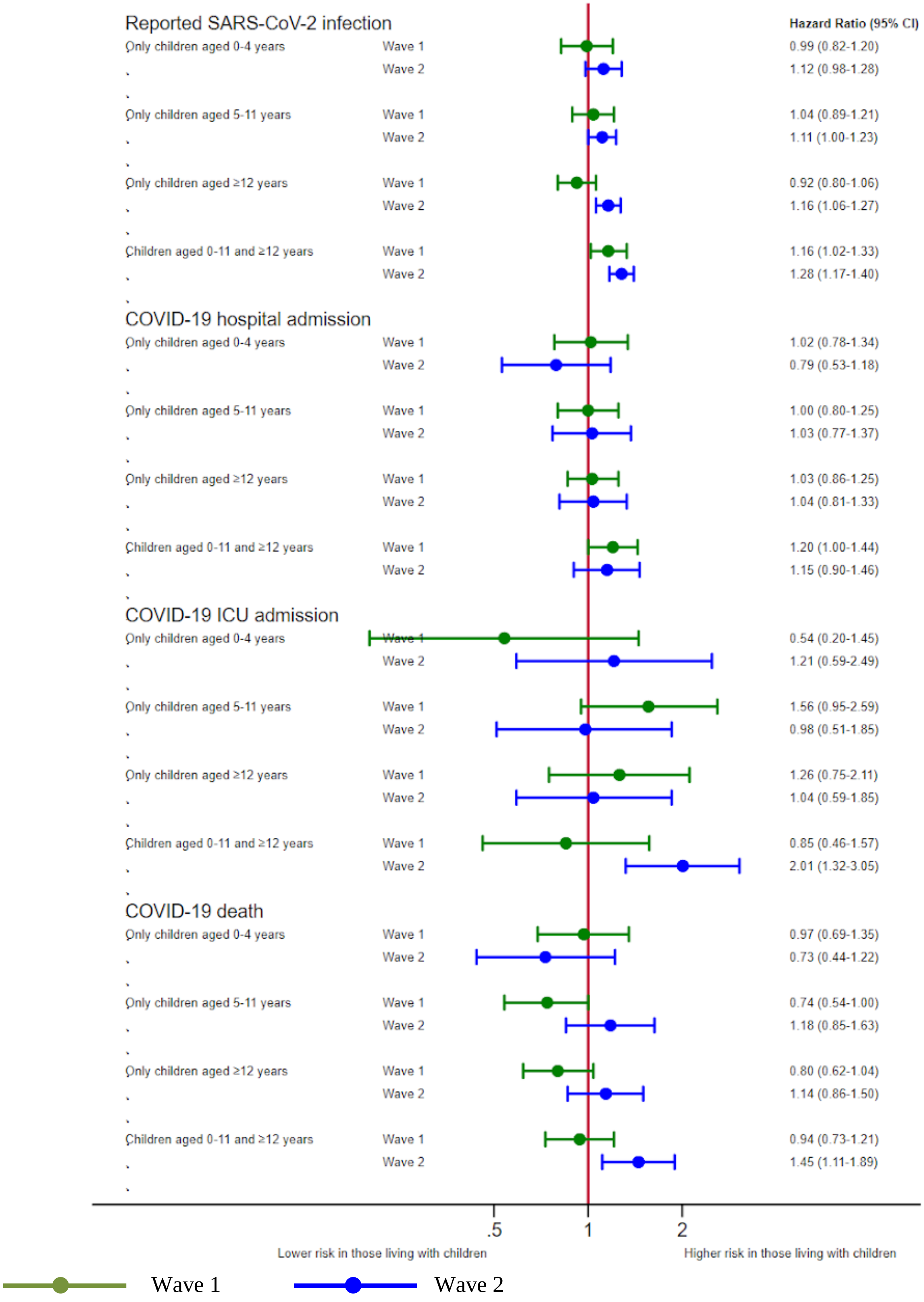

Tables

Table A1: Changes to the original study protocol

**Suggestion**	**Rationale**
Stratify all results on age above and below 65 years.	We were concerned that our age adjustment was not sufficient to capture the variation in risk of infection with SARS-CoV-2 among those working and after retirement.
Extract a second cohort to explore association in wave 2	We added an additional analysis to explore the association during wave 2 (September 1st to December 18th).
Revised exposure definition	We have revised the exposure group as follows: No children; only children 0-11 years; only children 12-18 years; both children 0-11 years and 12-18 years.
Revised SARS-CoV-2 definition	Given the marked increases in testing and improved data flows since study inception we have added SGSS test data in addition to primary care codes to define infection with SARS-CoV-2, increasing our ascertainment of infection. (SGSS is Public Health England's "Second Generation Surveillance System" in which SARS-CoV-2 PCR tests are recorded).
Add sensitivity analysis to assess risk of COVID-19 outcomes among adults living with nursery school aged children.	In light of current English (but not Scottish) Government policy around nurseries remaining open we included an additional analysis splitting the "only children 0 to 11 years" group into 0-4 (pre-school) and 5-11 years (primary school) aged children.
Separate COVID death and ICU admission	In early analyses the effect of living with children was in different directions for death from COVID-19 and ICU admission with COVID-19. In addition we had confirmed that there was adequate power to model each outcome separately.
Add non-COVID 19 death as an outcome	To contextualise our findings by comparing the risk of death from COVID-19 to and other causes.
Adjust for number of adults in household, rather than total number of people in the household	Data exploration showed that most of the additional people in a household were children, so results would have been hard to interpret if the 'dose-response' effect was also incorporated in the main model
Drop the planned sensitivity analysis using an alternative definition of COVID-19 in primary care to include diagnostic codes for suspected cases.	Exploration of suspected COVID-19 cases in primary care suggested there could be substantial misclassification of this outcome

Add outcome hospital admission for COVID-19	This outcome became available during the time course of the study, and it enabled us to examine whether the findings were consistent with those for ICU admission
Re-run analysis with age as the underlying timescale, as a sensitivity analysis	To confirm we are adequately adjusting for age.
Developing a directed-acyclic graph to inform our model covariates	To confirm we are adjusting for all appropriate variables and not introducing collider bias.
Additional model fitting a time-interaction with variables where there was evidence of non-proportional hazards	To examine whether introducing time interactions where there was evidence of non-proportional hazards materially altered our findings.
Use multiple imputation to impute missing data for ethnicity	To examine whether the reduced study population introduced by adjusting for ethnicity altered our results in a meaningful way.

Table A2. Cohort description, of adults >65 years, by presence of children in the household during Wave 1 and Wave 2 of the pandemic

	Wave 1 (1st February - 31st August)					Wave 2 (1st September - 18th December)				
		Children in the household (column %)					Children in the household (column %)			
	Total cohort, n (%)	No children	Only children aged 0-11 years	Only children aged 12 -18 years	Children aged 0-11 and children aged 12-18 years	Total cohort, n (%)	No children	Only children aged 0-11 years	Only children aged 12-18 years	Children aged 0-11 and children aged 12-18 years
**Total**	2684524 (100.0)	2595472 (100.00)	44420 (100.00)	29227 (100.00)	15405 (100.00)	2706505 (100.0)	2616423 (100.00)	44335 (100.00)	29854 (100.00)	15893 (100.00)
**Age**										
>65-70	633913 (23.6)	601603 (23.18)	17863 (40.21)	9030 (30.90)	5417 (35.16)	632027 (23.4)	599415 (22.91)	17826 (40.21)	9162 (30.69)	5624 (35.39)
70-80	1357522 (50.6)	1314301 (50.64)	20952 (47.17)	14752 (50.47)	7517 (48.80)	1375657 (50.8)	1331707 (50.90)	21017 (47.40)	15153 (50.76)	7780 (48.95)
80+	693089 (25.8)	679568 (26.18)	5605 (12.62)	5445 (18.63)	2471 (16.04)	698821 (25.8)	685301 (26.19)	5492 (12.39)	5539 (18.55)	2489 (15.66)
**Sex**										
Female	1445097 (53.8)	1397650 (53.85)	23411 (52.70)	15309 (52.38)	8727 (56.65)	1457578 (53.9)	1409510 (53.87)	23386 (52.75)	15668 (52.48)	9014 (56.72)
Male	1239427 (46.2)	1197822 (46.15)	21009 (47.30)	13918 (47.62)	6678 (43.35)	1248927 (46.1)	1206913 (46.13)	20949 (47.25)	14186 (47.52)	6879 (43.28)
**BMI (kg/m2)**										
<18.5	48518 (1.8)	47232 (1.82)	596 (1.34)	465 (1.59)	225 (1.46)	44591 (1.6)	43371 (1.66)	551 (1.24)	438 (1.47)	231 (1.45)
18.5-24.9	800412 (29.8)	777844 (29.97)	11709 (26.36)	7168 (24.53)	3691 (23.96)	793996 (29.3)	771543 (29.49)	11437 (25.80)	7215 (24.17)	3801 (23.92)
25-29.9	987076 (36.8)	955538 (36.82)	15690 (35.32)	10585 (36.22)	5263 (34.16)	994595 (36.7)	962848 (36.80)	15609 (35.21)	10703 (35.85)	5435 (34.20)
30-34.9 (Obese class I)	479258 (17.9)	461889 (17.80)	8560 (19.27)	5693 (19.48)	3116 (20.23)	485856 (18.0)	468212 (17.90)	8545 (19.27)	5891 (19.73)	3208 (20.18)
35-39.9 (Obese	156751 (5.8)	150696 (5.81)	2877 (6.48)	2073 (7.09)	1105 (7.17)	159518 (5.9)	153406 (5.86)	2893 (6.53)	2095 (7.02)	1124 (7.07)

class II)										
≥40 (Obese class III)	61135 (2.3)	58530 (2.26)	1227 (2.76)	875 (2.99)	503 (3.27)	62493 (2.3)	59863 (2.29)	1235 (2.79)	902 (3.02)	493 (3.10)
Missing	151374 (5.6)	143743 (5.54)	3761 (8.47)	2368 (8.10)	1502 (9.75)	165456 (6.1)	157180 (6.01)	4065 (9.17)	2610 (8.74)	1601 (10.07)
**Smoking**										
Never	1060734 (39.5)	1017749 (39.21)	21672 (48.79)	13150 (44.99)	8163 (52.99)	1073743 (39.7)	1030164 (39.37)	21685 (48.91)	13480 (45.15)	8414 (52.94)
Former	1395189 (52.0)	1359139 (52.37)	17673 (39.79)	12873 (44.04)	5504 (35.73)	1404161 (51.9)	1367878 (52.28)	17551 (39.59)	13095 (43.86)	5637 (35.47)
Current	222197 (8.3)	213161 (8.21)	4554 (10.25)	2974 (10.18)	1508 (9.79)	222251 (8.2)	213027 (8.14)	4593 (10.36)	3036 (10.17)	1595 (10.04)
Missing	6404 (0.2)	5423 (0.21)	521 (1.17)	230 (0.79)	230 (1.49)	6350 (0.2)	5354 (0.20)	506 (1.14)	243 (0.81)	247 (1.55)
**Ethnicity**										
White	2533944 (94.4)	2475149 (95.36)	28867 (64.99)	21381 (73.15)	8547 (55.48)	2552971 (94.3)	2493459 (95.30)	28815 (64.99)	21851 (73.19)	8846 (55.66)
Mixed	9970 (0.4)	8847 (0.34)	542 (1.22)	371 (1.27)	210 (1.36)	10249 (0.4)	9122 (0.35)	549 (1.24)	361 (1.21)	217 (1.37)
South Asian	91000 (3.4)	68550 (2.64)	11626 (26.17)	5525 (18.90)	5299 (34.40)	92732 (3.4)	70126 (2.68)	11582 (26.12)	5618 (18.82)	5406 (34.01)
Black	27818 (1.0)	24091 (0.93)	1761 (3.96)	1159 (3.97)	807 (5.24)	28209 (1.0)	24402 (0.93)	1771 (3.99)	1227 (4.11)	809 (5.09)
Other	21792 (0.8)	18835 (0.73)	1624 (3.66)	791 (2.71)	542 (3.52)	22344 (0.8)	19314 (0.74)	1618 (3.65)	797 (2.67)	615 (3.87)
**IMD quintile**										
1 (least deprived)	651069 (24.3)	635133 (24.47)	7829 (17.62)	5705 (19.52)	2402 (15.59)	668867 (24.7)	652542 (24.94)	7945 (17.92)	5868 (19.66)	2512 (15.81)
2	629037 (23.4)	612217 (23.59)	8482 (19.10)	5656 (19.35)	2682 (17.41)	636250 (23.5)	619228 (23.67)	8386 (18.92)	5888 (19.72)	2748 (17.29)
3	580534 (21.6)	561800 (21.65)	9483 (21.35)	6272 (21.46)	2979 (19.34)	574847 (21.2)	556103 (21.25)	9309 (21.00)	6331 (21.21)	3104 (19.53)
4	477447 (17.8)	457663 (17.63)	9876 (22.23)	6212 (21.25)	3696 (23.99)	479967 (17.7)	459987 (17.58)	9980 (22.51)	6246 (20.92)	3754 (23.62)
5 (most deprived)	346437 (12.9)	328659 (12.66)	8750 (19.70)	5382 (18.41)	3646 (23.67)	346574 (12.8)	328563 (12.56)	8715 (19.66)	5521 (18.49)	3775 (23.75)
**Total number adults in household**										

1	913998 (34.0)	910750 (35.09)	1435 (3.23)	1549 (5.30)	264 (1.71)	921594 (34.1)	918322 (35.10)	1432 (3.23)	1579 (5.29)	261 (1.64)
2	1384390 (51.6)	1367469 (52.69)	7590 (17.09)	7295 (24.96)	2036 (13.22)	1390726 (51.4)	1373830 (52.51)	7533 (16.99)	7296 (24.44)	2067 (13.01)
≥3	386136 (14.4)	317253 (12.22)	35395 (79.68)	20383 (69.74)	13105 (85.07)	394185 (14.6)	324271 (12.39)	35370 (79.78)	20979 (70.27)	13565 (85.35)
**Blood pressure**										
Normal	309796 (11.5)	298880 (11.52)	5551 (12.50)	3457 (11.83)	1908 (12.39)	310503 (11.5)	299723 (11.46)	5394 (12.17)	3510 (11.76)	1876 (11.80)
Elevated	417855 (15.6)	404259 (15.58)	6827 (15.37)	4429 (15.15)	2340 (15.19)	422947 (15.6)	409265 (15.64)	6749 (15.22)	4496 (15.06)	2437 (15.33)
High Stage 1	990139 (36.9)	957771 (36.90)	16184 (36.43)	10608 (36.30)	5576 (36.20)	1011992 (37.4)	978709 (37.41)	16518 (37.26)	10989 (36.81)	5776 (36.34)
High Stage 2	952842 (35.5)	923056 (35.56)	14516 (32.68)	10192 (34.87)	5078 (32.96)	946777 (35.0)	916919 (35.04)	14329 (32.32)	10281 (34.44)	5248 (33.02)
Missing	13892 (0.5)	11506 (0.44)	1342 (3.02)	541 (1.85)	503 (3.27)	14286 (0.5)	11807 (0.45)	1345 (3.03)	578 (1.94)	556 (3.50)
**High bp or diagnosed hypertension**	1859250 (69.3)	1799565 (69.33)	29235 (65.81)	20126 (68.86)	10324 (67.02)	1858019 (68.7)	1798291 (68.73)	28917 (65.22)	20336 (68.12)	10475 (65.91)
Comorbidities										
**Chronic respiratory disease ex asthma**	334940 (12.5)	324968 (12.52)	4769 (10.74)	3627 (12.41)	1576 (10.23)	324691 (12.0)	315049 (12.04)	4507 (10.17)	3563 (11.93)	1572 (9.89)
**Asthma**	362235 (13.5)	349399 (13.46)	6392 (14.39)	4190 (14.34)	2254 (14.63)	366433 (13.5)	353571 (13.51)	6353 (14.33)	4202 (14.08)	2307 (14.52)
**Chronic cardiac disease**	571371 (21.3)	553202 (21.31)	8687 (19.56)	6283 (21.50)	3199 (20.77)	551840 (20.4)	534326 (20.42)	8245 (18.60)	6174 (20.68)	3095 (19.47)
**Diabetes**										
No diabetes	2201205 (82.0)	2135805 (82.29)	32686 (73.58)	21947 (75.09)	10767 (69.89)	2221833 (82.1)	2155520 (82.38)	32726 (73.82)	22454 (75.21)	11133 (70.05)
Type 1, controlled	3563 (0.1)	3463 (0.13)	48 (0.11)	36 (0.12)	16 (0.10)	3710 (0.1)	3609 (0.14)	48 (0.11)	37 (0.12)	16 (0.10)
Type 1, uncontrolled	7408 (0.3)	7191 (0.28)	98 (0.22)	80 (0.27)	39 (0.25)	7352 (0.3)	7137 (0.27)	104 (0.23)	74 (0.25)	37 (0.23)
Type 2, controlled	323854 (12.1)	309255 (11.92)	7246 (16.31)	4578 (15.66)	2775 (18.01)	323947 (12.0)	309264 (11.82)	7154 (16.14)	4691 (15.71)	2838 (17.86)
Type 2,	146537 (5.5)	137999 (5.32)	4251 (9.57)	2530 (8.66)	1757 (11.41)	147893 (5.5)	139316 (5.32)	4196 (9.46)	2552 (8.55)	1829 (11.51)

uncontrolled										
Diabetes, no HbA1c	1957 (0.1)	1759 (0.07)	91 (0.20)	56 (0.19)	51 (0.33)	1770 (0.1)	1577 (0.06)	107 (0.24)	46 (0.15)	40 (0.25)
**Haematological cancer**										
Diagnosed < 1 year ago	3839 (0.1)	3735 (0.14)	48 (0.11)	39 (0.13)	17 (0.11)	3499 (0.1)	3410 (0.13)	36 (0.08)	35 (0.12)	18 (0.11)
Diagnosed 1-4.9 years ago	11638 (0.4)	11326 (0.44)	149 (0.34)	113 (0.39)	50 (0.32)	11138 (0.4)	10848 (0.41)	134 (0.30)	113 (0.38)	43 (0.27)
Diagnosed ≥5 years ago	23786 (0.9)	23176 (0.89)	295 (0.66)	205 (0.70)	110 (0.71)	23008 (0.9)	22415 (0.86)	286 (0.65)	202 (0.68)	105 (0.66)
**Non-haematological cancer**										
Diagnosed < 1 year ago	32334 (1.2)	31478 (1.21)	447 (1.01)	282 (0.96)	127 (0.82)	29009 (1.1)	28240 (1.08)	399 (0.90)	249 (0.83)	121 (0.76)
Diagnosed 1-4.9 years ago	95696 (3.6)	93208 (3.59)	1211 (2.73)	850 (2.91)	427 (2.77)	92748 (3.4)	90331 (3.45)	1191 (2.69)	819 (2.74)	407 (2.56)
Diagnosed ≥5 years ago	254667 (9.5)	249106 (9.60)	2670 (6.01)	2066 (7.07)	825 (5.36)	248878 (9.2)	243450 (9.30)	2565 (5.79)	2060 (6.90)	803 (5.05)
**Reduced kidney function**										
Estimated GFR 30-60	563148 (21.0)	548113 (21.12)	6880 (15.49)	5454 (18.66)	2701 (17.53)	560736 (20.7)	545878 (20.86)	6720 (15.16)	5474 (18.34)	2664 (16.76)
Estimated GFR <30	41148 (1.5)	39702 (1.53)	668 (1.50)	485 (1.66)	293 (1.90)	40212 (1.5)	38848 (1.48)	612 (1.38)	471 (1.58)	281 (1.77)
**End-stage renal disease***	6885 (0.3)	6498 (0.25)	183 (0.41)	117 (0.40)	87 (0.56)	6285 (0.2)	5934 (0.23)	159 (0.36)	110 (0.37)	82 (0.52)
**Chronic Liver disease**	26389 (1.0)	25427 (0.98)	456 (1.03)	316 (1.08)	190 (1.23)	25834 (1.0)	24900 (0.95)	426 (0.96)	319 (1.07)	189 (1.19)
**Stroke/dementia**	204093 (7.6)	198024 (7.63)	2868 (6.46)	2095 (7.17)	1106 (7.18)	191057 (7.1)	185318 (7.08)	2676 (6.04)	2016 (6.75)	1047 (6.59)

**Other neurological disease**	54121 (2.0)	52516 (2.02)	775 (1.74)	528 (1.81)	302 (1.96)	51397 (1.9)	49856 (1.91)	719 (1.62)	534 (1.79)	288 (1.81)
**Solid organ transplant****	872 (0.0)	821 (0.03)	22 (0.05)	17 (0.06)	12 (0.08)	852 (0.0)	802 (0.03)	19 (0.04)	17 (0.06)	14 (0.09)
**Asplenia**	6733 (0.3)	6556 (0.25)	79 (0.18)	70 (0.24)	28 (0.18)	6639 (0.2)	6458 (0.25)	88 (0.20)	67 (0.22)	26 (0.16)
**Rheumatoid/Lupus / Psoriasis**	208673 (7.8)	202704 (7.81)	2951 (6.64)	2096 (7.17)	922 (5.99)	208797 (7.7)	202856 (7.75)	2917 (6.58)	2061 (6.90)	963 (6.06)
**Other immunosuppressive condition**	5397 (0.2)	5192 (0.20)	99 (0.22)	70 (0.24)	36 (0.23)	5658 (0.2)	5448 (0.21)	110 (0.25)	68 (0.23)	32 (0.20)
**Probable shielding*****	956589 (35.6)	928407 (35.77)	13753 (30.96)	9756 (33.38)	4673 (30.33)	941453 (34.8)	913752 (34.92)	13374 (30.17)	9663 (32.37)	4664 (29.35)
**Any comorbidity******	1638898 (61.0)	1584706 (61.06)	26541 (59.75)	18171 (62.17)	9480 (61.54)	1623911 (60.0)	1570025 (60.01)	26049 (58.75)	18226 (61.05)	9611 (60.47)

*End-stage renal disease includes on dialysis or having had a kidney transplant
**All solid organ transplants, excluding kidney
***Shielding includes organ transplant recipients, renal replacement therapy, haematological cancers, non-haematological cancers, immunodeficiencies/asplenia and severe respiratory conditions.
****Any comorbidity includes: chronic respiratory disease, asthma, chronic cardiac disease, diabetes, cancer, end-stage renal disease, chronic liver disease, stroke or dementia, other neurological disease, other transplant, asplenia, Rheumatoid/Lupus/ Psoriasis or other immunosuppressive condition

Table A3. Cohort description by outcomes (evidence of SARS-CoV-2 infection recorded in primary care, COVID-19 outcomes and non-COVID 19 deaths) for adults ≤65 years in wave 1 and wave 2 of the UK pandemic

	Wave 1 (1st February to 31st August 2020)						Wave 2 (1st February to 31st August 2020)					
		Number (%) within stratum)						Number (%) within stratum)				
	N (column %)	Recorded SARS-CoV-2 infection	COVID-19 hospital admissions	COVID-19 ICU admissions	COVID-19 deaths	Non-COVID-19 deaths	N (column %)	Recorded SARS-CoV-2 infection	COVID-19 hospital admissions	COVID-19 ICU admissions	COVID-19 deaths	Non-COVID-19 deaths
**Total**	9334392 (100.0)	51560 (0.55)	6374 (0.07)	1601 (0.02)	1219 (0.01)	10580 (0.11)	9266919 (100.0)	241693 (2.61)	3616 (0.04)	1102 (0.01)	591 (0.01)	4409 (0.05)
**Age**												
18-<30	1981860 (21.2)	9778 (0.49)	345 (0.02)	45 (0.00)	12 (0.00)	280 (0.01)	1912652 (20.6)	62523 (3.27)	246 (0.01)	39 (0.00)	6 (0.00)	60 (0.00)
30-<40	2230655 (23.9)	11580 (0.52)	766 (0.03)	132 (0.01)	46 (0.00)	654 (0.03)	2222046 (24.0)	56007 (2.52)	440 (0.02)	102 (0.00)	14 (0.00)	205 (0.01)
40-<50	2022059 (21.7)	11897 (0.59)	1402 (0.07)	316 (0.02)	160 (0.01)	1671 (0.08)	2021030 (21.8)	52553 (2.60)	771 (0.04)	222 (0.01)	67 (0.00)	647 (0.03)
50-<60	2062371 (22.1)	12740 (0.62)	2344 (0.11)	648 (0.03)	466 (0.02)	4001 (0.19)	2064172 (22.3)	51315 (2.49)	1302 (0.06)	426 (0.02)	250 (0.01)	1772 (0.09)
60-<66	1037447 (11.1)	5565 (0.54)	1517 (0.15)	460 (0.04)	535 (0.05)	3974 (0.38)	1047019 (11.3)	19295 (1.84)	857 (0.08)	313 (0.03)	254 (0.02)	1725 (0.16)
**Sex**												
Female	4802386 (51.4)	30629 (0.64)	2740 (0.06)	513 (0.01)	436 (0.01)	4284 (0.09)	4757515 (51.3)	135947 (2.86)	1573 (0.03)	342 (0.01)	201 (0.00)	1866 (0.04)
Male	4532006 (48.6)	20931 (0.46)	3634 (0.08)	1088 (0.02)	783 (0.02)	6296 (0.14)	4509404 (48.7)	105746 (2.35)	2043 (0.05)	760 (0.02)	390 (0.01)	2543 (0.06)
**BMI (kg/m2)**												
<18.5	180395 (1.9)	804 (0.45)	74 (0.04)	9 (0.00)	22 (0.01)	579 (0.32)	177343 (1.9)	3515 (1.98)	31 (0.02)	5 (0.00)	11 (0.01)	230 (0.13)
18.5-24.9	2886762 (30.9)	13498 (0.47)	981 (0.03)	184 (0.01)	194 (0.01)	3170 (0.11)	2822640 (30.5)	66149 (2.34)	442 (0.02)	97 (0.00)	74 (0.00)	1286 (0.05)
25-29.9	2551873 (27.3)	14743 (0.58)	1829 (0.07)	434 (0.02)	319 (0.01)	2714 (0.11)	2489283 (26.9)	67124 (2.70)	944 (0.04)	248 (0.01)	131 (0.01)	1107 (0.04)
30-34.9 (Obese class I)	1286837 (13.8)	9118 (0.71)	1470 (0.11)	414 (0.03)	251 (0.02)	1610 (0.13)	1256346 (13.6)	38047 (3.03)	890 (0.07)	314 (0.02)	154 (0.01)	710 (0.06)
35-39.9 (Obese class II)	521821 (5.6)	4207 (0.81)	824 (0.16)	232 (0.04)	180 (0.03)	737 (0.14)	510314 (5.5)	16402 (3.21)	518 (0.10)	169 (0.03)	87 (0.02)	339 (0.07)
≥40 (Obese class III)	281814 (3.0)	2655 (0.94)	601 (0.21)	170 (0.06)	123 (0.04)	584 (0.21)	275736 (3.0)	9298 (3.37)	397 (0.14)	142 (0.05)	71 (0.03)	233 (0.08)
Missing	1624890 (17.4)	6535 (0.40)	595 (0.04)	158 (0.01)	130 (0.01)	1186 (0.07)	1735257 (18.7)	41158 (2.37)	394 (0.02)	127 (0.01)	63 (0.00)	504 (0.03)

**Smoking**												
Never	4491312 (48.1)	28132 (0.63)	3342 (0.07)	814 (0.02)	509 (0.01)	2795 (0.06)	4451215 (48.0)	129583 (2.91)	1840 (0.04)	524 (0.01)	214 (0.00)	1199 (0.03)
Former	2684880 (28.8)	15829 (0.59)	2389 (0.09)	669 (0.02)	515 (0.02)	3652 (0.14)	2677563 (28.9)	73998 (2.76)	1468 (0.05)	500 (0.02)	292 (0.01)	1568 (0.06)
Current	1900976 (20.4)	6683 (0.35)	602 (0.03)	111 (0.01)	190 (0.01)	4073 (0.21)	1866970 (20.1)	30547 (1.64)	287 (0.02)	69 (0.00)	85 (0.00)	1613 (0.09)
Missing	257224 (2.8)	916 (0.36)	41 (0.02)	7 (0.00)	REDACTED	60 (0.02)	271171 (2.9)	7565 (2.79)	21 (0.01)	9 (0.00)	0 (0.00)	29 (0.01)
**Ethnicity**												
White	7734228 (82.9)	35276 (0.46)	4120 (0.05)	1002 (0.01)	827 (0.01)	9604 (0.12)	7666700 (82.7)	190672 (2.49)	2470 (0.03)	731 (0.01)	471 (0.01)	4002 (0.05)
Mixed	160065 (1.7)	1052 (0.66)	161 (0.10)	46 (0.03)	28 (0.02)	95 (0.06)	160932 (1.7)	4026 (2.50)	66 (0.04)	24 (0.01)	6 (0.00)	43 (0.03)
South Asian	836666 (9.0)	11067 (1.32)	1291 (0.15)	328 (0.04)	220 (0.03)	484 (0.06)	835735 (9.0)	34963 (4.18)	789 (0.09)	262 (0.03)	93 (0.01)	203 (0.02)
Black	309079 (3.3)	2656 (0.86)	525 (0.17)	148 (0.05)	103 (0.03)	281 (0.09)	309730 (3.3)	7029 (2.27)	184 (0.06)	52 (0.02)	17 (0.01)	110 (0.04)
Other	294354 (3.2)	1509 (0.51)	277 (0.09)	77 (0.03)	41 (0.01)	116 (0.04)	293822 (3.2)	5003 (1.70)	107 (0.04)	33 (0.01)	REDACTED	51 (0.02)
**IMD quintile**												
1 (least deprived)	1724481 (18.5)	7391 (0.43)	854 (0.05)	197 (0.01)	121 (0.01)	1343 (0.08)	1741677 (18.8)	41659 (2.39)	437 (0.03)	123 (0.01)	52 (0.00)	602 (0.03)
2	1850024 (19.8)	8627 (0.47)	1021 (0.06)	260 (0.01)	177 (0.01)	1666 (0.09)	1837273 (19.8)	43514 (2.37)	494 (0.03)	155 (0.01)	72 (0.00)	734 (0.04)
3	1942791 (20.8)	9870 (0.51)	1161 (0.06)	313 (0.02)	227 (0.01)	1975 (0.10)	1898184 (20.5)	45357 (2.39)	574 (0.03)	179 (0.01)	86 (0.00)	830 (0.04)
4	2010621 (21.5)	12239 (0.61)	1577 (0.08)	406 (0.02)	351 (0.02)	2486 (0.12)	1994633 (21.5)	54108 (2.71)	832 (0.04)	252 (0.01)	141 (0.01)	1026 (0.05)
5 (most deprived)	1806475 (19.4)	13433 (0.74)	1761 (0.10)	425 (0.02)	343 (0.02)	3110 (0.17)	1795152 (19.4)	57055 (3.18)	1279 (0.07)	393 (0.02)	240 (0.01)	1217 (0.07)
**Total number adults in household**												
1	2260091 (24.2)	10670 (0.47)	1459 (0.06)	346 (0.02)	339 (0.01)	3912 (0.17)	2223814 (24.0)	49780 (2.24)	775 (0.03)	209 (0.01)	166 (0.01)	1497 (0.07)
2	3850961 (41.3)	20288 (0.53)	2319 (0.06)	564 (0.01)	392 (0.01)	3836 (0.10)	3772870 (40.7)	94536 (2.51)	1280 (0.03)	399 (0.01)	201 (0.01)	1686 (0.04)
≥3	3223340 (34.5)	20602 (0.64)	2596 (0.08)	691 (0.02)	488 (0.02)	2832 (0.09)	3270235 (35.3)	97377 (2.98)	1561 (0.05)	494 (0.02)	224 (0.01)	1226 (0.04)
**Blood pressure**												
Normal	2475941 (26.5)	13741 (0.55)	1232 (0.05)	218 (0.01)	235 (0.01)	2506 (0.10)	2452608 (26.5)	68001 (2.77)	663 (0.03)	142 (0.01)	84 (0.00)	1020 (0.04)
Elevated	1347313 (14.4)	7493 (0.56)	777 (0.06)	183 (0.01)	145 (0.01)	1371 (0.10)	1335410 (14.4)	36135 (2.71)	526 (0.04)	143 (0.01)	85 (0.01)	557 (0.04)
High Stage 1	2935415 (31.4)	17396 (0.59)	2418 (0.08)	642 (0.02)	449 (0.02)	3403 (0.12)	2924903 (31.6)	77834 (2.66)	1382 (0.05)	443 (0.02)	227 (0.01)	1489 (0.05)

High Stage 2	1585467 (17.0)	9740 (0.61)	1787 (0.11)	517 (0.03)	374 (0.02)	3075 (0.19)	1552001 (16.7)	38762 (2.50)	952 (0.06)	352 (0.02)	190 (0.01)	1267 (0.08)
Missing	990256 (10.6)	3190 (0.32)	160 (0.02)	41 (0.00)	16 (0.00)	225 (0.02)	1001997 (10.8)	20961 (2.09)	93 (0.01)	22 (0.00)	REDACTED	76 (0.01)
**High bp or diagnosed hypertension**	2221567 (23.8)	14915 (0.67)	3087 (0.14)	908 (0.04)	735 (0.03)	5194 (0.23)	2174941 (23.5)	55350 (2.54)	1634 (0.08)	611 (0.03)	341 (0.02)	2216 (0.10)
Comorbidities												
**Chronic respiratory disease ex asthma**	196442 (2.1)	1706 (0.87)	602 (0.31)	152 (0.08)	177 (0.09)	1821 (0.93)	185356 (2.0)	4401 (2.37)	412 (0.22)	145 (0.08)	114 (0.06)	766 (0.41)
**Asthma**	1607197 (17.2)	9659 (0.60)	1430 (0.09)	325 (0.02)	243 (0.02)	1974 (0.12)	1595772 (17.2)	45694 (2.86)	906 (0.06)	249 (0.02)	130 (0.01)	792 (0.05)
**Chronic cardiac disease**	274825 (2.9)	2446 (0.89)	758 (0.28)	191 (0.07)	238 (0.09)	1825 (0.66)	260386 (2.8)	7115 (2.73)	396 (0.15)	133 (0.05)	130 (0.05)	811 (0.31)
**Diabetes**												
No diabetes	8877057 (95.1)	46639 (0.53)	4892 (0.06)	1131 (0.01)	793 (0.01)	8504 (0.10)	8811895 (95.1)	227899 (2.59)	2783 (0.03)	778 (0.01)	387 (0.00)	3468 (0.04)
Type 1, controlled	13036 (0.1)	82 (0.63)	21 (0.16)	7 (0.05)	6 (0.05)	68 (0.52)	13740 (0.1)	336 (2.45)	REDACTED	REDACTED	REDACTED	29 (0.21)
Type 1, uncontrolled	39054 (0.4)	313 (0.80)	78 (0.20)	23 (0.06)	21 (0.05)	191 (0.49)	38109 (0.4)	1042 (2.73)	32 (0.08)	15 (0.04)	6 (0.02)	86 (0.23)
Type 2, controlled	224926 (2.4)	2300 (1.02)	650 (0.29)	193 (0.09)	178 (0.08)	913 (0.41)	221590 (2.4)	6556 (2.96)	371 (0.17)	127 (0.06)	92 (0.04)	438 (0.20)
Type 2, uncontrolled	175978 (1.9)	2187 (1.24)	722 (0.41)	240 (0.14)	214 (0.12)	877 (0.50)	177484 (1.9)	5752 (3.24)	419 (0.24)	173 (0.10)	101 (0.06)	374 (0.21)
Diabetes, no HbA1c	4341 (0.0)	39 (0.90)	11 (0.25)	7 (0.16)	7 (0.16)	27 (0.62)	4101 (0.0)	108 (2.63)	6 (0.15)	7 (0.17)	2 (0.05)	14 (0.34)
**Haematological cancer**												
Diagnosed < 1 year ago	2427 (0.0)	46 (1.90)	11 (0.45)	REDACTED	7 (0.29)	80 (3.30)	2232 (0.0)	57 (2.55)	7 (0.31)	REDACTED	REDACTED	31 (1.39)
Diagnosed 1-4.9 years ago	7583 (0.1)	103 (1.36)	45 (0.59)	9 (0.12)	15 (0.20)	113 (1.49)	7103 (0.1)	171 (2.41)	14 (0.20)	8 (0.11)	9 (0.13)	23 (0.32)
Diagnosed ≥5 years ago	19774 (0.2)	140 (0.71)	40 (0.20)	15 (0.08)	14 (0.07)	106 (0.54)	19067 (0.2)	462 (2.42)	23 (0.12)	9 (0.05)	14 (0.07)	51 (0.27)
**Non-haematological cancer**												
Diagnosed < 1 year ago	22851 (0.2)	210 (0.92)	57 (0.25)	7 (0.03)	32 (0.14)	1248 (5.46)	20699 (0.2)	477 (2.30)	28 (0.14)	9 (0.04)	17 (0.08)	426 (2.06)

Diagnosed 1-4.9 years ago	66072 (0.7)	434 (0.66)	106 (0.16)	19 (0.03)	48 (0.07)	1340 (2.03)	61958 (0.7)	1488 (2.40)	51 (0.08)	16 (0.03)	24 (0.04)	496 (0.80)
Diagnosed ≥5 years ago	116131 (1.2)	715 (0.62)	138 (0.12)	21 (0.02)	49 (0.04)	757 (0.65)	110193 (1.2)	2569 (2.33)	74 (0.07)	21 (0.02)	22 (0.02)	320 (0.29)
**Reduced kidney function**												
Estimated GFR 30-60	96030 (1.0)	858 (0.89)	291 (0.30)	90 (0.09)	128 (0.13)	690 (0.72)	95475 (1.0)	2312 (2.42)	130 (0.14)	62 (0.06)	52 (0.05)	302 (0.32)
Estimated GFR <30	9342 (0.1)	352 (3.77)	140 (1.50)	37 (0.40)	67 (0.72)	322 (3.45)	9104 (0.1)	360 (3.95)	55 (0.60)	26 (0.29)	21 (0.23)	135 (1.48)
**End-stage renal disease***	11510 (0.1)	374 (3.25)	157 (1.36)	49 (0.43)	50 (0.43)	195 (1.69)	10787 (0.1)	396 (3.67)	56 (0.52)	30 (0.28)	18 (0.17)	90 (0.83)
**Chronic Liver disease**	54410 (0.6)	557 (1.02)	156 (0.29)	25 (0.05)	65 (0.12)	1005 (1.85)	51834 (0.6)	1202 (2.32)	50 (0.10)	15 (0.03)	34 (0.07)	458 (0.88)
**Stroke/dementia**	76724 (0.8)	768 (1.00)	246 (0.32)	64 (0.08)	91 (0.12)	731 (0.95)	72174 (0.8)	1762 (2.44)	103 (0.14)	32 (0.04)	39 (0.05)	321 (0.44)
**Other neurological disease**	66083 (0.7)	486 (0.74)	165 (0.25)	20 (0.03)	66 (0.10)	416 (0.63)	64030 (0.7)	1372 (2.14)	74 (0.12)	10 (0.02)	23 (0.04)	177 (0.28)
**Solid organ transplant****	2210 (0.0)	37 (1.67)	11 (0.50)	REDACTED	REDACTED	31 (1.40)	2122 (0.0)	64 (3.02)	8 (0.38)	REDACTED	REDACTED	9 (0.42)
**Asplenia**	11363 (0.1)	107 (0.94)	22 (0.19)	10 (0.09)	REDACTED	89 (0.78)	10883 (0.1)	247 (2.27)	13 (0.12)	REDACTED	REDACTED	43 (0.40)
**Rheumatoid/Lupus/ Psoriasis**	421447 (4.5)	2603 (0.62)	452 (0.11)	120 (0.03)	98 (0.02)	799 (0.19)	412685 (4.5)	11255 (2.73)	255 (0.06)	80 (0.02)	60 (0.01)	363 (0.09)
**Other immunosuppressive condition**	33165 (0.4)	365 (1.10)	47 (0.14)	18 (0.05)	21 (0.06)	156 (0.47)	33238 (0.4)	799 (2.40)	11 (0.03)	REDACTED	9 (0.03)	57 (0.17)
**Shielding*****	1976741 (21.2)	12740 (0.64)	2284 (0.12)	548 (0.03)	532 (0.03)	6589 (0.33)	1944911 (21.0)	53970 (2.77)	1399 (0.07)	429 (0.02)	290 (0.01)	2922 (0.15)

*End-stage renal disease includes on dialysis or having had a kidney transplant
**All solid organ transplants, excluding kidney
***Shielding includes organ transplant recipients, renal replacement therapy, haematological cancers, non-haematological cancers, immunodeficiencies/asplenia and severe respiratory conditions.

Table A4. Source of diagnoses for the outcome of SARS-CoV-2 infection by wave and exposure status

	% of infections that came from each source	
	**Living with children**	**Not living with children**
**Wave 1**		
SGSS test positive	30	32
PCR positive test code	51	45
Sequelae code	1	1
Clinical diagnosis code	18	22
**Wave 2**		
SGSS test positive	25	25
PCR positive test code	73	73
Sequelae code	1	1
Clinical diagnosis code	2	2

Note: Due to the way OpenSAFELY data is extracted and analysed we are not able to provide the exact information for the cohorts used (for example, if a patient has subsequently changed GP practice and moved to a non-TPP surgery, for information governance reasons their data is no longer available). However, we have analysed this information from what is currently held on the server for the same time periods and these will be very similar to the cohorts in our analysis.

Table A5. Cohort description by outcomes (evidence of SARS-CoV-2 infection recorded in primary care, COVID-19 outcomes and non-COVID 19 deaths) for adults >65 years in wave 1 and wave 2 of the UK pandemic

	Wave 1 (1st February to 31st August 2020)						Wave 2 (1st February to 31st August 2020)					
		Number (%) within stratum)						Number (%) within stratum)				
	N (column %)	Recorded SARS-CoV-2 infection	COVID-19 hospital admissions	COVID-19 ICU admissions	COVID-19 deaths	Non- COVID-19 deaths	N (column %)	Recorded SARS-CoV-2 infection	COVID-19 hospital admissions	COVID-19 ICU admissions	COVID-19 deaths	Non- COVID-19 deaths
**Total**	2684524 (100.0)	18893 (0.70)	8560 (0.32)	732 (0.03)	6507 (0.24)	45940 (1.71)	2706505 (100.0)	34519 (1.28)	4280 (0.16)	784 (0.03)	4108 (0.15)	21796 (0.81)
**Age**												
>65-70	633913 (23.6)	2723 (0.43)	1045 (0.16)	240 (0.04)	475 (0.07)	3682 (0.58)	632027 (23.4)	8124 (1.29)	573 (0.09)	213 (0.03)	286 (0.05)	1654 (0.26)
70-80	1357522 (50.6)	7153 (0.53)	3239 (0.24)	428 (0.03)	2032 (0.15)	14970 (1.10)	1375657 (50.8)	14938 (1.09)	1809 (0.13)	459 (0.03)	1275 (0.09)	7153 (0.52)
80+	693089 (25.8)	9017 (1.30)	4276 (0.62)	64 (0.01)	4000 (0.58)	27288 (3.94)	698821 (25.8)	11457 (1.64)	1898 (0.27)	112 (0.02)	2547 (0.36)	12989 (1.86)
**Sex**												
Female	1445097 (53.8)	9065 (0.63)	3593 (0.25)	210 (0.01)	2688 (0.19)	22087 (1.53)	1457578 (53.9)	17650 (1.21)	1858 (0.13)	232 (0.02)	1666 (0.11)	10433 (0.72)
Male	1239427 (46.2)	9828 (0.79)	4967 (0.40)	522 (0.04)	3819 (0.31)	23853 (1.92)	1248927 (46.1)	16869 (1.35)	2422 (0.19)	552 (0.04)	2442 (0.20)	11363 (0.91)
**BMI (kg/m2)**												
<18.5	48518 (1.8)	710 (1.46)	263 (0.54)	REDACTED	253 (0.52)	2985 (6.15)	44591 (1.6)	709 (1.59)	98 (0.22)	REDACTED	146 (0.33)	1395 (3.13)
18.5-24.9	800412 (29.8)	5697 (0.71)	2405 (0.30)	122 (0.02)	1996 (0.25)	17122 (2.14)	793996 (29.3)	8798 (1.11)	994 (0.13)	98 (0.01)	1178 (0.15)	7870 (0.99)
25-29.9	987076 (36.8)	6129 (0.62)	2764 (0.28)	280 (0.03)	1952 (0.20)	13160 (1.33)	994595 (36.7)	12485 (1.26)	1401 (0.14)	280 (0.03)	1265 (0.13)	6496 (0.65)
30-34.9 (Obese class I)	479258 (17.9)	3500 (0.73)	1695 (0.35)	188 (0.04)	1190 (0.25)	6105 (1.27)	485856 (18.0)	7227 (1.49)	1002 (0.21)	228 (0.05)	803 (0.17)	2942 (0.61)
35-39.9 (Obese class II)	156751 (5.8)	1349 (0.86)	704 (0.45)	81 (0.05)	488 (0.31)	2259 (1.44)	159518 (5.9)	2601 (1.63)	407 (0.26)	93 (0.06)	314 (0.20)	1030 (0.65)
≥40 (Obese class III)	61135 (2.3)	605 (0.99)	304 (0.50)	23 (0.04)	218 (0.36)	1011 (1.65)	62493 (2.3)	1128 (1.81)	190 (0.30)	49 (0.08)	171 (0.27)	512 (0.82)
Missing	151374 (5.6)	903 (0.60)	425 (0.28)	34 (0.02)	410 (0.27)	3298 (2.18)	165456 (6.1)	1571 (0.95)	188 (0.11)	34 (0.02)	231 (0.14)	1551 (0.94)
**Smoking**												
Never	1060734 (39.5)	6317 (0.60)	2612 (0.25)	224 (0.02)	1906 (0.18)	13564 (1.28)	1073743 (39.7)	12321 (1.15)	1207 (0.11)	216 (0.02)	1122 (0.10)	6413 (0.60)
Former	1395189 (52.0)	11234 (0.81)	5401 (0.39)	471 (0.03)	4126 (0.30)	26874 (1.93)	1404161 (51.9)	20122 (1.43)	2830 (0.20)	539 (0.04)	2693 (0.19)	12819 (0.91)

Current	222197 (8.3)	1312 (0.59)	537 (0.24)	33 (0.01)	464 (0.21)	5423 (2.44)	222251 (8.2)	2043 (0.92)	239 (0.11)	28 (0.01)	287 (0.13)	2539 (1.14)
Missing	6404 (0.2)	30 (0.47)	10 (0.16)	REDACTED	11 (0.17)	79 (1.23)	6350 (0.2)	33 (0.52)	REDACTED	REDACTED	6 (0.09)	25 (0.39)
**Ethnicity**												
White	2533944 (94.4)	16803 (0.66)	7448 (0.29)	603 (0.02)	5796 (0.23)	43761 (1.73)	2552971 (94.3)	31054 (1.22)	3731 (0.15)	669 (0.03)	3701 (0.14)	20786 (0.81)
Mixed	9970 (0.4)	94 (0.94)	51 (0.51)	8 (0.08)	35 (0.35)	133 (1.33)	10249 (0.4)	117 (1.14)	13 (0.13)	REDACTED	19 (0.19)	78 (0.76)
South Asian	91000 (3.4)	1407 (1.55)	709 (0.78)	73 (0.08)	467 (0.51)	1362 (1.50)	92732 (3.4)	2791 (3.01)	438 (0.47)	88 (0.09)	310 (0.33)	611 (0.66)
Black	27818 (1.0)	397 (1.43)	248 (0.89)	29 (0.10)	145 (0.52)	443 (1.59)	28209 (1.0)	323 (1.15)	61 (0.22)	17 (0.06)	54 (0.19)	207 (0.73)
Other	21792 (0.8)	192 (0.88)	104 (0.48)	19 (0.09)	64 (0.29)	241 (1.11)	22344 (0.8)	234 (1.05)	37 (0.17)	8 (0.04)	24 (0.11)	114 (0.51)
**IMD quintile**												
1 (least deprived)	651069 (24.3)	3333 (0.51)	1385 (0.21)	143 (0.02)	1101 (0.17)	8972 (1.38)	668867 (24.7)	6175 (0.92)	633 (0.09)	127 (0.02)	677 (0.10)	4469 (0.67)
2	629037 (23.4)	3795 (0.60)	1626 (0.26)	145 (0.02)	1299 (0.21)	9782 (1.56)	636250 (23.5)	6674 (1.05)	693 (0.11)	118 (0.02)	709 (0.11)	4685 (0.74)
3	580534 (21.6)	3797 (0.65)	1753 (0.30)	162 (0.03)	1314 (0.23)	9752 (1.68)	574847 (21.2)	6480 (1.13)	762 (0.13)	156 (0.03)	736 (0.13)	4588 (0.80)
4	477447 (17.8)	4163 (0.87)	1948 (0.41)	161 (0.03)	1430 (0.30)	9234 (1.93)	479967 (17.7)	7360 (1.53)	899 (0.19)	180 (0.04)	922 (0.19)	4295 (0.89)
5 (most deprived)	346437 (12.9)	3805 (1.10)	1848 (0.53)	121 (0.03)	1363 (0.39)	8200 (2.37)	346574 (12.8)	7830 (2.26)	1293 (0.37)	203 (0.06)	1064 (0.31)	3759 (1.08)
**Total number adults in household**												
1	913998 (34.0)	8180 (0.89)	3690 (0.40)	184 (0.02)	3028 (0.33)	21246 (2.32)	921594 (34.1)	12322 (1.34)	1680 (0.18)	239 (0.03)	1859 (0.20)	9846 (1.07)
2	1384390 (51.6)	7508 (0.54)	3421 (0.25)	388 (0.03)	2459 (0.18)	18799 (1.36)	1390726 (51.4)	15600 (1.12)	1813 (0.13)	378 (0.03)	1645 (0.12)	9189 (0.66)
≥3	386136 (14.4)	3205 (0.83)	1449 (0.38)	160 (0.04)	1020 (0.26)	5895 (1.53)	394185 (14.6)	6597 (1.67)	787 (0.20)	167 (0.04)	604 (0.15)	2761 (0.70)
**Blood pressure**												
Normal	309796 (11.5)	3282 (1.06)	1522 (0.49)	84 (0.03)	1334 (0.43)	10455 (3.37)	310503 (11.5)	4830 (1.56)	636 (0.20)	89 (0.03)	754 (0.24)	5004 (1.61)
Elevated	417855 (15.6)	3138 (0.75)	1485 (0.36)	114 (0.03)	1176 (0.28)	7693 (1.84)	422947 (15.6)	5614 (1.33)	695 (0.16)	117 (0.03)	754 (0.18)	3497 (0.83)
High Stage 1	990139 (36.9)	6163 (0.62)	2814 (0.28)	264 (0.03)	1969 (0.20)	13670 (1.38)	1011992 (37.4)	12469 (1.23)	1508 (0.15)	308 (0.03)	1317 (0.13)	6420 (0.63)
High Stage 2	952842 (35.5)	6279 (0.66)	2727 (0.29)	265 (0.03)	2017 (0.21)	14026 (1.47)	946777 (35.0)	11544 (1.22)	1435 (0.15)	267 (0.03)	1275 (0.13)	6828 (0.72)
Missing	13892 (0.5)	31 (0.22)	12 (0.09)	REDACTED	11 (0.08)	96 (0.69)	14286 (0.5)	62 (0.43)	6 (0.04)	REDACTED	8 (0.06)	47 (0.33)
**High bp or diagnosed hypertension**	1859250 (69.3)	14515 (0.78)	6673 (0.36)	542 (0.03)	5111 (0.27)	35289 (1.90)	1858019 (68.7)	25105 (1.35)	3340 (0.18)	573 (0.03)	3254 (0.18)	16794 (0.90)

Comorbidities												
**Chronic respiratory disease ex asthma**	334940 (12.5)	4581 (1.37)	2339 (0.70)	134 (0.04)	1659 (0.50)	12241 (3.65)	324691 (12.0)	6356 (1.96)	1313 (0.40)	187 (0.06)	1189 (0.37)	5638 (1.74)
**Asthma**	362235 (13.5)	3209 (0.89)	1450 (0.40)	117 (0.03)	993 (0.27)	6166 (1.70)	366433 (13.5)	5610 (1.53)	771 (0.21)	135 (0.04)	647 (0.18)	3016 (0.82)
**Chronic cardiac disease**	571371 (21.3)	7262 (1.27)	3520 (0.62)	172 (0.03)	2805 (0.49)	19427 (3.40)	551840 (20.4)	10384 (1.88)	1735 (0.31)	235 (0.04)	1962 (0.36)	9142 (1.66)
**Diabetes**												
No diabetes	2201205 (82.0)	12799 (0.58)	5512 (0.25)	510 (0.02)	4173 (0.19)	33039 (1.50)	2221833 (82.1)	25566 (1.15)	2774 (0.12)	506 (0.02)	2625 (0.12)	15723 (0.71)
Type 1, controlled	3563 (0.1)	50 (1.40)	26 (0.73)	REDACTED	20 (0.56)	125 (3.51)	3710 (0.1)	57 (1.54)	9 (0.24)	REDACTED	12 (0.32)	64 (1.73)
Type 1, uncontrolled	7408 (0.3)	127 (1.71)	54 (0.73)	REDACTED	47 (0.63)	273 (3.69)	7352 (0.3)	135 (1.84)	21 (0.29)	REDACTED	27 (0.37)	110 (1.50)
Type 2, controlled	323854 (12.1)	3770 (1.16)	1803 (0.56)	125 (0.04)	1427 (0.44)	8447 (2.61)	323947 (12.0)	5616 (1.73)	878 (0.27)	157 (0.05)	882 (0.27)	3963 (1.22)
Type 2, uncontrolled	146537 (5.5)	2116 (1.44)	1145 (0.78)	91 (0.06)	829 (0.57)	3990 (2.72)	147893 (5.5)	3101 (2.10)	595 (0.40)	116 (0.08)	557 (0.38)	1897 (1.28)
Diabetes, no HbA1c	1957 (0.1)	31 (1.58)	20 (1.02)	REDACTED	11 (0.56)	66 (3.37)	1770 (0.1)	44 (2.49)	REDACTED	REDACTED	REDACTED	39 (2.20)
**Haematological cancer**												
Diagnosed < 1 year ago	3839 (0.1)	74 (1.93)	36 (0.94)	6 (0.16)	31 (0.81)	333 (8.67)	3499 (0.1)	53 (1.51)	9 (0.26)	REDACTED	10 (0.29)	132 (3.77)
Diagnosed 1-4.9 years ago	11638 (0.4)	184 (1.58)	105 (0.90)	15 (0.13)	83 (0.71)	572 (4.91)	11138 (0.4)	191 (1.71)	37 (0.33)	6 (0.05)	43 (0.39)	265 (2.38)
Diagnosed ≥5 years ago	23786 (0.9)	308 (1.29)	134 (0.56)	8 (0.03)	109 (0.46)	871 (3.66)	23008 (0.9)	384 (1.67)	72 (0.31)	16 (0.07)	80 (0.35)	408 (1.77)
**Non-haematological cancer**												
Diagnosed < 1 year ago	32334 (1.2)	425 (1.31)	202 (0.62)	7 (0.02)	181 (0.56)	3614 (11.18)	29009 (1.1)	434 (1.50)	62 (0.21)	REDACTED	87 (0.30)	1199 (4.13)
Diagnosed 1-4.9 years ago	95696 (3.6)	805 (0.84)	383 (0.40)	20 (0.02)	314 (0.33)	4205 (4.39)	92748 (3.4)	1257 (1.36)	172 (0.19)	30 (0.03)	203 (0.22)	1749 (1.89)
Diagnosed ≥5 years ago	254667 (9.5)	2244 (0.88)	1018 (0.40)	61 (0.02)	783 (0.31)	6780 (2.66)	248878 (9.2)	3350 (1.35)	449 (0.18)	69 (0.03)	494 (0.20)	3131 (1.26)
**Reduced kidney function**												
Estimated GFR 30-60	563148 (21.0)	6172 (1.10)	2963 (0.53)	156 (0.03)	2552 (0.45)	16311 (2.90)	560736 (20.7)	8830 (1.57)	1470 (0.26)	202 (0.04)	1661 (0.30)	7765 (1.38)
Estimated GFR <30	41148 (1.5)	1218 (2.96)	640 (1.56)	17 (0.04)	597 (1.45)	3856 (9.37)	40212 (1.5)	1143 (2.84)	237 (0.59)	24 (0.06)	413 (1.03)	1892 (4.71)
**End-stage renal disease***	6885 (0.3)	334 (4.85)	174 (2.53)	13 (0.19)	121 (1.76)	537 (7.80)	6285 (0.2)	213 (3.39)	37 (0.59)	10 (0.16)	43 (0.68)	244 (3.88)
**Chronic Liver disease**	26389 (1.0)	380 (1.44)	173 (0.66)	8 (0.03)	138 (0.52)	1160 (4.40)	25834 (1.0)	503 (1.95)	77 (0.30)	13 (0.05)	89 (0.34)	586 (2.27)

**Stroke/dementia**	204093 (7.6)	3502 (1.72)	1581 (0.77)	63 (0.03)	1370 (0.67)	9118 (4.47)	191057 (7.1)	4133 (2.16)	659 (0.34)	57 (0.03)	827 (0.43)	4167 (2.18)
**Other neurological disease**	54121 (2.0)	938 (1.73)	430 (0.79)	13 (0.02)	388 (0.72)	2253 (4.16)	51397 (1.9)	1091 (2.12)	158 (0.31)	17 (0.03)	209 (0.41)	1063 (2.07)
**Solid organ transplant****	872 (0.0)	21 (2.41)	8 (0.92)	REDACTED	7 (0.80)	35 (4.01)	852 (0.0)	15 (1.76)	6 (0.70)	REDACTED	REDACTED	16 (1.88)
**Asplenia**	6733 (0.3)	80 (1.19)	34 (0.50)	REDACTED	17 (0.25)	205 (3.04)	6639 (0.2)	107 (1.61)	15 (0.23)	REDACTED	15 (0.23)	107 (1.61)
**Rheumatoid/Lupus/ Psoriasis**	208673 (7.8)	1915 (0.92)	861 (0.41)	68 (0.03)	625 (0.30)	4222 (2.02)	208797 (7.7)	3040 (1.46)	451 (0.22)	80 (0.04)	405 (0.19)	1956 (0.94)
**Other immunosuppressive condition**	5397 (0.2)	77 (1.43)	34 (0.63)	REDACTED	29 (0.54)	221 (4.09)	5658 (0.2)	86 (1.52)	12 (0.21)	REDACTED	16 (0.28)	112 (1.98)
**Shielding*****	956589 (35.6)	9662 (1.01)	4547 (0.48)	317 (0.03)	3330 (0.35)	29076 (3.04)	941453 (34.8)	14751 (1.57)	2320 (0.25)	359 (0.04)	2244 (0.24)	13841 (1.47)

*End-stage renal disease includes on dialysis or having had a kidney transplant
**All solid organ transplants, excluding kidney
***Shielding includes organ transplant recipients, renal replacement therapy, haematological cancers, non-haematological cancers, immunodeficiencies/asplenia and severe respiratory condition

Table A6. Hazard Ratios (HRs) for outcomes in wave 1 and wave 2 (a) evidence of SARS-CoV-2 infection recorded in primary care, (b) COVID-19 hospital admission, (c) COVID-19 ICU admission, (d) COVID-19 death and (e) non-COVID-19 death), stratified by age.

(a) Recorded SARS-CoV-2 infection

	Wave 1 - Recorded SARS-CoV-2 infection						Wave 2 - Recorded SARS-CoV-2 infection					
				HR (95% CI)						HR (95% CI)		
Children in the household	N	Person years follow-up	Rate (per 100,000 person years)	Age-sex adjusted	+ Demographic adjusted*	+ Comorbidity adjusted**	N	Person years follow-up	Rate (per 100,000 person years)	Age-sex adjusted	+ Demographic adjusted*	+ Comorbidity adjusted**
**Adults 65 years and under**												
None	30415	3363742	904.2	1.00 (ref)	1.00 (ref)	1.00 (ref)	137612	1694805	8119.64	1.00 (ref)	1.00 (ref)	1.00 (ref)
Only children 0-11 years	10883	1104266	985.54	1.08 (1.05-1.10)	0.97 (0.95-1.00)	0.98 (0.96-1.01)	50293	538680	9336.35	1.11 (1.10-1.12)	1.06 (1.05-1.07)	1.06 (1.05-1.08)
Only children aged ≥ 12 years	5637	500507	1126.26	1.16 (1.13-1.20)	1.04 (1.01-1.08)	1.05 (1.01-1.08)	30192	259848	11619.1	1.30 (1.28-1.31)	1.22 (1.20-1.24)	1.22 (1.20-1.24)
Children aged 0-11 and ≥12	4625	394047	1173.72	1.22 (1.17-1.26)	1.00 (0.96-1.04)	1.01 (0.97-1.05)	23596	202433	11656.2	1.33 (1.31-1.36)	1.25 (1.23-1.27)	1.25 (1.23-1.27)
**Number of children aged 0-11 years in household**												
1 child 0-11 years	5444	553847	982.94	1.00 (ref)	1.00 (ref)	1.00 (ref)	25061	275336	9101.98	1.00 (ref)	1.00 (ref)	1.00 (ref)
2 children 0-11 years	4133	427480	966.83	0.99 (0.95-1.04)	0.99 (0.94-1.03)	0.99 (0.95-1.04)	19433	206410	9414.75	1.05 (1.02-1.07)	1.04 (1.02-1.06)	1.04 (1.02-1.06)
3 children 0-11 years	1077	99077	1087.03	1.09 (1.01-1.17)	0.95 (0.88-1.02)	0.95 (0.88-1.02)	4676	46184	10124.69	1.08 (1.04-1.12)	1.05 (1.01-1.09)	1.05 (1.01-1.09)
≥4 children 0-11 years	229	23861	959.72	0.94 (0.81-1.08)	0.75 (0.65-0.87)	0.76 (0.65-0.88)	1123	10750	10446.91	1.07 (1.00-1.15)	1.04 (0.97-1.11)	1.04 (0.97-1.11)
**Adults over 65 years**												
None	18078	1483331	1218.74	1.00 (ref)	1.00 (ref)	1.00 (ref)	32652	765224	4266.98	1.00 (ref)	1.00 (ref)	1.00 (ref)
Only Children 0-11 years	401	25307	1584.51	1.39 (1.25-1.54)	1.06 (0.95-1.18)	1.04 (0.93-1.16)	891	12906	6903.98	1.68 (1.57-1.81)	1.15 (1.07-1.25)	1.15 (1.06-1.24)
Only children aged ≥ 12 years	232	16673	1391.44	1.15 (1.01-1.32)	0.93 (0.81-1.06)	0.92 (0.80-1.06)	571	8707	6557.61	1.57 (1.44-1.72)	1.17 (1.07-1.29)	1.16 (1.06-1.27)
Children aged 0-11 and ≥12	182	8746	2081.06	1.72 (1.47-2.00)	1.19 (1.02-1.39)	1.17 (1.00-1.37)	405	4607	8790.69	2.07 (1.86-2.30)	1.29 (1.15-1.44)	1.27 (1.14-1.42)
**Number of children aged 0-11 years in household**												
1 child 0-11 years	224	14959	1497.48	1.00 (ref)	1.00 (ref)	1.00 (ref)	467	7773	6008.26	1.00 (ref)	1.00 (ref)	1.00 (ref)
2 children 0-11 years	118	8061	1463.91	0.95 (0.75-1.21)	0.92 (0.72-1.16)	0.92 (0.72-1.17)	312	4052	7699.9	1.25 (1.07-1.46)	1.15 (0.98-1.34)	1.15 (0.98-1.34)
3 children 0-11 years	50	1909	2619.12	1.56 (1.13-2.15)	1.29 (0.93-1.79)	1.29 (0.93-1.80)	93	906	10263.71	1.48 (1.17-1.87)	1.20 (0.94-1.52)	1.21 (0.95-1.53)
≥4 children 0-11 years	9	379	2372.99	1.31 (0.67-2.54)	1.02 (0.52-2.01)	1.09 (0.55-2.17)	19	175	10865.94	1.44 (0.87-2.39)	1.11 (0.67-1.85)	1.14 (0.68-1.89)

*Demographic adjusted model: Adjusted for age, sex, ethnicity, number adults in household, IMD, BMI, smoking.

**Comorbidity-adjusted model: Adjusted for age, sex, ethnicity, number adults in household, IMD, BMI, smoking, hypertension or high blood pressure, chronic respiratory disease, asthma, cancer, chronic liver disease, stroke or dementia, other neurological disease, reduced kidney function, end-stage renal disease, solid organ transplant, asplenia, rheumatoid, lupus or psoriasis, other immunosuppressive condition.

(b) COVID-19 hospital admissions

	Wave 1 - COVID-19 hospital admissions						Wave 2 - COVID-19 hospital admissions					
				HR (95% CI)						HR (95% CI)		
	N	Person years follow-up	Rate (per 100,000 person years)	Age-sex adjusted	+ Demographic adjusted*	+ Comorbidity adjusted**	N	Person years follow-up	Rate (per 100,000 person years)	Age-sex adjusted	+ Demographic adjusted*	+ Comorbidity adjusted**
**Adults 65 years and under**												
None	4319	3370737	128.13	1.00 (ref)	1.00 (ref)	1.00 (ref)	2292	1426755	160.64	1.00 (ref)	1.00 (ref)	1.00 (ref)
Only children aged 0-11 years	874	1106851	78.96	1.03 (0.95-1.12)	0.91 (0.84-0.98)	0.92 (0.85-1.00)	553	453698	121.89	1.34 (1.21-1.49)	1.17 (1.06-1.30)	1.18 (1.06-1.31)
Only children aged ⩾ 12 years	670	501807	133.52	1.17 (1.07-1.28)	1.05 (0.96-1.14)	1.06 (0.97-1.16)	424	219433	193.23	1.41 (1.26-1.57)	1.25 (1.12-1.39)	1.26 (1.12-1.40)
Children aged 0-11 and ≥12	511	395082	129.34	1.39 (1.26-1.53)	1.09 (0.99-1.20)	1.12 (1.01-1.23)	346	170861	202.5	1.85 (1.64-2.08)	1.42 (1.26-1.61)	1.44 (1.28-1.63)
**Number of children aged 0-11 years in household**												
1 child 0-11 years	445	555137	80.16	1.00 (ref)	1.00 (ref)	1.00 (ref)	274	231874	118.17	1.00 (ref)	1.00 (ref)	1.00 (ref)
2 children 0-11 years	312	428486	72.81	0.98 (0.85-1.14)	0.98 (0.84-1.14)	0.99 (0.85-1.15)	203	173826	116.78	1.08 (0.90-1.30)	1.10 (0.91-1.32)	1.10 (0.91-1.33)
3 children 0-11 years	97	99319	97.66	1.32 (1.06-1.66)	1.11 (0.88-1.39)	1.10 (0.88-1.39)	56	38934	143.83	1.30 (0.98-1.74)	1.08 (0.81-1.45)	1.07 (0.80-1.43)
⩾4 children 0-11 years	20	23908	83.65	1.16 (0.74-1.81)	0.87 (0.55-1.36)	0.89 (0.56-1.39)	20	9064	220.66	1.98 (1.25-3.12)	1.46 (0.92-2.31)	1.46 (0.93-2.31)
**Adults over 65 years**												
None	8172	1485541	550.1	1.00 (ref)	1.00 (ref)	1.00 (ref)	4056	640805	632.95	1.00 (ref)	1.00 (ref)	1.00 (ref)
Only children aged 0-11 years	186	25357	733.52	1.41 (1.21-1.63)	1.09 (0.93-1.27)	1.06 (0.91-1.24)	96	10842	885.46	1.68 (1.37-2.06)	0.97 (0.79-1.21)	0.96 (0.77-1.19)
Only children aged ⩾ 12 years	119	16698	712.64	1.28 (1.07-1.53)	1.04 (0.86-1.25)	1.03 (0.86-1.25)	69	7309	944.06	1.64 (1.29-2.09)	1.06 (0.82-1.36)	1.04 (0.81-1.34)
Children aged 0-11 and ≥12	83	8767	946.69	1.68 (1.35-2.10)	1.19 (0.95-1.49)	1.16 (0.92-1.45)	56	3878	1444.22	2.47 (1.89-3.24)	1.23 (0.93-1.62)	1.19 (0.90-1.58)
**Number of children aged 0-11 years in household**												
1 child 0-11 years	97	14988	647.18	1.00 (.- .)	1.00 (.- .)	1.00 (.- .)	47	6522	720.64	1.00 (.- .)	1.00 (.- .)	1.00 (.- .)
2 children 0-11 years	59	8075	730.67	1.10 (0.79-1.52)	1.07 (0.77-1.50)	1.06 (0.75-1.48)	33	3407	968.48	1.29 (0.83-2.02)	1.15 (0.73-1.79)	1.12 (0.71-1.76)
3 children 0-11 years	27	1913	1411.24	1.93 (1.26-2.94)	1.64 (1.06-2.53)	1.61 (1.04-2.49)	12	765	1568.59	1.78 (0.93-3.41)	1.32 (0.68-2.54)	1.37 (0.69-2.71)
⩾4 children 0-11 years	REDACT	381	787.34	1.01 (0.32-3.19)	0.82 (0.26-2.61)	0.81 (0.25-2.64)	REDACTED	147	2712.59	2.68 (0.94-7.64)	1.99 (0.69-5.76)	2.08 (0.70-6.18)

*Demographic adjusted model: Adjusted for age, sex, ethnicity, number adults in household, IMD, BMI, smoking.
**Comorbidity-adjusted model: Adjusted for age, sex, ethnicity, number adults in household, IMD, BMI, smoking, hypertension or high blood pressure, chronic respiratory disease, asthma, cancer, chronic liver disease, stroke or dementia, other neurological disease, reduced kidney function, end-stage renal disease, solid organ transplant, asplenia, rheumatoid, lupus or psoriasis, other immunosuppressive condition.

c) **COVID-19 ICU admission**

	**Wave 1 - COVID-19 ICU admission**						**Wave 2 - COVID-19 ICU admission**					
				**HR (95% CI)**						**HR (95% CI)**		
	**N**	**Person years follow-up**	**Rate (per 100,000**	**Age-sex adjusted**	**+ Demographic adjusted***	**+ Comorbidity adjusted****	**N**	**Person years follow-up**	**Rate (per 100,000 person years)**	**Age-sex adjusted**	**+ Demographic adjusted***	**+ Comorbidity adjusted****
**Adults 65 years and under**												
None	1133	3371778	33.6	1.00 (ref)	1.00 (ref)	1.00 (ref)	733	1710507	42.85	1.00 (ref)	1.00 (ref)	1.00 (ref)
Only children aged 0-11 years	193	1107083	17.43	1.09 (0.92-1.30)	0.90 (0.76-1.06)	0.91 (0.76-1.07)	160	544105	29.41	1.47 (1.21-1.78)	1.18 (0.98-1.42)	1.19 (0.98-1.43)
Only children aged ⩾ 12 years	167	501982	33.27	1.21 (1.02-1.44)	1.04 (0.87-1.23)	1.05 (0.88-1.24)	120	263187	45.6	1.35 (1.10-1.64)	1.13 (0.92-1.38)	1.13 (0.93-1.39)
Children aged 0-11 and ≥12	108	395222	27.33	1.36 (1.10-1.67)	0.98 (0.79-1.21)	1.00 (0.81-1.23)	88	204948	42.94	1.72 (1.36-2.17)	1.20 (0.95-1.53)	1.22 (0.96-1.55)
**Number of children aged 0-11 years in household**												
1 child 0-11 years	102	555254	18.37	1.00 (ref)	1.00 (ref)	1.00 (ref)	91	278056	32.73	1.00 (ref)	1.00 (ref)	1.00 (ref)
2 children 0-11 years	65	428570	15.17	0.96 (0.70-1.31)	0.96 (0.71-1.31)	0.97 (0.71-1.32)	47	208489	22.54	0.77 (0.54-1.10)	0.77 (0.55-1.09)	0.77 (0.55-1.10)
3 children 0-11 years	19	99347	19.12	1.25 (0.76-2.04)	1.00 (0.60-1.64)	1.00 (0.61-1.64)	19	46693	40.69	1.42 (0.86-2.32)	1.13 (0.69-1.87)	1.11 (0.67-1.84)
⩾4 children 0-11 years	7	23913	29.27	2.08 (0.97-4.44)	1.44 (0.67-3.07)	1.49 (0.70-3.18)	REDACTED	10868	27.6	1.00 (0.31-3.19)	0.69 (0.22-2.20)	0.69 (0.22-2.19)
**Adults over 65 years**												
None	684	1487082	46	1.00 (ref)	1.00 (ref)	1.00 (ref)	727	768349	94.62	1.00 (ref)	1.00 (ref)	1.00 (ref)
Only children aged 0-11 years	25	25393	98.45	1.51 (1.01-2.27)	1.10 (0.72-1.68)	1.09 (0.71-1.67)	28	12997	215.44	1.99 (1.36-2.91)	1.32 (0.88-1.98)	1.30 (0.87-1.95)
Only children aged ⩾ 12 years	16	16721	95.69	1.64 (1.00-2.70)	1.27 (0.76-2.13)	1.26 (0.75-2.12)	13	8763	148.35	1.42 (0.82-2.47)	1.05 (0.59-1.86)	1.04 (0.59-1.84)
Children aged 0-11 and ≥12	7	8783	79.7	1.32 (0.63-2.78)	0.87 (0.41-1.86)	0.87 (0.41-1.85)	16	4646	344.36	3.26 (1.98-5.38)	1.90 (1.13-3.19)	1.86 (1.11-3.14)
**Number of children aged 0-11 years in household**												
1 child 0-11 years	12	15010	79.95	1.00 (ref)	1.00 (ref)	1.00 (ref)	14	7819	179.06	1.00 (ref)	1.00 (ref)	1.00 (ref)
2 children 0-11 years	10	8085	123.69	1.50 (0.65-3.46)	1.37 (0.60-3.14)	1.47 (0.64-3.36)	8	4085	195.85	1.08 (0.45-2.57)	0.95 (0.40-2.25)	0.95 (0.41-2.21)
3 children 0-11 years	REDACTED	1917	104.34	1.19 (0.27-5.27)	0.93 (0.20-4.47)	0.90 (0.19-4.29)	6	917	654.44	3.18 (1.24-8.15)	2.28 (0.91-5.71)	2.49 (0.99-6.24)
⩾4 children 0-11 years	REDACTED	381	262.13	3.13 (0.39-25.01)	2.38 (0.29-19.30)	2.31 (0.27-20.12)	0	177	0	0.00 (.- .)	0.00 (0.00-0.00)	0.00 (0.00-0.00)

*Demographic adjusted model: Adjusted for age, sex, ethnicity, number adults in household, IMD, BMI, smoking.
**Comorbidity-adjusted model: Adjusted for age, sex, ethnicity, number adults in household, IMD, BMI, smoking, hypertension or high blood pressure, chronic respiratory disease, asthma, cancer, chronic liver disease, stroke or dementia, other neurological disease, reduced kidney function, end-stage renal disease, solid organ transplant, asplenia, rheumatoid, lupus or psoriasis, other immunosuppressive condition.

d) COVID-19 death

	Wave 1 - COVID-19 death						Wave 2 - COVID-19 death					
				HR (95% CI)						HR (95% CI)		
	N	Person years follow-up	Rate (per 100,000 person years)	Age-sex adjusted	+ Demographic adjusted*	+ Comorbidity adjusted**	N	Person years follow-up	Rate (per 100,000 person years)	Age-sex adjusted	+ Demographic adjusted*	+ Comorbidity adjusted**
**Adults 65 years and under**												
None	990	3372068	29.36	1.00 (ref)	1.00 (ref)	1.00 (ref)	492	1710567	28.76	1.00 (ref)	1.00 (ref)	1.00 (ref)
Only children aged 0-11 years	92	1107136	8.31	0.81 (0.64-1.03)	0.71 (0.56-0.89)	0.71 (0.57-0.90)	28	544119	5.15	0.59 (0.39-0.89)	0.52 (0.35-0.78)	0.52 (0.35-0.78)
Only children aged ≥ 12 years	90	502029	17.93	0.91 (0.73-1.14)	0.85 (0.68-1.07)	0.87 (0.69-1.09)	48	263197	18.24	0.99 (0.73-1.36)	0.97 (0.70-1.32)	0.97 (0.71-1.33)
Children aged 0-11 and ≥12	47	395252	11.89	0.92 (0.67-1.25)	0.73 (0.54-0.99)	0.76 (0.56-1.03)	23	204956	11.22	1.00 (0.64-1.54)	0.82 (0.53-1.27)	0.84 (0.54-1.30)
**Number of children aged 0-11 years in household**												
1 child 0-11 years	58	555281	10.45	1.00 (ref)	1.00 (ref)	1.00 (ref)	16	278063	5.75	1.00 (ref)	1.00 (ref)	1.00 (ref)
2 children 0-11 years	26	428589	6.07	0.73 (0.45-1.17)	0.74 (0.47-1.19)	0.74 (0.47-1.18)	8	208493	3.84	0.89 (0.37-2.12)	0.91 (0.39-2.12)	0.92 (0.40-2.12)
3 children 0-11 years	6	99352	6.04	0.73 (0.31-1.70)	0.60 (0.25-1.43)	0.59 (0.25-1.40)	REDACTED	46694	6.42	1.51 (0.43-5.26)	1.32 (0.37-4.73)	1.10 (0.30-4.06)
≥4 children 0-11 years	REDACTED	23915	8.36	1.09 (0.26-4.51)	0.80 (0.19-3.31)	0.83 (0.20-3.46)	REDACTED	10868	9.2	2.32 (0.29-18.36)	1.80 (0.21-15.28)	1.74 (0.19-16.06)
**Adults over 65 years**												
None	6294	1487196	423.21	1.00 (ref)	1.00 (ref)	1.00 (ref)	3934	768392	511.98	1.00 (ref)	1.00 (ref)	1.00 (ref)
Only children aged 0-11 years	101	25396	397.7	1.18 (0.97-1.44)	0.88 (0.71-1.08)	0.86 (0.70-1.06)	72	12999	553.89	1.63 (1.29-2.06)	1.11 (0.87-1.41)	1.09 (0.86-1.40)
Only children aged ≥ 12 years	66	16723	394.66	1.03 (0.81-1.32)	0.81 (0.63-1.04)	0.80 (0.62-1.03)	56	8764	638.98	1.59 (1.22-2.07)	1.17 (0.89-1.54)	1.14 (0.86-1.50)
Children aged 0-11 and ≥12	46	8784	523.67	1.40 (1.05-1.88)	0.94 (0.70-1.27)	0.92 (0.68-1.24)	46	4647	989.84	2.52 (1.88-3.37)	1.48 (1.09-2.02)	1.44 (1.05-1.97)
**Number of children aged 0-11 years in household**												
1 child 0-11 years	56	15012	373.05	1.00 (ref)	1.00 (ref)	1.00 (ref)	31	7820	396.44	1.00 (ref)	1.00 (ref)	1.00 (ref)
2 children 0-11 years	29	8086	358.65	0.94 (0.60-1.47)	0.90 (0.58-1.42)	0.90 (0.57-1.42)	32	4085	783.3	1.92 (1.18-3.15)	1.72 (1.06-2.81)	1.75 (1.07-2.87)
3 children 0-11 years	13	1917	678.18	1.54 (0.84-2.83)	1.24 (0.67-2.31)	1.26 (0.68-2.34)	6	917	654.14	1.37 (0.57-3.31)	0.92 (0.38-2.22)	0.92 (0.37-2.27)
≥4 children 0-11 years	REDACTED	382	786.3	1.56 (0.50-4.87)	1.20 (0.37-3.91)	1.10 (0.33-3.65)	REDACTED	177	1697.05	3.08 (0.91-10.40)	1.85 (0.53-6.49)	2.08 (0.57-7.56)

*Demographic adjusted model: Adjusted for age, sex, ethnicity, number adults in household, IMD, BMI, smoking.
**Comorbidity-adjusted model: Adjusted for age, sex, ethnicity, number adults in household, IMD, BMI, smoking, hypertension or high blood pressure, chronic respiratory disease, asthma, cancer, chronic liver disease, stroke or dementia, other neurological disease, reduced kidney function, end-stage renal disease, solid organ transplant, asplenia, rheumatoid, lupus or psoriasis, other immunosuppressive condition.

e) non-COVID-19 deaths

	Wave 1 - non-COVID-19 deaths						Wave 2 - non-COVID-19 deaths					
				HR (95% CI)						HR (95% CI)		
	N	Person years follow-up	Rate (per 100,000 person years)	Age-sex adjusted	+ Demographic adjusted*	+ Comorbidity adjusted**	N	Person years follow-up	Rate (per 100,000 person years)	Age-sex adjusted	+ Demographic adjusted*	+ Comorbidity adjusted**
**Adults 65 years and under**												
None	9033	3372068	267.88	1.00 (ref)	1.00 (ref)	1.00 (ref)	3746	1710567	218.99	1.00 (ref)	1.00 (ref)	1.00 (ref)
Only children aged 0-11 years	666	1107136	60.16	0.50 (0.46-0.55)	0.61 (0.56-0.66)	0.62 (0.57-0.68)	259	544119	47.6	0.55 (0.48-0.63)	0.64 (0.56-0.74)	0.66 (0.57-0.75)
Only children aged ⩾ 12 years	597	502029	118.92	0.59 (0.54-0.64)	0.72 (0.66-0.78)	0.73 (0.67-0.80)	264	263197	100.3	0.63 (0.55-0.71)	0.74 (0.65-0.84)	0.76 (0.67-0.87)
Children aged 0-11 and ≥12	284	395252	71.85	0.49 (0.43-0.55)	0.58 (0.51-0.66)	0.61 (0.54-0.69)	140	204956	68.31	0.63 (0.53-0.75)	0.73 (0.61-0.87)	0.77 (0.64-0.91)
**Number of children aged 0-11 years in household**												
1 child 0-11 years	391	555281	70.41	1.00 (ref)	1.00 (ref)	1.00 (ref)	152	278063	54.66	1.00 (ref)	1.00 (ref)	1.00 (ref)
2 children 0-11 years	222	428589	51.8	0.90 (0.77-1.07)	0.96 (0.81-1.14)	1.00 (0.84-1.18)	83	208493	39.81	0.85 (0.65-1.11)	0.89 (0.68-1.16)	0.91 (0.69-1.19)
3 children 0-11 years	47	99352	47.31	0.88 (0.65-1.20)	0.90 (0.66-1.22)	1.00 (0.73-1.36)	20	46694	42.83	0.97 (0.61-1.55)	0.97 (0.61-1.56)	1.07 (0.67-1.72)
⩾4 children 0-11 years	6	23915	25.09	0.51 (0.23-1.15)	0.48 (0.22-1.08)	0.57 (0.26-1.27)	REDACTED	10868	36.8	0.92 (0.34-2.49)	0.86 (0.31-2.37)	1.03 (0.37-2.84)
**Adults over 65 years**												
None	44787	1487196	3011.51	1.00 (ref)	1.00 (ref)	1.00 (ref)	21283	768392	2769.81	1.00 (ref)	1.00 (ref)	1.00 (ref)
Only children aged 0-11 years	541	25396	2130.27	1.03 (0.95-1.13)	0.98 (0.90-1.08)	0.98 (0.89-1.07)	230	12999	1769.38	0.95 (0.84-1.09)	0.91 (0.79-1.04)	0.91 (0.80-1.04)
Only children aged ⩾ 12 years	404	16723	2415.79	1.00 (0.91-1.11)	0.96 (0.87-1.06)	0.97 (0.87-1.07)	181	8764	2065.27	0.94 (0.81-1.09)	0.90 (0.78-1.05)	0.90 (0.77-1.04)
Children aged 0-11 and ≥12	208	8784	2367.92	1.05 (0.91-1.20)	1.00 (0.87-1.15)	1.01 (0.88-1.16)	102	4647	2194.86	1.08 (0.89-1.31)	1.03 (0.84-1.25)	1.05 (0.86-1.28)
**Number of children aged 0-11 years in household**												
1 child 0-11 years	320	15012	2131.69	1.00 (ref)	1.00 (ref)	1.00 (ref)	142	7820	1815.94	1.00 (ref)	1.00 (ref)	1.00 (ref)
2 children 0-11 years	161	8086	1991.15	0.95 (0.79-1.15)	0.99 (0.82-1.20)	1.02 (0.84-1.24)	65	4085	1591.09	0.90 (0.67-1.21)	0.91 (0.68-1.22)	0.95 (0.71-1.28)
3 children 0-11 years	53	1917	2764.89	1.26 (0.94-1.70)	1.29 (0.95-1.73)	1.26 (0.93-1.71)	17	917	1853.39	1.00 (0.60-1.66)	1.01 (0.60-1.69)	1.02 (0.61-1.72)
⩾4 children 0-11 years	7	382	1834.69	0.76 (0.36-1.63)	0.76 (0.35-1.61)	0.83 (0.38-1.79)	6	177	3394.1	1.73 (0.76-3.92)	1.75 (0.76-4.02)	1.98 (0.84-4.65)

*Demographic adjusted model: Adjusted for age, sex, ethnicity, number adults in household, IMD, BMI, smoking.
**Comorbidity-adjusted model: Adjusted for age, sex, ethnicity, number adults in household, IMD, BMI, smoking, hypertension or high blood pressure, chronic respiratory disease, asthma, cancer, chronic liver disease, stroke or dementia, other neurological disease, reduced kidney function, end-stage renal disease, solid organ transplant, asplenia, rheumatoid, lupus or psoriasis, other immunosuppressive condition.

Table A7. Hazard Ratios (HRs) for threadworm between 2018 to 2019, stratified by age

				Worms HR (95% CI)		
	**N**	**Person years**	**Rate**	**Age-sex adjusted**	**+ Demographic adjusted***	**+ Comorbidity adjusted****
**Adults 65 years and under**						
None	470	5712919	8.23	1.00 (ref)	1.00 (ref)	1.00 (ref)
Only children aged 0-11 years	508	1818356	27.94	2.73 (2.36-3.16)	2.70 (2.33-3.13)	2.70 (2.33-3.12)
Only children aged ≥ 12 years	84	782920	10.73	1.29 (1.01-1.64)	1.31 (1.03-1.68)	1.30 (1.02-1.67)
Children aged 0-11 and >12 years	120	605682	19.81	2.13 (1.70-2.67)	2.07 (1.65-2.61)	2.08 (1.65-2.62)
**Number of children aged 1-<11 years in household**						
1 child 0-11 years	180	905723	19.87	1.00 (ref)	1.00 (ref)	1.00 (ref)
2 children 0-11 years	219	708900	30.89	1.58 (1.28-1.96)	1.58 (1.28-1.96)	1.59 (1.28-1.97)
3 children 0-11 years	71	164533	43.15	2.14 (1.60-2.87)	2.10 (1.57-2.82)	2.14 (1.59-2.87)
≥4 children 0-11 years	38	39199	96.94	4.64 (3.12-6.91)	4.48 (2.99-6.69)	4.30 (2.87-6.45)
**Adults over 65 years**						
None	82	2256656	3.63	1.00 (ref)	1.00 (ref)	1.00 (ref)
Only children aged 0-11 years	6	34074	17.61	4.51 (1.66-12.25)	4.13 (1.32-12.99)	2.98 (0.69-12.87)
Only children aged ≥ 12 years	REDACTED	23454	8.53	2.27 (0.57-9.05)	2.15 (0.48-9.65)	1.13 (0.13-9.58)
Children aged 0-11 and >12 years	REDACTED	11741	8.52	2.17 (0.30-15.63)	1.89 (0.23-15.66)	1.99 (0.23-17.19)
**Number of children aged 1-<11 years in household**						
1 child 0-11 years	REDACTED	20038	9.98	1.00 (ref)	1.00 (ref)	1.00 (ref)
2 children 0-11 years	REDACTED	10951	27.4	3.02 (0.38-24.08)	2.93 (0.33-25.81)	0.79 (0.06-10.22)
3 children 0-11 years	REDACTED	2561	39.04	4.45 (0.28-69.99)	4.85 (0.27-87.30)	0.00 (0.00-0.00)
≥4 children 0-11 years	0	524	0	0.00 (.- .)	0.00 (.- .)	0.00 (0.00-0.00)

*Demographic adjusted model: Adjusted for age, sex, ethnicity, number adults in household, IMD, BMI, smoking.
**Comorbidity-adjusted model: Adjusted for age, sex, ethnicity, number adults in household, IMD, BMI, smoking, hypertension or high blood pressure, chronic respiratory disease, asthma, cancer, chronic liver disease, stroke or dementia, other neurological disease, reduced kidney function, end-stage renal disease, solid organ transplant, asplenia, rheumatoid, lupus or psoriasis, other immunosuppressive condition.

Table A8: Hazard Ratios (HRs) and 95% confidence intervals (CIs) for COVID-19 outcomes among those ≤65 years, comparing results of complete case analysis with multiple imputation to account for ethnicity.

	Evidence of SARS-CoV-2 infection in primary care		Hospital admission		ICU admission		Death from COVID-19	
	Complete case*	Multiple imputation	Complete case*	Multiple imputation	Complete case*	Multiple imputation	Complete case*	Multiple imputation
**WAVE 1**								
None	1.00 (ref)	1.00 (ref)	1.00 (ref)	1.00 (ref)	1.00 (ref)	1.00 (ref)	1.00 (ref)	1.00 (ref)
Only children aged 0-11 years	0.98 (0.96-1.01)	1.01 (0.98-1.03)	0.92 (0.85-1.00)	0.95 (0.88-1.02)	0.91 (0.76-1.07)	0.97 (0.83-1.14)	0.71 (0.57-0.90)	0.73 (0.59-0.90)
Only children aged ≥ 12 years	1.05 (1.01-1.08)	1.07 (1.04-1.10)	1.06 (0.97-1.16)	1.07 (0.99-1.16)	1.05 (0.88-1.24)	1.11 (0.95-1.29)	0.87 (0.69-1.09)	0.92 (0.75-1.12)
Children aged 0-11 and ≥12 years	1.01 (0.97-1.05)	1.04 (1.00-1.07)	1.12 (1.01-1.23)	1.14 (1.04-1.24)	1.00 (0.81-1.23)	1.06 (0.88-1.29)	0.76 (0.56-1.03)	0.79 (0.60-1.04)
**WAVE 2**								
None	1.00 (ref)	1.00 (ref)	1.00 (ref)	1.00 (ref)	1.00 (ref)	1.00 (ref)	1.00 (ref)	1.00 (ref)
Only children aged 0-11 years	1.06 (1.05-1.08)	1.06 (1.05-1.08)	1.18 (1.06-1.31)	1.23 (1.12-1.36)	1.19 (0.98-1.43)	1.22 (1.02-1.45)	0.52 (0.35-0.78)	0.62 (0.44-0.87)
Only children aged ≥ 12 years	1.22 (1.20-1.24)	1.25 (1.23-1.27)	1.26 (1.12-1.40)	1.31 (1.19-1.45)	1.13 (0.93-1.39)	1.26 (1.05-1.51)	0.97 (0.71-1.33)	0.92 (0.69-1.22)
Children aged 0-11 and ≥12 years	1.25 (1.23-1.27)	1.25 (1.24-1.27)	1.44 (1.28-1.63)	1.55 (1.39-1.73)	1.22 (0.96-1.55)	1.31 (1.05-1.62)	0.84 (0.54-1.30)	0.89 (0.60-1.32)

*Comorbidity-adjusted model: Adjusted for age, sex, ethnicity, number adults in household, IMD, BMI, smoking, hypertension or high blood pressure, chronic respiratory disease, asthma, cancer, chronic liver disease, stroke or dementia, other neurological disease, reduced kidney function, end-stage renal disease, solid organ transplant, asplenia, rheumatoid, lupus or psoriasis, other immunosuppressive condition.

Table A9: Hazard Ratios (HRs) and 95% confidence intervals (CIs) for COVID-19 outcomes among those >65 years, comparing results of complete case analysis with multiple imputation to account for ethnicity

	**Evidence of SARS-CoV-2 infection in primary care**		**Hospital admission**		**ICU admission**		**Death from COVID-19**	
	Complete case*	Multiple imputation	Complete case*	Multiple imputation	Complete case*	Multiple imputation	Complete case*	Multiple imputation
**WAVE 1**								
None	1.00 (ref)	1.00 (ref)	1.00 (ref)	1.00 (ref)	1.00 (ref)	1.00 (ref)	1.00 (ref)	1.00 (ref)
Only children aged 0-11 years	1.04 (0.93-1.16)	1.09 (0.99-1.20)	1.06 (0.91-1.24)	1.10 (0.96-1.26)	1.09 (0.71-1.67)	1.07 (0.72-1.58)	0.86 (0.70-1.06)	0.86 (0.72-1.04)
Only children aged ≥ 12 years	0.92 (0.80-1.06)	0.98 (0.86-1.10)	1.03 (0.86-1.25)	1.03 (0.87-1.22)	1.26 (0.75-2.12)	1.24 (0.77-1.99)	0.80 (0.62-1.03)	0.82 (0.72-1.04))
Children aged 0-11 and ≥12 years	1.17 (1.00-1.37)	1.19 (1.02-1.37)	1.16 (0.92-1.45)	1.15 (0.93-1.42)	0.87 (0.41-1.85)	0.91 (0.45-1.84)	0.92 (0.68-1.24)	0.93 (0.70-1.23)
**WAVE 2**								
None	1.00 (ref)	1.00 (ref)	1.00 (ref)	1.00 (ref)	1.00 (ref)	1.00 (ref)	1.00 (ref)	1.00 (ref)
Only children aged 0-11 years	1.15 (1.06-1.24)	1.28 (1.19-1.37)	0.96 (0.77-1.19)	1.09 (0.90-1.32)	1.30 (0.87-1.95)	1.38 (0.97-1.99)	1.09 (0.86-1.40)	1.13 (0.91-1.41)
Only children aged ≥ 12 years	1.16 (1.06-1.27)	1.28 (1.17-1.39)	1.04 (0.81-1.34)	1.17 (0.93-1.47)	1.04 (0.59-1.84)	0.97 (0.57-1.67)	1.14 (0.86-1.50)	1.15 (0.90-1.48)
Children aged 0-11 and≥12 years	1.27 (1.14-1.42)	1.45 (1.31-1.61)	1.19 (0.90-1.58)	1.48 (1.16-1.90)	1.86 (1.11-3.14)	2.18 (1.38-3.44)	1.44 (1.05-1.97)	1.59 (1.20-2.10)

*Comorbidity-adjusted model: Adjusted for age, sex, ethnicity, number adults in household, IMD, BMI, smoking, hypertension or high blood pressure, chronic respiratory disease, asthma, cancer, chronic liver disease, stroke or dementia, other neurological disease, reduced kidney function, end-stage renal disease, solid organ transplant, asplenia, rheumatoid, lupus or psoriasis, other immunosuppressive condition.

Table A10: Bias-adjusted hazard ratios accounting for high-risk occupation for the association between living in a household with children and Covid-19 outcome among adults aged ≤ 65 years assuming a hazard ratio between high-risk occupation and outcome of 1.3

Outcome	Exposure	Wave	Main analysis hazard ratio (95% CI)	Bias-adjusted hazard ratio (95% CI)
**Reported SARS-CoV-2 infection**	**Only children aged 0-11 years**	Wave 1	0.98 (0.96-1.01)	0.95 (0.93-0.98)
		Wave 2	1.06 (1.05-1.08)	1.03 (1.02-1.05)
	**Only children aged ≥12 years**	Wave 1	1.05 (1.01-1.08)	1.02 (0.98-1.05)
		Wave 2	1.22 (1.20-1.24)	1.19 (1.17-1.21)
	**Children aged 0-11 and ≥12 years**	Wave 1	1.01 (0.97-1.05)	0.98 (0.94-1.02)
		Wave 2	1.25 (1.23-1.27)	1.22 (1.20-1.24)
**COVID-19 hospital admission**	**Only children aged 0-11 years**	Wave 1	0.92 (0.85-1.00)	0.90 (0.83-0.97)
		Wave 2	1.18 (1.06-1.31)	1.15 (1.03-1.27)
	**Only children aged ≥12 years**	Wave 1	1.06 (0.97-1.16)	1.03 (0.94-1.13)
		Wave 2	1.26 (1.12-1.40)	1.23 (1.09-1.36)
	**Children aged 0-11 and ≥12 years**	Wave 1	1.12 (1.01-1.23)	1.09 (0.98-1.20)
		Wave 2	1.44 (1.28-1.63)	1.40 (1.25-1.59)
**COVID-19 ICU admission**	**Only children aged 0-11 years**	Wave 1	0.91 (0.76-1.07)	0.89 (0.74-1.04)
		Wave 2	1.19 (0.98-1.43)	1.16 (0.95-1.39)
	**Only children aged ≥12 years**	Wave 1	1.05 (0.88-1.24)	1.02 (0.86-1.21)
		Wave 2	1.13 (0.93-1.39)	1.10 (0.90-1.35)
	**Children aged 0-11 and ≥12 years**	Wave 1	1.00 (0.81-1.23)	0.97 (0.79-1.20)
		Wave 2	1.22 (0.96-1.55)	1.19 (0.93-1.51)
**COVID-19 death**	**Only children aged 0-11 years**	Wave 1	0.71 (0.57-0.90)	0.69 (0.55-0.88)
		Wave 2	0.52 (0.35-0.78)	0.51 (0.34-0.76)
	**Only children aged ≥12 years**	Wave 1	0.87 (0.69-1.09)	0.85 (0.67-1.06)
		Wave 2	0.97 (0.71-1.33)	0.94 (0.69-1.29)
	**Children aged 0-11 and ≥12 years**	Wave 1	0.76 (0.56-1.03)	0.74 (0.54-1.00)
		Wave 2	0.84 (0.54-1.30)	0.82 (0.53-1.26)

Table A11: Bias-adjusted hazard ratios accounting for high-risk occupation for the association between living in a household with children and Covid-19 outcomes among adults aged ≤ 65 years assuming a hazard ratio between high-risk occupation and outcome of 2

Outcome	Exposure	Wave	Main analysis hazard ratio (95% CI)	Bias-adjusted hazard ratio (95% CI)
**Reported SARS-CoV-2 infection**	**Only children aged 0-11 years**	Wave 1	0.98 (0.96-1.01)	0.91 (0.89-0.94)
		Wave 2	1.06 (1.05-1.08)	0.98 (0.97-1.00)
	**Only children aged ≥12 years**	Wave 1	1.05 (1.01-1.08)	0.97 (0.94-1.00)
		Wave 2	1.22 (1.20-1.24)	1.13 (1.11-1.15)
	**Children aged 0-11 and ≥12 years**	Wave 1	1.01 (0.97-1.05)	0.94 (0.90-0.97)
		Wave 2	1.25 (1.23-1.27)	1.16 (1.14-1.18)
**COVID-19 hospital admission**	**Only children aged 0-11 years**	Wave 1	0.92 (0.85-1.00)	0.85 (0.79-0.93)
		Wave 2	1.18 (1.06-1.31)	1.09 (0.98-1.21)
	**Only children aged ≥12 years**	Wave 1	1.06 (0.97-1.16)	0.98 (0.90-1.07)
		Wave 2	1.26 (1.12-1.40)	1.17 (1.04-1.30)
	**Children aged 0-11 and ≥12 years**	Wave 1	1.12 (1.01-1.23)	1.04 (0.94-1.14)
		Wave 2	1.44 (1.28-1.63)	1.33 (1.19-1.51)
**COVID-19 ICU admission**	**Only children aged 0-11 years**	Wave 1	0.91 (0.76-1.07)	0.84 (0.70-0.99)
		Wave 2	1.19 (0.98-1.43)	1.10 (0.91-1.32)
	**Only children aged ≥12 years**	Wave 1	1.05 (0.88-1.24)	0.97 (0.81-1.15)
		Wave 2	1.13 (0.93-1.39)	1.05 (0.86-1.29)
	**Children aged 0-11 and ≥12 years**	Wave 1	1.00 (0.81-1.23)	0.93 (0.75-1.14)
		Wave 2	1.22 (0.96-1.55)	1.13 (0.89-1.44)
**COVID-19 death**	**Only children aged 0-11 years**	Wave 1	0.71 (0.57-0.90)	0.66 (0.53-0.83)
		Wave 2	0.52 (0.35-0.78)	0.48 (0.32-0.72)
	**Only children aged ≥12 years**	Wave 1	0.87 (0.69-1.09)	0.81 (0.64-1.01)
		Wave 2	0.97 (0.71-1.33)	0.90 (0.66-1.23)
	**Children aged 0-11 and ≥12 years**	Wave 1	0.76 (0.56-1.03)	0.70 (0.52-0.95)
		Wave 2	0.84 (0.54-1.30)	0.78 (0.50-1.20)

Table A12: Bias-adjusted hazard ratios accounting for high-risk occupation for the association between living in a household with children and Covid-19 outcomes among adults aged ≤65 years assuming a hazard ratio between high-risk occupation and outcome of 3

Outcome	Exposure	Wave	Main analysis Hazard ratio (95% CI)	Bias-adjusted hazard ratio (95% CI)
**Reported SARS-CoV-2 infection**	**Only children aged 0-11 years**	Wave 1	0.98 (0.96-1.01)	0.86 (0.85-0.89)
		Wave 2	1.06 (1.05-1.08)	0.94 (0.93-0.95)
	**Only children aged ≥12 years**	Wave 1	1.05 (1.01-1.08)	0.93 (0.89-0.95)
		Wave 2	1.22 (1.20-1.24)	1.08 (1.06-1.09)
	**Children aged 0-11 and ≥12 years**	Wave 1	1.01 (0.97-1.05)	0.89 (0.86-0.93)
		Wave 2	1.25 (1.23-1.27)	1.10 (1.09-1.12)
**COVID-19 hospital admission**	**Only children aged 0-11 years**	Wave 1	0.92 (0.85-1.00)	0.81 (0.75-0.88)
		Wave 2	1.18 (1.06-1.31)	1.04 (0.94-1.16)
	**Only children aged ≥12 years**	Wave 1	1.06 (0.97-1.16)	0.94 (0.86-1.02)
		Wave 2	1.26 (1.12-1.40)	1.11 (0.99-1.24)
	**Children aged 0-11 and ≥12 years**	Wave 1	1.12 (1.01-1.23)	0.99 (0.89-1.09)
		Wave 2	1.44 (1.28-1.63)	1.27 (1.13-1.44)
**COVID-19 ICU admission**	**Only children aged 0-11 years**	Wave 1	0.91 (0.76-1.07)	0.80 (0.67-0.94)
		Wave 2	1.19 (0.98-1.43)	1.05 (0.86-1.26)
	**Only children aged ≥12 years**	Wave 1	1.05 (0.88-1.24)	0.93 (0.78-1.09)
		Wave 2	1.13 (0.93-1.39)	1.00 (0.82-1.23)
	**Children aged 0-11 and ≥12 years**	Wave 1	1.00 (0.81-1.23)	0.88 (0.71-1.09)
		Wave 2	1.22 (0.96-1.55)	1.08 (0.85-1.37)
**COVID-19 death**	**Only children aged 0-11 years**	Wave 1	0.71 (0.57-0.90)	0.63 (0.50-0.79)
		Wave 2	0.52 (0.35-0.78)	0.46 (0.31-0.69)
	**Only children aged ≥12 years**	Wave 1	0.87 (0.69-1.09)	0.77 (0.61-0.96)
		Wave 2	0.97 (0.71-1.33)	0.86 (0.63-1.17)
	**Children aged 0-11 and ≥12 years**	Wave 1	0.76 (0.56-1.03)	0.67 (0.49-0.91)
		Wave 2	0.84 (0.54-1.30)	0.74 (0.48-1.15)

Supplementary methods

Algorithm to identify households in TPP, using addresses registered in patient record

1) Get all address details for fully registered patients
- Find all permanent patient addresses that were active on 01 Feb 2020 or recorded since
- Combine the house name/number, remove punctuation (commas, full stops, apostrophes), double spaces and leading/trailing whitespace from house name/number and road
- Replace street, lane etc with the abbreviation (street -> St, lane -> Ln, place -> Pl, avenue -> Ave, road -> Rd, close -> Cl, drive -> Dr)
- Find registrations for patients that were active on or since 01 Feb 2020 (making sure the patient was alive on 01 Feb 2020)
- Filter the patient addresses based on those registrations

2) Build an Address table using the distinct address fields
- For each distinct (house name/number + road + post code), assign a unique Address_ID

3) Set the Address_ID on each individual patient address
- Join to Address table from 2) on house name/number + road + post code

4) End addresses that started before the property was sold
- Import land registry property sales data (www.gov.uk/government/statistical-data-sets/price-paid-data-downloads)
- Apply the same processing as 1) to the address fields
- Set address IDs on the land registry house sales data using the same Address table from 2) joining on house name/number + road + post code
- End all patient addresses at properties where the patient address start date was before the property was sold (under the assumption that in the majority of cases that means the occupants moved out)
- If a patient address has an unset start date (imported data), use the registration start date for this step

5) Get the latest "active" patient address per patient
- Filter to those which were active on 01 Feb 2020 or recorded since (need to do this again now we've ended some based on property sales)
- Filter to the latest per patient

6) Create the HouseholdMember table
- Insert a row for each patient address from 5), using the Address_ID as the Household_ID (one household per address)

7) Create the Household table
- Select the distinct Address_IDs as the Household_ID into the Household table (one household per address)
- Set the size based on the number of members
- Use the PotentialCareHomeAddress table to set the CareHome flag
- Check the address fields to identify NFA / Unknown addresses (e.g. postcode "ZZ99 ...", house name "NFA", road "Unknown", ...)

Table of primary care codes used as evidence of SARS-CoV-2 infection in primary care with links to codelists

Evidence	Link to OpenSAFELY codelists
Clinical diagnosis of COVID-19	https://codelists.opensafely.org/codelist/opensafely/covid-identification-in-primary-care-probable-covid-clinical-code/2020-07-16/
Positive swab test for SARS-CoV-2	https://codelists.opensafely.org/codelist/opensafely/covid-identification-in-primary-care-probable-covid-positive-test/2020-07-16/
Sequelae of COVID-19	https://codelists.opensafely.org/codelist/opensafely/covid-identification-in-primary-care-probable-covid-sequelae/2020-07-16/

Table of covariate definitions

Covariate	Link to OpenSAFELY codelists
Obesity	Body mass index (BMI) was ascertained within the 10 years prior to 1 Feb 2020 and recorded when the patient was over 16 years old. Grouped using categories derived from the World Health Organisation classification of BMI: no evidence of obesity <30 kg/m^2; obese I 30-34.9; obese II 35-39.9; obese III 40+
Smoking	Grouped into current, former and never smokers.
Ethnicity	Categorised as a five-level variable, White, Mixed, South Asian, Black or Other
Age	age groups were: 18-29, 30-39, 40-49, 50-59, 60-69, 70-79, 80+ years.
Total number of adults in the household	Categorised as 1, 2, and 3 or more.
Region	We used the Sustainability and Transformation Partnership area (STP, an NHS administrative grouping) of the patient’s general practice as a marker of region.
Shielding patients	Identified at study start as having one or more of (ever); organ transplant, renal replacement therapy, haematological cancers, non-haematological cancers, immunodeficiencies/asplenia and chronic respiratory disease.

Table of chronic comorbidities with details and links to codelists

**Comorbidity**	**Details**	**Link to OpenSAFELY codelists**	**Time frame codes searched**
Asthma	Asthma code AND no codes indicating chronic obstructive pulmonary disease	https://codelists.opensafely.org/codelist/opensafely/asthma-diagnosis/2020-04-15/	Ever diagnosed prior to study start date
Chronic Respiratory Disease	Including chronic obstructive pulmonary disease, fibrosing lung disease, bronchiectasis or cystic fibrosis	https://codelists.opensafely.org/codelist/opensafely/chronic-respiratory-disease/2020-04-10/	Ever diagnosed prior to study start date
Chronic heart disease	Including chronic heart failure, ischaemic heart disease, and severe valve or congenital heart disease likely to require lifelong follow-up	https://codelists.opensafely.org/codelist/opensafely/chronic-cardiac-disease-snomed/2020-04-08-draft/	Ever diagnosed prior to study start date
Diabetes mellitus	Defined using a combination of diabetes diagnosis codes, anti-diabetes drugs and HBA1C records were used to define diabetes status.	https://codelists.opensafely.org/codelist/opensafely/diabetes-unknown-type/2020-06-29/<br><br>https://codelists.opensafely.org/codelist/opensafely/type-1-diabetes/2020-06-29/<br><br>https://codelists.opensafely.org/codelist/opensafely/type-2-diabetes/2020-06-29/<br><br>https://codelists.opensafely.org/codelist/opensafely/antidiabetic-drugs/2020-07-16/<br><br>https://codelists.opensafely.org/codelist/opensafely/insulin-medication/2020-04-26/	Ever diagnosed prior to study start date
Chronic liver disease	Including all chronic viral hepatitis disease, signs of cirrhosis such as oesophageal varices and liver transplant (recipient only)	https://codelists.opensafely.org/codelist/opensafely/chronic-liver-disease/2020-06-02/	Ever diagnosed prior to study start date
Stroke/dementia	Diagnosis of stroke or dementia.	https://codelists.opensafely.org/codelist/opensafely/stroke-updated/2020-06-02/<br><br>https://codelists.opensafely.org/codelist/opensafely/dementia/2020-04-22/	Ever diagnosed prior to study start date

Other chronic neurological diseases	Including conditions in which respiratory function may be compromised, such as motor neurone disease, myasthenia gravis, multiple sclerosis, Parkinson's disease, cerebral palsy, quadriplegia or hemiplegia and progressive cerebellar disease.	https://codelists.opensafely.org/codelist/opensafely/other-neurological-conditions/2020-06-02/	Ever diagnosed prior to study start date
Common autoimmune diseases	Rheumatoid Arthritis, Systemic Lupus Erythematosus or psoriasis	https://codelists.opensafely.org/codelist/opensafely/ra-sle-psoriasis/2020-04-14/	Ever diagnosed prior to study start date
Solid organ transplant	Codes indicating other organ transplant (kidney is grouped with renal replacement therapy below)	https://codelists.opensafely.org/codelist/opensafely/other-organ-transplant/2020-07-15/	Ever diagnosed prior to study start date
Asplenia	Including splenectomy or a spleen dysfunction, including sickle cell disease.	https://codelists.opensafely.org/codelist/opensafely/asplenia/2020-06-02/	Ever diagnosed prior to study start date
Immunosuppression (including permanent immunodeficiency such as HIV, sickle cell disease, other immunosuppressive conditions and temporary immunodeficiency)	Other immunosuppressive conditions included human immunodeficiency virus or a condition inducing permanent immunodeficiency ever diagnosed, or aplastic anaemia or temporary immunodeficiency recorded within the last year.	https://codelists.opensafely.org/codelist/opensafely/hiv/2020-07-13/<br><br>https://codelists.opensafely.org/codelist/opensafely/permanent-immunosuppression/2020-06-02/<br><br>https://codelists.opensafely.org/codelist/opensafely/sickle-cell-disease/2020-04-14/<br><br>https://codelists.opensafely.org/codelist/opensafely/temporary-immunosuppression/2020-04-24/<br><br>https://codelists.opensafely.org/codelist/opensafely/aplastic-anaemia/2020-04-24/	Ever diagnosed prior to study start date
Cancer (haematological or non haematological)		https://codelists.opensafely.org/codelist/opensafely/haematological-cancer/2020-04-15/<br><br>https://codelists.opensafely.org/codelist/opensafely/cancer-excluding-lung-and-haematological/2020-04-15/<br><br>https://codelists.opensafely.org/codelist/opensafely/lung-cancer/2020-04-15/	Cancer was grouped by time since the first diagnosis (within the last year; between 1 and 4.9 years ago; more than 5 years ago).

Estimated GFR (eGFR)	Kidney function was ascertained from the most recent serum creatinine measurement, and was converted into eGFR using the CKD-EPI equation, with reduced kidney function grouped into eGFR 30–59.9 or <30 ml/min/1.73m^2. No serum creatinine measurement was categorized as eGFR>60mls.min/1.73m^2		Ever record prior to study start date
*End-stage renal disease includes	On dialysis or having had a kidney transplant ever.	https://codelists.opensafely.org/codelist/opensafely/dialysis/2020-07-16/<br><br>https://codelists.opensafely.org/codelist/opensafely/kidney-transplant/2020-07-15/	Ever diagnosis prior to study start date
High blood pressure or diagnosis of hypertension	A previous coded diagnosis of hypertension or the most recent recording indicating systolic blood pressure ≥140 mmHg or diastolic blood pressure ≥90 mmHg	https://codelists.opensafely.org/codelist/opensafely/hypertension/2020-04-28/	Ever diagnosis or record prior to study start date

Further details of quantitative bias analysis

We used literature estimates to specify a plausible range of values for the association between COVID-19 and high-risk occupation.

In data from the Labour Force Survey 31% of UK working-age adults with children were key workers, whereas 27% of those without children were key workers. Key workers[16] are workers who are deemed by the UK government to be essential during the COVID-19 outbreak. This grouping includes health care workers, social care workers, and others in frontline positions (i.e. police), who, as such, are likely at higher risk of COVID-19 infection.

Based on this literature we estimated the prevalence of high-risk occupation among those with and without children as 0.35 and 0.25 respectively.

We also specified a range of plausible values for the association between high-risk occupation and SARS-CoV-2infection. Among key workers, it would be anticipated that healthcare workers are at highest risk. As such we used the risk ratio between health care worker occupation and COVID-19 seroprevalence in a published Danish study (1.33, 95% CI 1.12-1.58)(Iversen et al. 2020)[17] and also the risk ratio for COVID-19 related admission to hospital between patient facing and non-patient facing healthcare workers (hazard ratio 3.30, 2.13 to 5.13)(Shah et al. 2020) to specify a range of plausible values for the association between high-risk occupation and COVID-19 infection: 1.3, 2 and 3.

We assume the effect of high-risk occupation on COVID-19 outcomes is mediated solely by risk of SARS-CoV-2 infection. In UK Office for National Statistics data there was no difference in COVID-19 mortality between health care workers and the working-age population.(Office for National Statistics 2020)

References

Iversen, Kasper, Henning Bundgaard, Rasmus B. Hasselbalch, Jonas H. Kristensen, Pernille B. Nielsen, Mia Pries-Heje, Andreas D. Knudsen, et al. 2020. “Risk of COVID-19 in Health-Care Workers in Denmark: An Observational Cohort Study.” *The Lancet Infectious Diseases*, August. https://doi.org/10.1016/S1473-3099(20)30589-2.

Office for National Statistics. 2020. “Coronavirus (COVID-19) Related Deaths by Occupation, England and Wales: Deaths Registered between 9 March and 28 December 2020.” Ons.Gov.Uk. January 25, 2020. https://www.ons.gov.uk/peoplepopulationandcommunity/healthandsocialcare/causesofdeath/bulletins/coronaviruscovid19relateddeathsbyoccupationenglandandwales/deathsregisteredbetween9marchand28december2020#related-links.

Shah, Anoop S. V., Rachael Wood, Ciara Gribben, David Caldwell, Jennifer Bishop, Amanda Weir, Sharon Kennedy, et al. 2020. “Risk of Hospital Admission with Coronavirus Disease 2019 in Healthcare Workers and Their Households: Nationwide Linkage Cohort Study.” *BMJ* 371 (October): m3582.
